# A High‐Entropy Single‐Atom Catalyst Toward Oxygen Reduction Reaction in Acidic and Alkaline Conditions

**DOI:** 10.1002/advs.202309883

**Published:** 2024-04-30

**Authors:** Mohsen Tamtaji, Min Gyu Kim, Jun WANG, Patrick Ryan Galligan, Haoyu Zhu, Faan‐Fung Hung, Zhihang Xu, Ye Zhu, Zhengtang Luo, William A. Goddard, GuanHua Chen

**Affiliations:** ^1^ Hong Kong Quantum AI Lab Limited Pak Shek Kok Hong Kong SAR 999077 China; ^2^ Beamline Research Division Pohang Accelerator Laboratory (PAL) Pohang University of Science and Technology Pohang 37673 Republic of Korea; ^3^ Department of Chemical and Biological Engineering Guangdong‐Hong Kong‐Macao Joint Laboratory for Intelligent Micro‐Nano Optoelectronic Technology William Mong Institute of Nano Science and Technology and Hong Kong Branch of Chinese National Engineering Research Center for Tissue Restoration and Reconstruction The Hong Kong University of Science and Technology Clear Water Bay Hong Kong Kowloon 999077 P.R. China; ^4^ Department of Applied Physics Research Institute for Smart Energy The Hong Kong Polytechnic University Hong Kong 999077 China; ^5^ Materials and Process Simulation Center (MSC), MC 139–74 California Institute of Technology Pasadena CA 91125 USA; ^6^ Department of Chemistry The University of Hong Kong Pokfulam Road Hong Kong SAR 999077 China

**Keywords:** DFT, electrocatalyst, overpotential, volcano plot, Zinc–air battery

## Abstract

The design of high‐entropy single‐atom catalysts (HESAC) with 5.2 times higher entropy compared to single‐atom catalysts (SAC) is proposed, by using four different metals (FeCoNiRu‐HESAC) for oxygen reduction reaction (ORR). Fe active sites with intermetallic distances of 6.1 Å exhibit a low ORR overpotential of 0.44 V, which originates from weakening the adsorption of OH intermediates. Based on density functional theory (DFT) findings, the FeCoNiRu‐HESAC with a nitrogen‐doped sample were synthesized. The atomic structures are confirmed with X‐ray photoelectron spectroscopy (XPS), X‐ray absorption (XAS), and scanning transmission electron microscopy (STEM). The predicted high catalytic activity is experimentally verified, finding that FeCoNiRu‐HESAC has overpotentials of 0.41 and 0.37 V with Tafel slopes of 101 and 210 mVdec^−1^ at the current density of 1 mA cm^−2^ and the kinetic current densities of 8.2 and 5.3 mA cm^−2^, respectively, in acidic and alkaline electrolytes. These results are comparable with Pt/C. The FeCoNiRu‐HESAC is used for Zinc–air battery applications with an open circuit potential of 1.39 V and power density of 0.16 W cm^−2^. Therefore, a strategy guided by DFT is provided for the rational design of HESAC which can be replaced with high‐cost Pt catalysts toward ORR and beyond.

## Graphical abstract

This study introduces high‐entropy single‐atom catalysts (HESAC) incorporating four metals in FeCoNiRu‐HESAC. The catalyst is predicted to have an ORR overpotential of 0.44 V, which is verified experimentally to be 0.41 V (acidic electrolyte) and 0.37 V (alkaline electrolyte). A Zinc–air battery based on this catalyst achieves an open circuit potential of 1.39 V and a power density of 0.16 W cm^−2^.

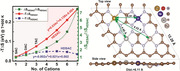

## 1 Introduction

An efficient four‐proton‐coupled electron transfer oxygen reduction reaction (ORR) is required to guarantee rapid reaction kinetics for sustainable energy conversion and storage devices such as fuel cells and metal–air batteries.^[^ ^1^, ^2^ ^]^ Single‐atom catalysts (SACs), in which the metal atoms act as the active catalytic sites for the reaction, exhibit good ORR performance^[^ ^3^, ^4^ ^]^ but suffer from scaling relationship limits.^[^ ^5^, ^6^ ^]^ Dual atom catalysts (DACs), in which one metal atom acts as the active metal site while another metal normally acts as the counterpart site, weaken the scaling relationship limit, exhibiting tunable electronic structures with unsurpassed electrocatalytic ORR activity.^[^ ^7^, ^8^, ^9^ ^]^ The counterpart metal normally acts as a modulator for the electronic structure, oxidation state, spin state, and d‐orbital electron distribution^[^ ^10^, ^11^, ^12^ ^]^ of active sites through either orbital coupling or long‐distance interactions between the two metal atoms^[^ ^5^, ^13^, ^14^ ^]^ to tune desorption and/or adsorption of reaction intermediates toward high‐performance ORR.^[^ ^15^, ^16^, ^17^, ^18^, ^19^, ^20^, ^21^, ^22^, ^23^, ^24^ ^]^ Still, the scaling relationship limit, the sluggish kinetics of the ORR processes, and the agglomeration of metals impose tremendous challenges on catalytic applications of SACs and DACs, which require the fundamental and experimental design of single‐phase electrocatalysts with enhanced ORR activity.

High entropy materials are widely used for several electrochemical reactions because of their interesting properties^[^ ^25^, ^26^ ^]^ which are synthesized by mixing several cations,^[^ ^27^, ^28^, ^29^ ^]^ and they can be oxides, carbides, nitrides, alloys, and 2D materials. However, the formation of a single‐phase and homogeneously mixed compound is a major challenge for high‐entropy materials. By incorporating several different cations, multi‐functional catalysts with several active metal sites can be designed and used for different electrochemical reactions such as an ORR, oxygen evolution reaction (OER), CO_2_ reduction reaction (CO_2_RR), nitrogen reduction reaction (NRR), nitrate reduction reaction (NO_3_RR), and hydrogen evolution reaction (HER).^[^ ^30^ ^]^ More importantly, the presence of different cations beside the active metal site can participate in the modulation of its electronic state, orbital configuration, and catalytic activity through the non‐bonding interaction, similar to DACs.^[^ ^5^, ^10^ ^]^ In other words, in HESAC, the active metal site has the possibility of possessing a new and novel environment by changing the counterpart metal atoms.^[^ ^25^, ^30^, ^31^ ^]^ This enables researchers to modify the catalytic activity of metal centers by changing the counterpart metals leading to many opportunities to design the desired catalyst toward enhanced electrocatalytic activity, surpassing the performance of SACs and DACs.

In this paper, we apply DFT calculations to predict the ORR activity of a high‐entropy single‐atom catalyst composed of Fe, Co, Ni, and Ru metals on nitrogen‐doped graphene (FeCoNiRu‐HESAC) in five different structures with averaged intermetallic distances (Dist.) of 10.68, 9.18, 6.11, 4.81, and 2.98 Å. The Fe site was found to have an ORR overpotential of 0.44 V for the intermetallic distance of 6.11 Å, significantly better than Fe, Co, Ni, and Ru SACs, which lead to overpotentials of 0.56, 0.55, 1.37, and 1.06 V, respectively. We find that the non‐bonding interactions between Fe, Co, Ni, and Ru atoms can remarkably affect their ORR activity. Inspired by the feature importance analysis obtained from machine learning (ML), we propose a new descriptor that describes the ORR performance of HESACs.

Based on our DFT calculations, we used a two‐step pyrolysis method to synthesize the FeCoNiRu‐HESAC and nitrogen‐doped graphene samples. Subsequently, the local atomic environment of the metal atoms was examined using X‐ray absorption near‐edge structure (XANES), extended X‐ray absorption fine structure (EXAFS), X‐ray photoelectron spectroscopy (XPS), and scanning transmission electron microscope (STEM). The ORR activity of the synthesized FeCoNiRu‐HESAC and nitrogen‐doped graphene samples was measured and compared with the Pt/C as the commercial benchmark in the acidic and alkaline conditions. In agreement with our theoretical predictions, we found a low experimental overpotential and high activity for FeCoNiRu‐HESAC, comparable with the ORR performance of Pt/C. These results demonstrate the feasibility of ML‐ and DFT‐guided rational design of HESACs for enhanced electrocatalytic activity, providing a new direction for state‐of‐the‐art electrocatalysts.

## 2 Results and Discussion

The concept of atomically dispersed high entropy single‐atom catalysts (HESAC) provides the opportunity of having multi‐functional catalysts with several active metal sites for enhanced electrochemical reactions. In addition, it provides the opportunity to make numerous different combinations to provide a pathway for designing new catalysts with enhanced electrochemical performance through non‐bonding interactions. For example, the number of combinations (*M*) of *n* transition metals into the catalyst with *m* (<*n*) active sites can be calculated based on Equation S1 (Supporting Information). This suggests that as the number of active sites (*m*) increases, the number of combinations (*M*) increases exponentially. For instance, considering 3d, 4d, and 5d transition metals (*n *= 30), the catalyst with four active sites (*m *= 4) leads to 27405 combinations, which is 913 times more than the 30 combinations for single‐atom catalysts (SAC) (see Table S1, Supporting Information for more details).

The successful formation of a single‐phase high‐entropy material relates directly to the values for Gibbs free energy of mixing (∆*G*_mix_) as a more stable and homogeneous mixing requires more negative values for ∆*G*_mix_, which can be calculated as:^[^ ^28^, ^29^ ^]^

(1) $$\Delta G_{\mathrm{mix}}=\Delta H_{\mathrm{mix}}-T\Delta S_{\mathrm{mix}}$$

which ∆*H*_mix_, ∆*S*_mix_, and T are the enthalpy of mixing, the entropy of mixing, and the absolute temperature (K), respectively. We describe the enthalpy of mixing, ∆H_mix_, per metal referenced to nitrogen‐doped graphene simply as:

(2) $$\Delta H_{\mathrm{mix}}=\left[E_{\mathrm{HESAC}}-E_{N-\mathrm{doped}}-\sum_{i=1}^{m}E_{i}^{\mathrm{bulk}}\right]\;/m$$

where *E*_HESAC_ and *E*_N‐doped_ are the total energies of HESAC and nitrogen‐doped systems, respectively. *E*_i_^bulk^ refers to the energy of i^th^ metal atom in its most stable bulk structure which is shown in Table S2 (Supporting Information). By increasing the number of cations of different types, the enthalpy part goes to positive values which depends on the atomic radius and electronegativity of the cations, which weakens the homogeneous mixing contribution. However, the interplay of entropy part plays a competitive role in the Gibbs free energy of mixing. The entropy of mixing for an ideal solution might be calculated based on the following equation:^[^ ^46^ ^]^

(3) $$\Delta S_{\mathrm{mix}}=-R\sum_{i\;=\;1}^{m}x_{i}\ln\left(x_{i}\right)$$

where *R* and *x*_i_ are the ideal gas constant (=0.0862 eV K^−1^ atom^−1^) and the molar concentration of i^th^ element, respectively. This indicates that by increasing the number of cations of different types, the entropy of the system goes to more negative values which in turn enhances the feasibility of single‐phase high‐entropy material formation.

**Figure 1:**
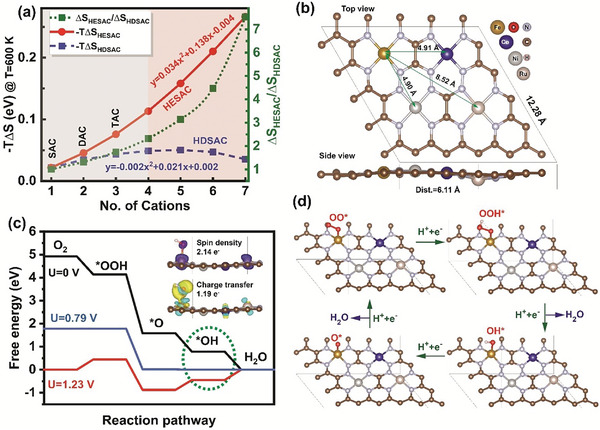
Oxygen reduction reaction (ORR) mechanism on FeCoNiRu‐HESAC. a) Entropy behavior of HDSAC and HESAC versus the number of cations in the 4 × 4 supercell of nitrogen‐doped Graphene. b) Lateral and side views of the DFT‐optimized structure of FeCoNiRu‐HESAC with the averaged intermetallic distance (Dist.) of 6.11 Å. Fe, Co, Ni, and Ru are anchored on the moiety side. c) Free energy change of ORR intermediates catalyzed by FeCoNiRu‐HESAC at the applied potentials of 0.00 V (black), 0.79 V (blue), and 1.23 V (red), indicating that the potential determining step is the desorption of OH* intermediate (shown in a dashed green cycle). The inset shows the spin and charge transfer of FeCoNiRu‐HESAC in the presence of OH* intermediate. Red and blue colors correspond to the beta and alpha spin density, respectively (Isosurface value = 0.02 e Å^−3^), while yellow and green colors indicate the charge availability and deficiency, respectively (Isosurface value = 0.005 e Å^−3^). d) Side view of ORR intermediates on Fe active site of FeCoNiRu‐HESAC.

We have investigated the entropy behavior versus the number of cations in a 4 × 4 supercell of nitrogen‐doped graphene. We assume that introducing each metal leads to two carbon vacancies and the replacement of adjacent carbon atoms with nitrogen atoms. The catalysts made from one, two, and three metals of different types are named, respectively, as single, dual, and triple atom catalysts (SAC, DAC, and TAC), and the catalysts made from four or more different equimolar metals are named as the high entropy single‐atom catalysts (HESAC). While the catalysts made from four or more metals of the same type are named as high‐density single‐atom catalysts (HDSAC). According to **Figure** **1**a, by increasing the number of cations in a 4 × 4 supercell of nitrogen‐doped graphene, the entropy of the system increases for both HESAC and HDSAC. For example, the entropy of a HESAC and HDSAC with 4 cations possess, respectively, 5.2 and 2.9 times more negative values compared to a SAC. Besides the entropy of HESAC is 1.8 times more than the entropy of HDSAC and increases by adding the cations to the 4 × 4 supercell.

Figure S3 and Table S2 (Supporting Information) show the computed enthalpy, entropy, and Gibbs free energy of mixing for HESAC with 4 cations at 600 K (see experimental section) for various intermetallic distances (Dist.). This indicates that by decreasing the intermetallic distances the enthalpy of mixing decreases, while the entropy of mixing reaches −0.11 eV, comparable with the entropy for SAC (−0.02 eV at 600 K) and high‐entropy spinal oxide (−0.14 eV at 1000 K). For all the samples, the Gibbs free energy of mixing is negative which indicates the thermodynamic stability of the catalysts. It is noteworthy to highlight that, as stated in the literature, Co, Ni, and Ru atoms have been employed in the form of FeCo‐DAC,^[^ ^5^ ^]^ FeNi‐DAC,^[^ ^10^ ^]^ and FeRu‐DAC^[^ ^47^ ^]^ to modulate the electrocatalytic activity of Fe site. In this study, we have taken a similar approach by incorporating Co, Ni, and Ru metals simultaneously to enhance the activity of the Fe site. Hence, we have utilized Fe, Co, Ni, and Ru metals as the four cations in our work.

We then consider the electrochemical stability of HESAC by calculating formation energy (*E*_f_) and dissociation potential (*U*_diss_) which are defined as follows:^[^ ^13^ ^]^

(4) $$E_{f}=\Delta G_{\mathrm{mix}}\;\mathrm{and}\;U_{\mathrm{diss}}=U_{\mathrm{diss}}^{o}\;\left(M-\mathrm{bulk}\right)-\frac{E_{f}}{ne}$$

$U_{\mathrm{diss}}^{o}\left(M-\mathrm{bulk}\right)$ and *n* are the standard dissolution potential of the bulk metals and the number of electrons involved in the dissolution process, respectively. Table S3 (Supporting Information) shows the computed dissociation potential (*U*_diss_, V) of Fe, Co, Ni, and Ru metals from FeCoNiRu‐HESAC with an averaged intermetallic distance (Dist.) of 6.11 Å. The positive values for *U*_diss_ implies the electrochemical stability of the understudied catalyst.

Subsequently, to investigate the electrochemical activities, we calculated the Gibbs free energy (Δ*G*) of ORR intermediates for FeCoNiRu‐HESAC in planar and reversed sandwich structures with the average intermetallic distances (Dist.) of 10.68, 9.15, 6.11, 4.81, and 2.93 Å. The average intermetallic distances are calculated based on the DFT‐relaxed structure as Dist. = (Dist._Fe‐Co_+ Dist._Fe‐Ni_ + Dist._Fe‐Ru_)/3. Figure 1b and Figure S2 (Supporting Information) show the top and lateral views of the optimized structural models of inversed sandwich and planar structures. ORR electrocatalysis on the metal active sites proceeds through the four fundamental steps shown in Schematic S1 (Supporting Information). Due to the difficulties associated with determining the accurate free energy of OH, O, OOH, OO, and H radicals in the electrolyte solution, Δ*G*_OH*_, Δ*G*_O*_, Δ*G*_OOH*_, Δ*G*_OO*,_ and Δ*G*_H*_ are calculated with reference to the free energy of stoichiometrically appropriate quantities of H_2_ (g) and H_2_O (g), at zero potential (*U* = 0 V_RHE_) as follows:^[^ ^3^ ^]^

(5) $$\begin{array}{ccc} \Delta G_{OH^{*}} & = & E_{\mathrm{HESAC}-OH^{*}}\;-E_{\mathrm{HESAC}}+\frac{1}{2}E_{H_{2}}\\ & & -\;E_{H_{2}O}+\Delta E_{\mathrm{ZPE}}-T\Delta S+\Delta E_{\mathrm{solv}.} \end{array}$$

(6) $$\begin{array}{ccc} \Delta G_{O^{*}} & = & E_{\mathrm{HESAC}-O^{*}}\;-E_{\mathrm{HESAC}}+E_{H_{2}}-E_{H_{2}O}\\ & & +\;\Delta E_{\mathrm{ZPE}}-T\Delta S+\Delta E_{\mathrm{solv}.} \end{array}$$

(7) $$\begin{array}{ccc} \Delta G_{\mathrm{OO}H^{*}} & = & E_{\mathrm{HESAC}-\mathrm{OO}H^{*}}\;-E_{\mathrm{HESAC}}+\frac{3}{2}E_{H_{2}}-2E_{H_{2}O}\\ & & +\;\Delta E_{\mathrm{ZPE}}-T\Delta S+\Delta E_{\mathrm{solv}.} \end{array}$$

(8) $$\begin{array}{ccc} \Delta G_{O_{2}^{*}} & = & E_{\mathrm{HESAC}-O_{2}^{*}}\;-E_{\mathrm{HESAC}}+2E_{H_{2}}\\ & & -\;2E_{H_{2}O}+\Delta E_{\mathrm{ZPE}}-T\Delta S+\Delta E_{\mathrm{solv}.} \end{array}$$

(9) $$\begin{array}{ccc} \Delta G_{H^{*}} & = & E_{\mathrm{HESAC}-H^{*}}\;-E_{\mathrm{HESAC}}\\ & & -\;0.5E_{H_{2}}+\Delta E_{\mathrm{ZPE}}-T\Delta S+\Delta E_{\mathrm{solv}.} \end{array}$$

$E_{H_{2}O}$ and $E_{H_{2}}$ are the energy of H_2_O (g) and H_2_ (g), respectively. E_HESAC_, E_HESAC‐OH*_, E_HESAC‐O*_, E_HESAC‐OOH*_, E_HESAC‐OO*_, and E_HESAC‐H*_ are the total energy of HESAC without and with adsorbed OH*, O*, OOH*, OO*, and H*, respectively. Δ*E*_ZPE_ is zero‐point energy (ZPE) correction obtained from the vibrational calculation, Δ*S* is entropy contribution calculated using a harmonic oscillator approximation, T is the absolute temperature (K), and Δ*E*_solv._ is the solvation energy. The heatmap of Gibbs free energies at zero potential (U = 0 V) for all the metals in different structures is shown in Figure S4 (Supporting Information). The changes in the Gibss free energies are generally defined as ΔG (U, pH) =  ΔG(0) + ΔG_U_ + ΔG_pH_. In this work, the Gibbs free energies remain relatively constant regardless of the pH of the solution and linearly decreases with the applied potential ($\Delta G_{OH^{*}}\;\left(U\right)=\Delta G_{OH^{*}}\left(0\right)-\mathrm{eU}$, $\Delta G_{O^{*}}\;\left(U\right)=\Delta G_{O^{*}}\left(0\right)-2\mathrm{eU}$, and $\Delta G_{\mathrm{OO}H^{*}}\;\left(U\right)=\Delta G_{\mathrm{OO}H^{*}}\left(0\right)-3\mathrm{eU}$), which *e* is the elementary charge transferred.^[^ ^48^ ^]^ Figure 1c and Figure S8 (Supporting Information) illustrate the Gibbs free energy diagram of Fe, Co, Ni, and Ru metals in FeCoNiRu‐HESAC with Dist. = 6.11 Å (see Figure S7, Supporting Information for Gibbs free energy diagram of FeN4‐SAC and Pt(111).^[^ ^3^ ^]^ Figure 1c shows the limiting potential of 0.79 and the overpotential of |1.23‐0.79| = 0.44 V for the Fe site. Figure 1c shows that the ORR potential determining step (shown as a green dashed circle) is the formation of H_2_O from OH* as this reaction step is flat in free energy. It is worth indicating that based on Figure S8 (Supporting Information), the overpotential for Co, Ni, and Ru metals in FeCoNiRu‐HESAC are 1.00, 1.46, and 0.74 V, respectively. Therefore, the Co, Ni, and Ru sites possess unfavorable theoretical overpotentials and simply serve as counterparts to modify the electrocatalytic activity of the Fe site through long‐distance interactions. This is in agreement with the experimental works published in the literature.^[^ ^5^, ^48^ ^]^ Figure 1d shows the lateral view of ORR intermediates on th Fe site of FeCoNiRu‐HESAC with Dist. = 6.11 Å, indicating the non‐bonding effect of Co, Ni, and Ru countrerpart metals on the Fe electrocatalytic activity.

**Figure 2:**
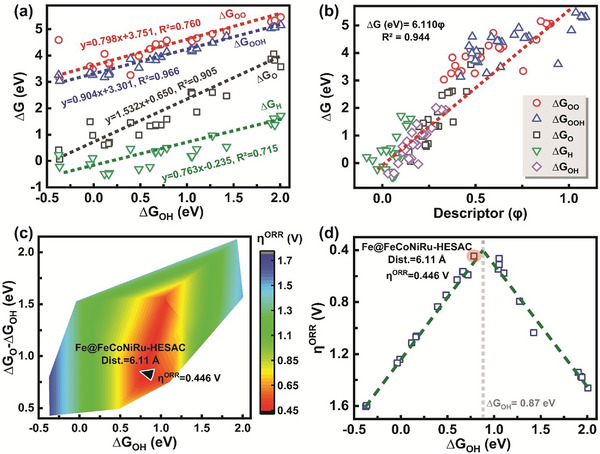
Gibbs free energy and overpotential. a) Linear relationship of ∆*G*_H*_, ∆*G*_OH*_, ∆*G*_O*_, ∆*G*_OOH*_, and ∆*G*_OO*_ vs. a) ∆*G*_OH*_ and b) new descriptor (*φ*) for FeCoNiRu‐HESAC, indicating that the new descriptor effectively establishes a linear relationship with Gibbs free energies. c) The counter plot of ORR overpotential vs. ∆*G*_OOH*_ and ∆*G*_O*_‐∆*G*_OH*_. d) Volcano plot of ORR overpotential vs. ∆*G*_OH*_, indicating Fe active site with D = 6.11 Å at the summit.

**Figure** **2**a indicates a linear relationship of ∆*G*_H*_, ∆*G*_OH*_, ∆*G*_O*_, ∆*G*_OOH*_, and ∆*G*_OO*_ vs. ∆*G*_OH*_, indicating the scaling relationship limits are weakened compared to SACs.^[^ ^49^ ^]^ Figure 2b indicates the linear relationship of ∆*G*_H*_, ∆*G*_OH*_, ∆*G*_O*_, ∆*G*_OOH*_, and ∆*G*_OO*_ vs. new descriptor (φ) for FeCoNiRu‐HESAC, indicating that the following descriptor effectively defines the linear relationships for FeCoNiRu‐HESAC:

(10) $$\begin{array}{ccc} \varphi & = & \theta_{d,\;M}\;\frac{EN_{M}\left|N_{O,\;\mathrm{int}}VE_{O}-2N_{H,\;\mathrm{int}}VE_{H}\right|}{\mathrm{At}R_{M}}\\ & & -\;\frac{1}{\mathrm{At}N_{M}}\times \frac{\mathrm{Dist}._{\mathrm{SAC}}}{\mathrm{Dist}.} \end{array}$$

θ_d,M_, EN_M_, AtR_M_, AtN_M_, Dist._SAC_, and Dist. are the number of electrons in the d orbital of metal (M), the electronegativity of M, the atomic radius of M, the atomic number of M, the intermetallic distances for single‐atom catalysts (=15 Å), and the intermetallic distances for HESAC. N_O,int_ and N_H,int_ are the number of oxygen and hydrogen atoms, respectively, in the intermediate, while VE_O_, and VE_H_ are the valence electrons of oxygen (6) and hydrogen (1) atoms, respectively. Figure 2c shows the counter plot of ORR overpotential versus ∆*G*_OOH*_ and ∆*G*_O*_‐∆*G*_OH*_, while Figure 2d demonstrates the volcano plot of ORR overpotential versus ∆*G*_OH*_, indicating Fe active site at the summit. We found that an Fe site with Dist. = 6.11 Å possesses an η^ORR^ of 0.44 V, which is comparable with DFT‐predicted overpotentials of 0.56 V for FeN4‐SAC, 0.43 V for the Pt(111),^[^ ^3^ ^]^ 0.48 V for FeNiN8‐DAC,^[^ ^5^ ^]^ 0.34 for CoCuN_6_‐gra(OH),^[^ ^19^ ^]^ and 0.33 V for CoRu@N_8_V_4_.^[^ ^50^ ^]^

**Figure 3:**
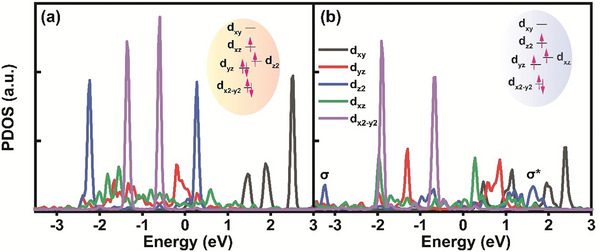
The density of states and molecular orbital energies. Partial density of states (PDOS) of 3d_x2‐y2_, 3d_xz_, 3d_z2_, 3d_yz_, and 3d_xy_ orbitals of Fe atom in FeCoNiRu‐HESAC with Dist. = 6.11 Å a) before and b) after interaction with OH*. This suggests the changes in the *z*‐axis d orbitals (3d_xz_, 3d_z2_, and 3d_yz_) where the hybridization takes place with *p*_x_ orbital of OH*. The insets show the molecular 3d orbital energy distribution of the Fe atom before and after the adsorption of OH*.

We provided the projected density of states (PDOS) in order to further investigate the impact of Co, Ni, and Ru metals on the ORR performance of the Fe site. **Figure** **3**a,b demonstrate PDOS of 3d_x2‐y2_, 3d_xz_, 3d_z2_, 3d_yz_, and 3d_xy_ orbitals of Fe atom in FeCoNiRu‐HESAC with Dist. = 6.11 Å before and after interaction with the OH* intermediate. This suggests that changes in the *z*‐axis d orbitals (3d_xz_, 3d_z2_, and 3d_yz_) where the hybridization takes place with p_x_ orbital of OH*. The insets show the molecular orbital distribution of 3d hybrid orbital of Fe before and after the adsorption of OH*, suggesting the spin crossover throughout the ORR pathway. Figure S14 (Supporting Information) shows the density of states (DOS) for Fe metal in FeCoNiRu‐HESAC with Dist. = 6.11 Å, implying the presence of antibonding (σ*) and bonding (σ) orbitals after the adsorption of OH*.^[^ ^33^ ^]^

Figure S19a (Supporting Information) shows the parity plot of ML‐ versus DFT‐obtained ∆*G* of OO*, OOH*, O*, OH*, and H* intermediates for FeCoNiRu‐HESAC. The trained ML algorithm results in satisfactory root mean square error (RMSE) and *R* ^2^ values for test and training data without any signs of over‐ and under‐fitting. Figure S19b (Supporting Information) shows feature importance values obtained from the permutation and SHAP methods along with the corresponding Mutual Information (MI) and Pearson correlation coefficients. This suggests the new descriptor (φ, Equation 10), valence electron of intermediate (VE_int_), and number of electrons in the d orbital (θ_d_) as the most informative and important parameters. In addition, these input features possess the highest Pearson coefficient and MI values, indicating the linear relationship between these features with the Gibbs free energies. Figure S19c (Supporting Information) displays a heatmap illustrating the SHAP values of the input features for all 120 data instances, arranged based on their feature importance in descending order, indicating the high effect of Ru atoms on the adsorption energies. In Figure S19d (Supporting Information), the SHAP values of the new descriptor (φ) are plotted versus the values of the new descriptor (φ), with the colors indicating the features’ values, indicating nearly linear dependence of SHAP value to the new descriptor (φ).

**Figure 4:**
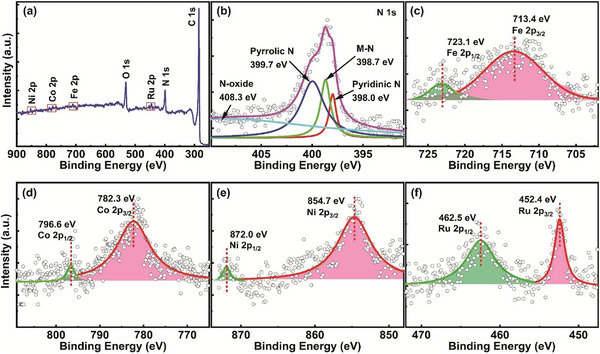
XPS characterization. a) Wide range and b–f) high‐resolution XPS spectra of N 1s, Fe 2p, Co 2p, Ni 2p, and Ru 2p for the synthesized FeCoNiRu‐HESAC sample. The binding energy observed for Fe, Co, Ni, and Ru metals suggests the presence of oxidation states higher than 0.

Inspired by our DFT calculations, we synthesized FeCoNiRu‐HESAC and nitrogen‐doped graphene samples. The compositions and chemical states of the metal atoms were investigated using X‐ray photoelectron spectroscopy (XPS), resulting in the Fe, Co, Ni, and Ru metal contents of, respectively, 0.34, 0.35, 0.33, and 0.4 wt.% in the FeCoNiRu‐HESAC (**Figure** **4**a). This observation suggests that the Fe:Co:Ni:Ru molar ratio is ≈1:1:1:1. Different types of nitrogen atoms in the FeCoNiRu‐HESAC and nitrogen‐doped graphene samples were identified, by deconvoluting the N 1s peak (Figure 4b; Figure S21b, Supporting Information). Figure 4b indicates that the deconvoluted N 1s peak for FeCoNiRu‐HESAC is composed of pyridinic‐N (398.0 eV), pyrrolic‐N (399.7 eV), Fe‐, Co‐, Ni‐, and Ru‐N (398.7 eV), and oxidized‐N (408.3 eV).^[^ ^33^ ^]^ Figure 4c–f represents high‐resolution peak spectra for Fe, Co, Ni, and Ru atoms, suggesting p_1/2_ and p_3/2_ valence states for metals with partially oxidative states.^[^ ^10^, ^48^ ^]^ These findings align with the charge analysis of Fe, Co, Ni, and Ru atoms in the relaxed structure of FeCoNiRu‐HESAC, suggesting the atomic charges of 0.587, 0.550, 0.403, and 0.171, respectively.

**Figure 5:**
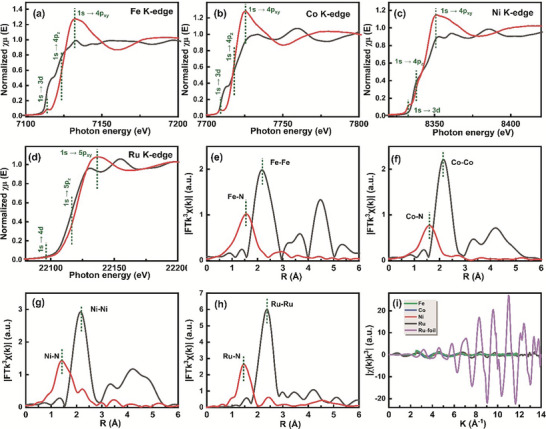
XAS characterizations. a) Fe K‐edge, b) Co K‐edge, c) Ni K‐edge, and d) Ru K‐edge XANES spectra of FeCoNiRu‐HESAC sample and their corresponding bulk samples. Fourier transformations (FT) EXAFS spectra in R space of e) Fe, f) Co, g) Ni, and h) Ru for FeCoNiRu‐HESAC sample and their corresponding bulk samples. i) The EXAFS analysis of FeCoNiRu‐HESAC sample at k space for Fe, Co, Ni, and Ru elements along with Ru foil.

The atomic configuration and electronic states of the Fe, Co, Ni, and Ru metal atoms in the FeCoNiRu‐HESAC sample are explored with the XANES and EXAFS in R and k spaces. **Figure** **5**a–d represents the Fe, Co, Ni, and Ru K‐edge XANES spectra for FeCoNiRu‐HESAC and their corresponding bulk samples, suggesting that the local atomic environment surrounding the Fe, Co, Ni, and Ru metals differs from their corresponding bulk metals. This distinction becomes more apparent when examining the first and second derivative curves of Fe, Co, Ni, and Ru K‐edge XANES spectra (Figure S27 and S28, Supporting Information). This indicates intermediate oxidation states for each single metal, which is consistent with the XPS characterization and DFT results. Figure 5a–d shows a huge blue‐shift in the absorption edge of Fe, Co, Ni, and Ru metals when compared to their respective bulk metals, implying that the valence state of Fe, Co, Ni, and Ru metals in FeCoNiRu‐HESAC is higher than their valence state in their bulk forms (=0). The higher valence states observed in the Fe, Co, Ni, and Ru metals are attributed to the coordination of Fe, Co, Ni, and Ru metals with nitrogen atoms, which forms FeN4, CoN4, NiN4, and RuN4 coordination.^[^ ^33^, ^34^, ^51^ ^]^ For the purpose of comparison, theoretical K‐edge XANES (shown in Figure S30, Supporting Information) is also obtained based on the DFT‐optimized structure shown in Figure 1b. The blue‐shift in the experimental K‐edge XANES spectra is consistent with the theoretically obtained K‐edge XANES spectra. Also, the experimental XANES spectra matches with the theoretical XANES spectra presented in Figure S30 (Supporting Information), which is based on the homogeneously dispersed mixed four atoms shown in Figure 1b. Therefore, we may say that four different atoms are homogeneously mixed in the nitrogen‐doped graphene matrix. According to Figure S30 (Supporting Information), the theoretically obtained Fe K‐edge XANES spectra of FeCoNiRu‐HESAC are between the K‐edge XANES spectra of Fe_2_O_3_ and Fe foil, indicating the oxidation state between +2 and 0. Moreover, three peaks for Fe, Co, and Ni metals in Figure 5a–c represent 1s→3d, 1s→4p_z_, and 1s→4p_xy_ transitions while three peaks for Ru metal in Figure 5d represent 1s→4d, 1s→5p_z_, and 1s→5p_xy_ transitions.^[^ ^34^, ^51^ ^]^ The variations in intensity observed in the main edge peak of 1s→4/5p_xy_ transition in Figure 5a–d are corroborated by the presence of divacancy‐based M‐N4C4 moieties that are bonded axially, resulting in broken D4h symmetry.^[^ ^33^ ^]^ However, the spin density shown in Figure S12 (Supporting Information), suggests unpaired electron delocalization from the high‐lying d_z2_ orbital of metals into ligands,^[^ ^52^ ^]^ which can result in a slight decrease in the main peak intensity of K‐edge XANES spectra of FeCoNiRu‐HESAC. In addition, Figure S32 (Supporting Information) displays the main peak intensity of XANES spectra versus the atomic number of Fe, Co, Ni, and Ru metals of FeCoNiRu‐HESAC obtained experimentally and theoretically. This indicates that by increasing the atomic number, the peak intensity decreases.

Moreover, the EXAFS Fourier transform (FT) was used to investigate the bonding and coordination environment surrounding Fe, Co, Ni, and Ru metals. In Figure 5e–h, the EXAFS spectra of Fe, Co, Ni, and Ru atoms reveal prominent primary peaks that correspond to Fe─N, Co─N, Ni─N, and Ru─N bonding at ≈1.45 Å, distinct from Fe─Fe, Co─Co, Ni─Ni, and Ru─Ru peaks at ≈2.20 Å as well as Fe─O, Co─O, Ni─O, and Ru─O peaks at ≈1.55–1.65 Å.^[^ ^48^, ^53^ ^]^ This distinction is more evident from its first derivative curves shown in Figure S29 (Supporting Information). Figure 5i displays the EXAFS analysis of the FeCoNiRu‐HESAC sample at k space for Fe, Co, Ni, and Ru elements along with Ru foil. This suggests that EXAFS analysis of metals in k space is hugely different from the bulk Ru metal.

**Figure 6:**
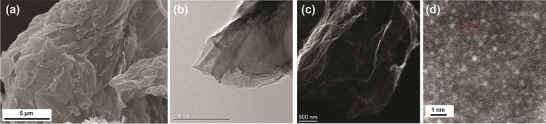
SEM, TEM, and STEM characterizations. a) SEM image, b) TEM image, and c,d) STEM images of the FeCoNiRu‐HESAC sample indicating that the individual Fe, Co, Ni, and Ru metals (white dots) are dispersed homogeneously on the nitrogen‐doped graphene support without any sign of aggregation.

The surface morphology, structure, and elemental mapping of FeCoNiRu‐HESAC are studied by SEM, TEM, and STEM images. **Figure** **6**a and Figure S23 (Supporting Information) present SEM imaging and EDX elemental mapping for C, N, Fe, Co, Ni, and Ru elements, suggesting the homogeneous dispersion of all elements. Figure 6d displays the STEM image of the FeCoNiRu‐HESAC sample, indicating a uniform distribution of metals (white dots) across the entire support. In addition, the average intermetallic distance of 6.07 Å is obtained from Figure 6d which surprisingly matches with the DFT‐obtained intermetallic distance of 6.11 Å. Figure 6b–c demonstrate the TEM and STEM images of FeCoNiRu‐HESAC, indicating that there is not any noticeable aggregation or metal clusters on the surface. These findings are in agreement with the XRD spectrum shown in Figure S22 (Supporting Information) for FeCoNiRu‐HESAC, nitrogen‐doped, and Graphene Oxide (GO) samples. The XRD spectra for crystalline GO show a sharp peak (001) at 2θ = 9.6° corresponding to the interlayer spacing of d = 9.2 Å and a tiny peak (100) at 2θ = 41.5° corresponding to the d spacing of 2.2 Å. After the annealing process, the XRD spectra for amorphous FeCoNiRu‐HESAC and nitrogen‐doped samples exhibit a single broad graphitic carbon peak (002) at 2θ = 23.8°, corresponding to an interlayer spacing of d = 3.7 Å. This decrease in the interlayer spacing arises because of the removal of OH functional groups from GO during the annealing process. In both samples, the observed broad graphitic peak can be attributed to the polycrystalline structure, and no additional peaks associated with metal clusters or their oxides or nitrides compounds are detected.^[^ ^33^, ^54^ ^]^ The Raman spectrum provided in Figure S24 (Supporting Information) suggests a rich porous support. As shown in Figure S24 (Supporting Information), two main Raman bands for GO, nitrogen‐doped, and FeCoNiRu‐HESAC are appeared at around 1353 cm^−1^ (G band) and 1583 cm^−1^ (D band), which represent, respectively, the planar configuration sp^2^ and sp^3^ bonded carbon atoms. The intensity ratio of the D‐band to the G‐band (I_D_/I_G_) in the Raman spectra demonstrates an increase from 0.54 for GO to 0.87 for the nitrogen‐doped sample, and further to 0.92 for the FeCoNiRu‐HESAC sample. Therefore, the density of defect vacancies (n_D_, Equation S11, Supporting Information) demonstrates an increase from 0.0163 nm^−2^ for GO to 0.0263 nm^−2^ for nitrogen‐doped, and further to 0.0278 nm^−2^ for FeCoNiRu‐HESAC while the distance between the defects ($\ell \left(Å\right)\;=\;44\left(I_{G}/I_{D}\right)$) decreases from 81.5 Å for GO to 50.6 Å, for nitrogen‐doped, and 47.8 Å for FeCoNiRu‐HESAC (see Supporting Information). The expected rise in defect density and decrease in the inter‐defect spacing might be because of the presence of nitrogen doping, greater disruption of the hexagonal lattice, and the addition of Fe, Co, Ni, and Ru metallic species, (see Supporting Information).^[^ ^55^ ^]^ Figure S26 (Supporting Information) shows the 1/d obtained from XRD versus I_D_/I_G_ obtained from Raman spectroscopy for GO, nitrogen‐doped graphene, and FeCoNiRu‐HESAC along with FeNiN8‐DAC, FeCoN8‐DAC, and nitrogen‐doped graphene from ref. [5] and HESAC, Fe‐SAC, and nitrogen‐doped graphene from ref. [30]. The increase in I_D_/I_G_ indicates a decrease in the d spacing.^[^ ^56^ ^]^ Figure S26 (Supporting Information) indicates a blue‐ and red‐shift, respectively, in the D and G bands of nitrogen‐doped and FeCoNiRu‐HESAC compared to GO, suggesting a decrease in the crystallinity after the annealing process which agrees with the XRD results.

**Figure 7:**
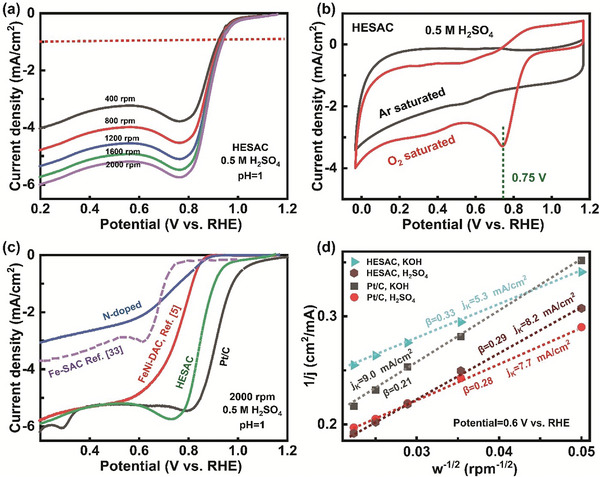
ORR performance. a) LSV polarization curves of FeCoNiRu‐HESAC in the acidic (0.5 m H_2_SO_4_) electrolyte for various rotation speeds of 400, 800, 1200, 1600, and 2000 rpm. b) CV curves obtained in Ar‐ and O_2_‐saturated acidic electrolyte, showing a prominent ORR peak at 0.75 V_RHE_ representing the reduction of O_2_ with the FeCoNiRu‐HESAC. c) LSV polarization curves of Pt/C, FeCoNiRu‐HESAC, Fe‐SAC (ref. [34]), FeNi‐DAC (ref. [5]), and nitrogen‐doped samples in the acidic (0.5 m H_2_SO_4_) electrolyte for the rotation speed of 2000 rpm. d) K–L plot at the applied potential of 0.6 V_RHE_ for FeCoNiRu‐HESAC and Pt/C samples in the acidic (0.5 m H_2_SO_4_) and alkaline (0.1 m KOH) electrolytes, with satisfactory linearity and comparable kinetic current densities (j_K_). All LSV polarization curves are obtained in an O_2_‐saturated electrolyte.

The ORR performance of synthesized FeCoNiRu‐HESAC, nitrogen‐doped graphene, and commercial Pt/C was determined using rotating disk electrode tests in 0.1 m KOH as well as 0.5 m H_2_SO_4_ electrolytes. **Figure** **7a** and Figure S34 (Supporting Information) show the linear sweep volumetry (LSV) polarization curves of FeCoNiRu‐HESAC in acidic and alkaline electrolytes for rotation speeds of 400, 800, 1200, 1600, and 2000 rpm. As shown in Figure 7a and Figure S34d (Supporting Information), the reduction current appears at 0.92 and 0.96 V_RHE_ and in acidic and alkaline electrolytes, respectively, corresponding to low overpotentials of 0.41 and 0.37 V_RHE_, which is very close to our DFT‐predicted overpotential of 0.44 V_RHE_. Figure 7b shows the Cyclic voltammetry (CV) curves in both Ar‐ and O_2_‐saturated environments, revealing a distinct ORR peak at 0.75 V_RHE_ indicating that FeCoNiRu‐HESAC is capable of reducing O_2_. Figure 7c compares the LSV polarization curves of FeCoNiRu‐HESAC, Pt/C, Fe‐SAC,^[^ ^34^ ^]^ FeNi‐DAC,^[^ ^5^ ^]^ and nitrogen‐doped samples in acidic (0.5 m H_2_SO_4_) electrolyte for the rotation speed of 2000 rpm. It is observed that our FeCoNiRu‐HESAC has a better onset potential and ORR current density compared to Fe‐SAC,^[^ ^34^ ^]^ FeNi‐DAC,^[^ ^5^ ^]^ and nitrogen‐doped samples. The Tafel plots are generated based on the LSV curves for FeCoNiRu‐HESAC (Figure S35, Supporting Information). FeCoNiRu‐HESAC leads to Tafel slopes of 210 and 101 mV dec^−1^, respectively, in acidic and alkaline electrolytes comparable with the Tafel slopes of 129 mV dec^−1^ and 137 for Pt/C, respectively, in acidic and alkaline electrolytes. Figure 7d shows the K–L plots for FeCoNiRu‐HESAC and Pt/C samples in the acidic and alkaline electrolytes at the applied potential of 0.6 V_RHE_, suggesting satisfactory linearity with comparable kinetic current densities (*j_K_*). In addition, the number of electron transfers (*n*) and electron‐transfer rate (*k*) are obtained from equation S1 and presented through Table S7 (Supporting Information). Based on Table S7 and Figure 7d, we obtain values of *n *= 3.28 e, *k *= 0.026, *j_k _* = 8.2, and *B *= 0.29 (in the acidic medium) suggesting a proficient electron transfer process for the designed FeCoNiRu‐HESAC. These values are comparable with the values of *n *= 3.16 e, *k *= 0.025, *j_k _* = 7.7, and *B *= 0.28 for a commercial Pt/C sample.

In order to address the old and important stability issue, we performed a cycling stability test for FeCoNiRu‐HESAC in O_2_‐saturated electrolytes (0.5 m H_2_SO_4_). As displayed in Figure S39 (Supporting Information), after 1000 cycles at the rotation speed of 2000 rpm, the FeCoNiRu‐HESAC possesses a very close onset potential, with a small decrease in the current density, suggesting its satisfactory stability. In addition, we have performed XRD characterization from the FeCoNiRu‐HESAC sample after 1000 ORR cycles. Figure S40 (Supporting Information) shows the XRD pattern of the FeCoNiRu‐HESAC sample before and after 1000 ORR cycles at the rotation speed of 2000 rpm. After 1000 ORR cycles, no peak is detected that would correspond to metal aggregation or their oxides and nitrides compounds. It implies that FeCoNiRu‐HESAC possesses a durable and stable atomically dispersed structure.

**Figure 8:**
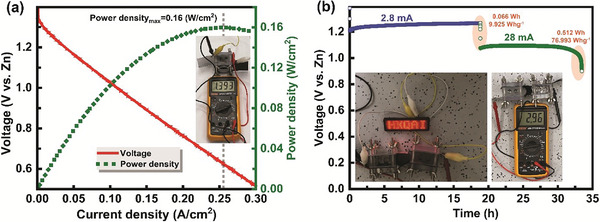
Zinc–air battery performance. a) Polarization curve and corresponding power density plots of Zinc–air battery with FeCoNiRu‐HESAC in place of cathodes. The inset shows the photograph of the assembled Zinc–air battery with an open‐circuit voltage of 1.393 V. b) Galvanostatic discharge plot of assembled Zinc–air battery. The insets show the photograph of a 3.0 V LED panel powered by two series‐connected Zinc–air batteries with an open‐circuit voltage of 2.96 V, displaying the ‘HKQAI’.

The performance of our designed catalyst was also tested in a Zinc–air battery setup shown in **Figure** **8**. Figure 8a and Figure S37 (Supporting Information) show the polarization curve and power density plot of a Zinc–air battery using FeCoNiRu‐HESAC or Pt/C in place of the cathode. The insets show the photograph of the Zinc–air battery assembled by FeCoNiRu‐HESAC with open‐circuit voltages of 1.393 and 1.365 V, respectively. This indicates that FeCoNiRu‐HESAC can give a power density of 0.160 W cm^−2^ at a current density of 0.255 A cm^−2^, which is comparable with the power density of 0.157 W/cm^2^ for Pt/C. Figure 8b and Figure S38 (Supporting Information) show the galvanostatic discharge plots of the Zinc–air battery with FeCoNiRu‐HESAC in place of cathode. The insets of Figure S38 (Supporting Information) show the photograph of the cathode and Zinc anodes after current densities of 2.8 and 28 mA. After 34 h, the Zinc plate is totally dissolved into the electrolyte, without any decrease in catalyst activity, indicating the stability of FeCoNiRu‐HESAC. We have demonstrated that a series‐connected configuration of Zinc–air batteries can effectively provide power to illuminate a light‐emitting diode (LED). The insets of Figure 8b features a photograph showcasing a red‐color LED panel (3.0 V), powered by two series‐connected Zinc–air batteries with an open‐circuit voltage of 2.96 V, displaying the ‘HKQAI’.

## 3 Conclusion

In order to address the sluggish kinetics of the ORR processes, we present the rational design of nitrogen‐doped graphene‐supported high‐entropy single‐atom catalysts (HESAC) with 5.2 times more entropy compared to single‐atom catalysts (SAC) for ORR. This is accomplished by combining fundamental theory and experimental studies. We show that the non‐bonding effect of Fe, Co, Ni, and Ru metal atoms can synergistically modulate the catalytic activity of the Fe site in FeCoNiRu‐HESAC system. Our ML analysis predicts that Fe active sites exhibit an ORR overpotential of 0.44 V_RHE_ and that Ru acts as the most important metal counterpart. Inspired by the density functional theory (DFT) and ML findings, we prepared FeCoNiRu‐HESAC along with a nitrogen‐doped sample and confirmed the atomic structures with X‐ray photoelectron spectroscopy (XPS), scanning transmission electron microscopy (STEM), and X‐ray absorption (XAS). By conducting catalytic activity tests, we find that FeCoNiRu‐HESAC has experimental overpotentials of 0.41 and 0.37 V_RHE_ with Tafel slopes of 101 and 210 mVdec^−1^ at a current density of 1 mA cm^−2^ and kinetic current densities of 8.2 and 5.3 mA cm^−2^, respectively, in acidic and alkaline electrolytes, comparable with Pt/C. The synthesized sample was used for Zinc–air battery application with an open circuit potential of 1.393 V and power density of 0.16 W cm^−2^. Based on these theoretical and experimental results, we demonstrate an ML‐ and DFT‐guided strategy for the rational design of HESAC toward enhanced electrochemical ORR and beyond.

## 4 Experimental Section

### Chemicals

Ethanol (96%), KMnO_4_, KOH (>85%), H_2_SO_4_ (95%) Nafion (5 wt.% in a mixture of propanol and water), acrylamide (AM, 99%), H_2_O_2_ (30 wt.%), FeCl_3_•6H_2_O (97%), CoCl_2_•6H_2_O (99%), NiCl_2_•6H_2_O (98%), and RuCl_3_•xH_2_O (99.9%) were purchased from Sigma Inc. USA. Graphite (grafguard, particle size = 350 mm) was utilized for the synthesis of Graphene Oxide (GO) and ultrapure deionized water (DI, 18.2 MΩ) was used for washing and synthesis purposes. All the above‐mentioned chemicals were utilized as received.

### Synthesis of Graphene Oxide (GO)

The modified hummers method involving exfoliation and oxidation of expanded graphite sheets was used to synthesize GO through thermal treatment.^[^ ^32^ ^]^ In this process, 1 g of microwave‐expanded graphite was dispersed in a 500 mL round bottom flask containing 30 mL of H_2_SO_4_ (98%). After 2 h of stirring in an ice bath, 5 g of KMnO_4_ was gradually added to the suspension. The suspension was then stirred for 4 h at room temperature until the color turned pale brownish. Subsequently, 50 mL of deionized water (DI) was slowly added to dilute the solution as the color transitioned to a darker brown. The process was continued by adding 200 mL of DI water and stirring at room temperature for an additional 2 h. After that, to reduce the residual KMnO_4_, H_2_O_2_ (30 wt.%) was added dropwise to the solution, resulting in a bright green color. The solution was stirred for an additional 2 h and then allowed to settle for 24 h. Then, the prepared GO was centrifuged 9 times at 15000 rpm for 30 min and was washed with DI water. Finally, the concentration of the GO solution was reached to 8 mg mL^−1^ using DI water.

### Synthesis of FeCoNiRu‐HESAC

In order to synthesize FeCoNiRu‐HESAC, FeCl_3_•6H_2_O, CoCl_2_•6H_2_O, NiCl_2_•6H_2_O, and RuCl_3_•xH_2_O salts were dissolved as the metal precursors to make 0.05 m solution of Fe^3+^, Co^2+^, Ni^2+^, and Ru^3+^, respectively.^[^ ^33^, ^34^ ^]^ 100 mL of DI water, 1.2 mL of acrylamides (25 wt.%, as a nitrogen precursor), and 125 µL of Fe^3+^, Co^2+^, Ni^2+^, and Ru^3+^ solutions were added into 12.5 ml of 8 mg mL^−1^ GO and kept stirring for 48 h before being freeze–dried for four days. A two‐step pyrolysis process was used as the second annealing step enhances the dispersity of the metal atoms on the nitrogen‐doped graphene support that can produce highly dispersed HESAC by lowering the energy barrier for the formation of single atoms from their corresponding metal compounds.^[^ ^10^, ^35^, ^36^ ^]^ The freeze–dried sample with a brownish color underwent the initial annealing process at a temperature of 500 °C in a one‐inch quartz tube furnace for a duration of 3 h under an Ar atmosphere with a flow rate of 200 sccm. Then the resulting blackish sample was washed five times using ethanol (96%) and H_2_SO_4_ (0.05 m) before undergoing a subsequent freeze–drying step. After that, the freeze–dried sample underwent a second annealing step at a temperature of 600 °C for 3 h under Ar atmosphere with a flow rate of 100 sccm to obtain FeCoNiRu‐HESAC. For the synthesis of the control sample (nitrogen‐doped), no metal precursor was added during the preparation process

### Characterization and Analysis

Scanning transmission electron microscopy (STEM, JEOL JEM‐2100F), scanning electron microscopy (SEM, JEOL JSM‐7800F), and transmission electron microscopy (TEM, JEOL JEM 100CXII) were utilized to examine the structure and morphology of synthesized FeCoNiRu‐HESAC. Elemental mapping was performed using energy dispersive x‐ray analysis (EDX, JEOL JEM 100CXII, and JEOL JSM‐7800F). Elemental composition, elemental bonding states, and chemical structure of the prepared catalyst were verified using X‐ray photoelectron spectroscopy (XPS) with a PHI 5000 VersaProbe III (ULVAC‐PHI). To examine the local environment around the Fe, Co, Ni, and Ru metal atoms, the Fe‐K edge, Co‐K edge, Ni‐K edge, and Ru‐K edge X‐ray Absorption Fine Structure (XAFS) spectra were acquired through the synchrotron radiation‐based Wide‐XAFS facility, BL10C, at Pohang Accelerator Laboratory (PAL). Raman spectroscopy (Renishaw Raman RM3000 scopes with a 514 nm laser source) and X‐ray crystallography (XRD, PANalytical) were used to investigate the ratio of the G band to the D band, the interlayer spacing, and the crystallinity of the synthesized catalyst. The ORR performances and the Zinc–air battery polarization curves were acquired using a rotating ring disk electrode device (RRDE‐3A, ALS Co.) along with a Gamry 5000E workstation.

### Electrocatalytic and Zinc–Air Battery Performances

In order to perform the ORR tests, 2.5 mg of each nitrogen‐doped, FeCoNiRu‐HESAC, or Pt/C samples, 50 µL of Nafion, 150 mL ethyl alcohol, and 350 µL DI water were mixed and sonicated for 10 h resulting in a homogeneous black ink. After that, 3 droplets of 10 µL of the black ink were dropped on the surface of the neat and clean, glassy carbon electrode (GCE) with a surface of 0.196 cm^2^, and a diameter of 0.5 cm, leading to the loading of 0.695 mg cm^−2^. As in Figure S33 (Supporting Information), a three‐electrode cell loaded with either 0.5 m H_2_SO_4_ or 0.1 m KOH aqueous electrolytes for the ORR performance measurements was used. The GCE loaded with catalysts was used as a working electrode, an Ag/AgCl electrode was used as a reference electrode, and a Pt wire was used as a counter electrode. The potentials relative to reversible hydrogen electrode (RHE) were then obtained from: E_RHE_ = E_Ag/AgCl_ + 0.197 + 0.0592 × pH (for example, E_RHE_ = E_Ag/AgCl_ + 0.2562 for 0.5 m H_2_SO_4_ with the pH of 1 and E_RHE_ = E_Ag/AgCl_ + 0.9666 for 0.1 M KOH with the pH of 13).

Highly pure Ar (99.99%) and O_2_ (99.99%) were purged into KOH and H_2_SO_4_ electrolytes for 30 min before conducting the ORR performances. The cyclic voltammetry (CV) tests were performed with a scan rate of 50 mVs^−1^ and step size of 5 mV, while the linear sweep voltammetry (LSV) tests were carried out from positive to negative potentials with a scan rate of 10 mVs^−1^, step size of 5 mV, and different rotating speeds of 400, 800, 1200, 1600, and 2000 rpm. The ORR Tafel slopes were determined from the Tafel plots (Log(current density) versus working potential), which were obtained from corresponding LSV polarization curves at a rotating speed of 2000 rpm. Koutecky–Levich (K–L) plot was obtained according to the following equation:^[^ ^34^ ^]^

(11) $$\frac{1}{j}=\frac{1}{B\omega^{1/2}}\;+\frac{1}{j_{K}}$$

(12) $$B\;=\;0.62nFC_{0}D_{0}^{2/3}v^{-1/6}\;\mathrm{and}\;\;j_{K}=\;nFkC_{0}$$

where *j*, *j_K_*, and ω are the measured current density, the kinetic current density, and the electrode rotation rate, respectively. *D_0_*, *C_0_*, *F*, *v*, and k are the diffusion coefficient of O_2_, the bulk concentration of O_2_, the Faraday constant, the kinematic viscosity of the electrolyte, and the electron‐transfer rate constant, respectively.

Zinc–air battery tests were conducted by vigorously mixing 60 mg FeCoNiRu‐HESAC, 375 µL Nafion, 563 mL ethyl alcohol, and 563 µL DI water for 10 min to obtain a homogeneous black ink. Subsequently, the 250 µm thick ink was coated on a carbon cloth that was dried out overnight to obtain a loading of 0.95 mg cm^−2^. After that, the catalyst‐coated carbon cloth was utilized as the cathode and a 2 mm thick zinc plate was utilized as the anode. The Zinc–air cell was then filled with 6 m KOH electrolyte, and the measurement of polarization curves was performed at room temperature on a Gamry 5000E workstation to obtain voltage and power density versus current.

### DFT Calculations

Density functional theory (DFT) was implemented in the Vienna ab initio simulation package (VASP 5.4.4) to calculate the atomic and electronic structures of the catalyst. Spin‐polarized DFT calculations with the Perdew–Burke–Emzerhof (PBE) functional and a plane–wave basis set with the cutoff energy of 500 eV were used.^[^ ^13^, ^37^ ^]^ DFT‐D3 method to consider van der Waals (London dispersion) interactions was used.^[^ ^38^ ^]^ VASPsol was used to include implicit solvation, with the default dielectric constant of *ε* = 78.4. For structure relaxation, the Brillouin zone was sampled using a Monkhorst–Pack scheme with a 4 × 4 × 1 k‐point grid. As shown in Figure 1 and Figure S2 (Supporting Information), 4 × 4, 5 × 5, and 6 × 6 supercells of the graphene with the dimensions of 12.28 Å × 12.28 Å, 14.74 Å × 14.74 Å, and 17.19 Å × 17.19 Å were constructed, and Fe, Co, Ni, and Ru metals were placed at carbon vacancies and coordinated with nitrogen atoms in either a planar structure^[^ ^5^ ^]^ or an inversed sandwich with hydroxyl bridges^[^ ^39^, ^40^ ^]^ structure. A vacuum space of 20 Å in the *z* direction was applied to lower periodic effects between mirror images.^[^ ^41^, ^42^ ^]^ The force convergence criteria on each atom for structure relaxation was set to 0.03 eV Å^−1^. The energy convergence criteria was set to 10^−6^  and 10^−7^ eV, respectively, for structure relaxation and self‐consistent calculations.

The vibrational frequency calculation was performed only for OH*, O*, OOH*, OO*, and H* intermediates to obtain phonon frequencies and hence the zero‐point energy (Δ*E*_ZPE_), entropy, and specific heat contributions to calculate the Gibbs free energies at *T* = 298.15 K.^[^ ^43^ ^]^ Spin and charge transfer densities were visualized with VESTA program while density‐of‐states (DOS) and spin density were generated using the VASPKIT1.3.0 program.^[^ ^44^ ^]^ The relaxed structures generated from the VASPsol code were used to obtain the XANES spectra using Finite Difference Method Near Edge Structure (FDMNES) code.^[^ ^45^ ^]^

### Statistical Analysis

The data was pre‐processed using Excel software and Python. The Origin software and Python were used to analyze and plot the data. Electrochemical ORR test data were adjusted with reference to reversible hydrogen electrode (RHE). Experiments were conducted multiple times to ensure accurate and reliable results.

## Conflict of Interest

The authors declare no conflict of interest.

## Supporting information

Supporting Information

## Data Availability

The data that support the findings of this study are available in the supplementary material of this article.
