# Supplementary material for: A High‐Entropy Single‐Atom Catalyst Toward Oxygen Reduction Reaction in Acidic and Alkaline Conditions
Source: Adv Sci (Weinh). 2024 Apr 30;11(26):2309883. doi: 10.1002/advs.202309883 (PMC11234427; doi:10.1002/advs.202309883)
Supplement: Supplementary file 1 — Supporting Information [file ADVS-11-2309883-s001.docx]

**Supporting Information**

**A High-entropy Single Atom Catalyst towards Oxygen Reduction Reaction in Acidic and Alkaline Conditions**

Mohsen Tamtaji,^1^ Min Gyu Kim,^2^ Jun WANG,^3^ Patrick Ryan Galligan,^3^ Haoyu zhu^1^, Faan-Fung Hung,^1^ Zhihang Xu,^4^ Ye Zhu,^4^* Zhengtang Luo,^3^* William A. Goddard III,^5^* GuanHua Chen^1,6^*

*^1^Hong Kong Quantum AI Lab Limited, Pak Shek Kok, Hong Kong SAR, China*

*^2^Beamline Research Division, Pohang Accelerator Laboratory (PAL), Pohang University of Science and Technology, Pohang 37673, Republic of Korea*

*^3^Department of Chemical and Biological Engineering, Guangdong-Hong Kong-Macao Joint Laboratory for Intelligent Micro-Nano Optoelectronic Technology, William Mong Institute of Nano Science and Technology, and Hong Kong Branch of Chinese National Engineering Research Center for Tissue Restoration and Reconstruction, The Hong Kong University of Science and Technology, Clear Water Bay, Kowloon, Hong Kong, 999077, P.R. China*

*^4^Department of Applied Physics, Research Institute for Smart Energy, The Hong Kong Polytechnic University, Hong Kong, China*

*^5^Materials and Process Simulation Center (MSC), MC 139-74, California Institute of Technology, Pasadena CA, 91125, USA*

*^6^Department of Chemistry, The University of Hong Kong, Pokfulam Road, Hong Kong SAR, China*

*Corresponding Authors, email: [ye.ap.zhu@polyu.edu.hk](mailto:ye.ap.zhu@polyu.edu.hk), [keztluo@ust.hk](mailto:keztluo@ust.hk), [ghc@everest.hku.hk](mailto:ghc@everest.hku.hk), and [wag@caltech.edu](mailto:wag@caltech.edu)

**S1. DFT calculations**

The number of combinations (*M*) of *n* transition metals into the HESAC with *m* (<*n*) active sites can be calculated as follows:

| $M=\frac{n!}{m!\times\left( n-m \right)!}$ | (S1) |
| --- | --- |

It indicates that by increasing the number of active sites (*m*) in the HESAC catalyst, the number of combinations (*M*) increases exponentially. For instance, considering 3d, 4d, and 5d transition metals (*n*=30), HESAC with four active sites (*m*=4) leads to 27405 combinations, 913 times more than 30 combinations for single atom catalysts (SAC).

**Table S1.** The number of combinations (*M*) of *n* transition metals into the HESAC with *m* active sites.

| ***m*** | ***M*_HESAC_** | ***M*_HESAC_/*M*_SAC_** | **ln(*M*_HESAC_)** |
| --- | --- | --- | --- |
| **1***^a^* | 30 | 1 | 3.4 |
| **2***^b^* | 435 | 15 | 6.1 |
| **3***^c^* | 4060 | 135 | 8.3 |
| **4***^d*^* | 27405 | 913 | 10.2 |
| **5** | 142506 | 4750 | 11.9 |
| **6** | 593775 | 19793 | 13.3 |
| **7** | 2035800 | 67860 | 14.5 |
| **8** | 5852925 | 195098 | 15.6 |
| **9** | 14307150 | 476905 | 16.5 |
| **10** | 30045015 | 1001501 | 17.2 |

*a*: This is single atom catalyst (SAC).

*b*: This is double atom catalyst (DAC).

*c*: This is triple atom catalyst (TAC).

*d**: In this work, a HESAC with *m*=4 (Fe, Co, Ni, and Ru) active sites is studied.


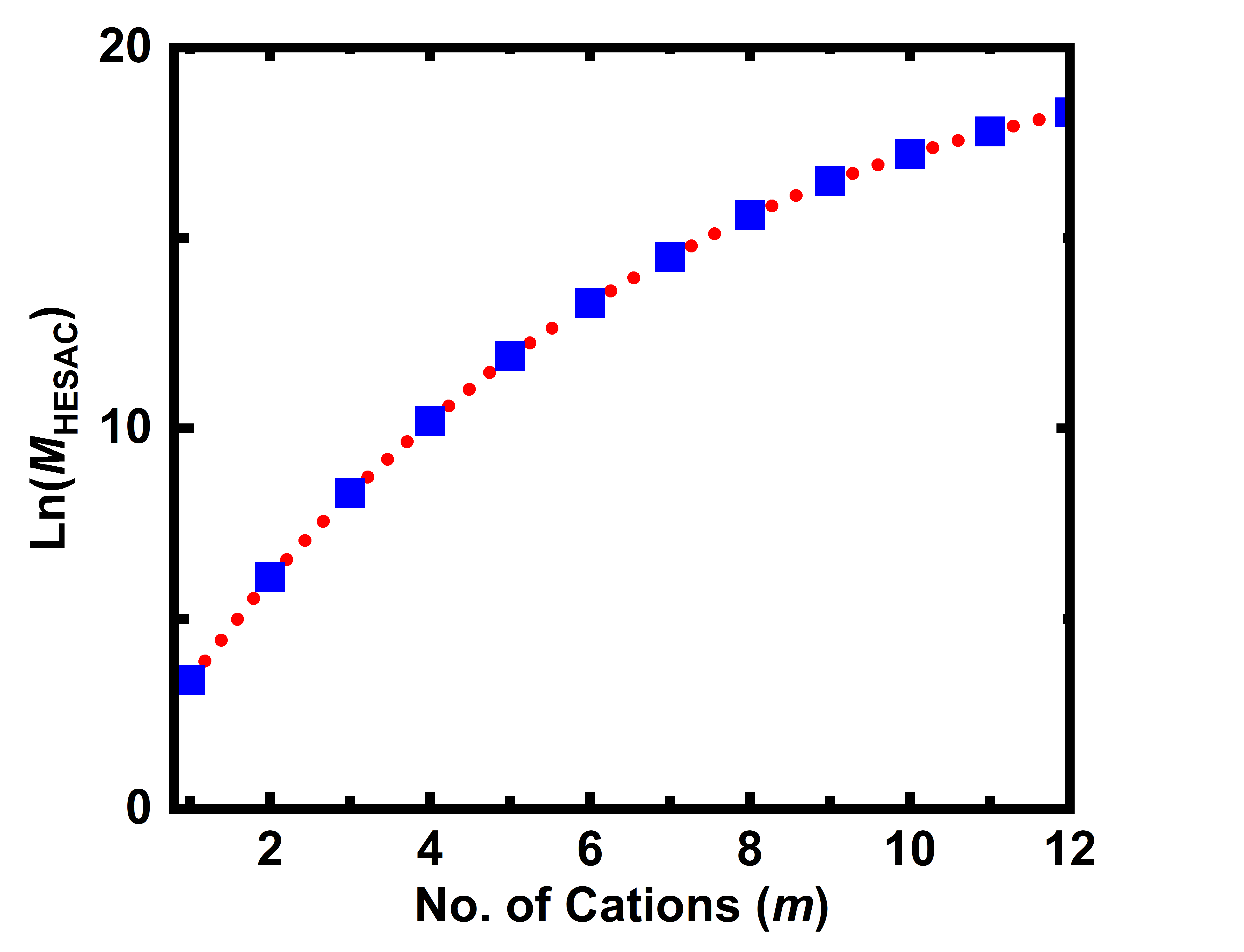


**Figure S1.** The number of combinations (*M*) of *n* transition metals into the HESAC with *m* active sites.


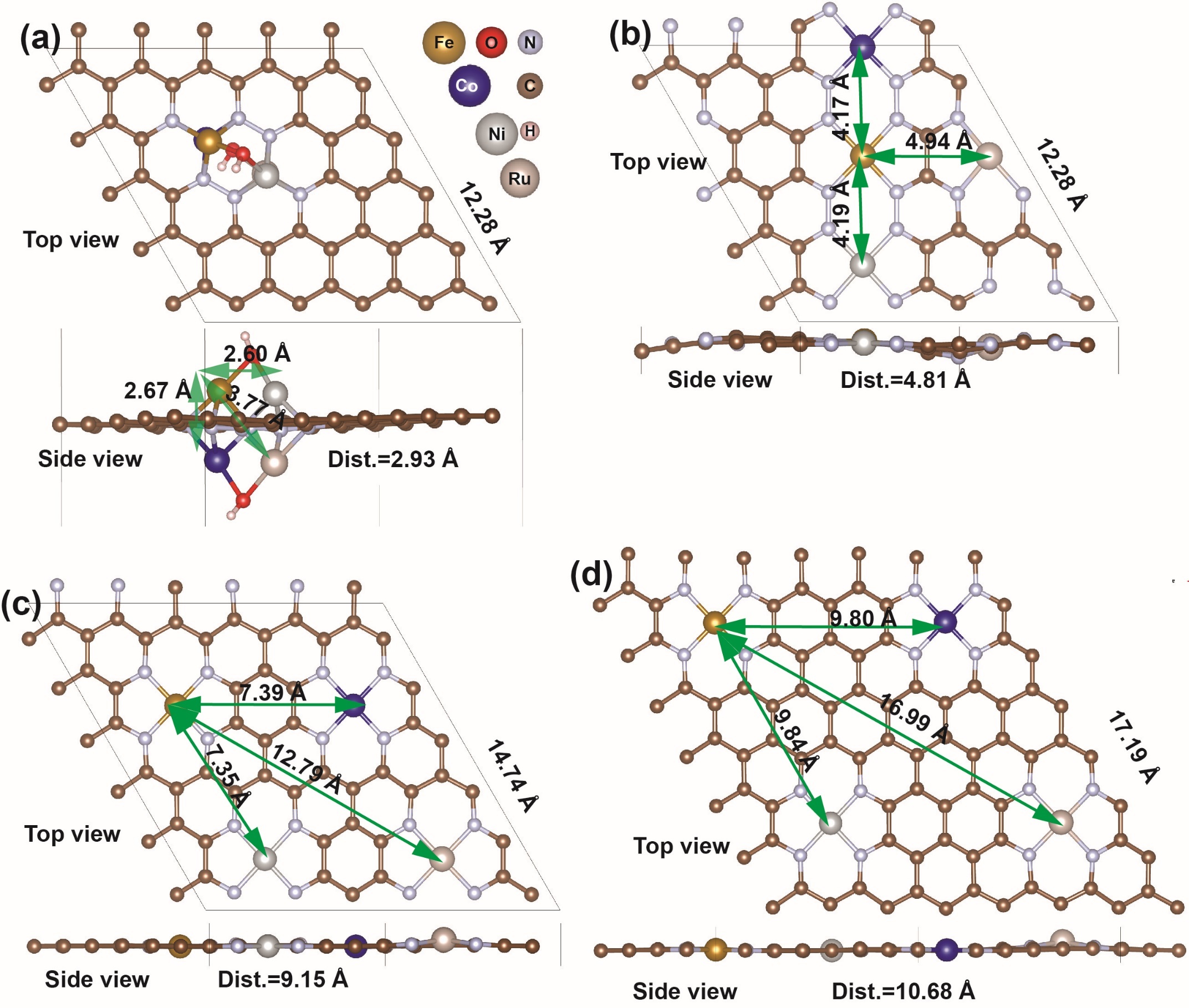


**Figure S2.** Top and side views of the optimized structural models of an inversed sandwich structure and planar FeCoNiRu-HESAC with averaged intermetallic distances (Dist.) of (a) Dist.=2.93 Å, (b) Dist.=4.81 Å, (c) Dist.=9.15 Å, and (d) Dist.=10.68 Å. Distances are in Å. In the inversed sandwich structure, Fe, Co, Ni, and Ru are out of plane while in planar structures Fe, Co, Ni, and Ru are anchored on the moiety side, in plane.

**Table S2.** Computed enthalpy, entropy, and Gibbs free energy of mixing for FeCoNiRu-HESAC at 600 K for various intermetallic distances (Dist.).

| **Energy** | **Dist.= 2.93Å** | **Dist.= 4.81Å** | **Dist.= 6.11Å*^a^*** | **Dist.= 9.15Å** | **Dist.= 10.68Å** | **Dist.=15Å** |
| --- | --- | --- | --- | --- | --- | --- |
| **∆H**_mix_ **(eV)** | -1.1 | -1.19 | -1.27 | -1.74 | -1.86 | -2.14 |
| **T∆S**_mix_ **(eV)** | 0.10 | 0.11 | 0.11 | 0.09 | 0.08 | 0.02 |
| **∆G**_mix_ **(eV)** | -1.2 | -1.3 | -1.38 | -1.83 | -1.94 | -2.162 |

*a*: This is the structure in which Fe active site leads to an 0.46 V overpotential.


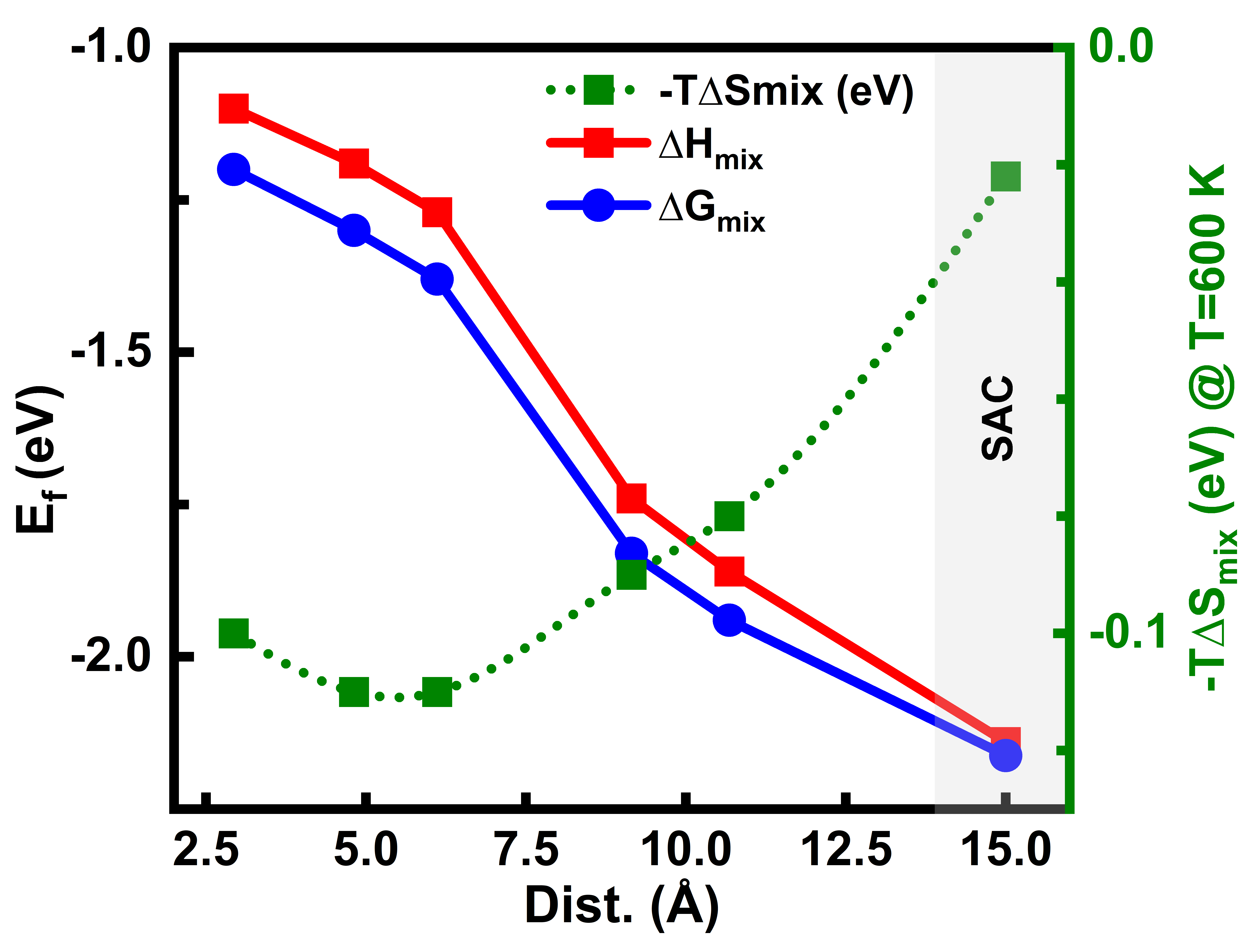


**Figure S3.** Computed enthalpy, entropy, and Gibbs free energy of mixing for FeCoNiRu-HESAC at 600 K for various intermetallic distances (Dist.).

**Table S3.** Computed dissolution potential (U_diss_, V) of Fe, Co, Ni, and Ru metals from FeCoNiRu-HESAC with intermetallic distances (Dist.) of 6.11 Å, where E_i_^bulk^ is the total energy of i^th^ metal atom in their bulk phase (eV/atom), *n* is the number of electrons transferred during the dissolution,^[1]^ and U^o^_diss_ (V)^[2]^ is the standard dissolution potential of the metals.

| **Metal** | **E_i_^bulk^ (eV/atom), Ref.** ^[3]^ | **U^o^_diss_ (V), Ref.** ^[1]^ | ***n, Ref.*** ^[2]^ | **U_diss_ (V)** |
| --- | --- | --- | --- | --- |
| **Fe** | -8.45 | -0.45 | 2 | 0.24 |
| **Co** | -7.10 | -0.28 | 2 | 0.41 |
| **Ni** | -5.55 | -0.26 | 2 | 0.43 |
| **Ru** | -9.20 | 0.46 | 2 | 1.15 |


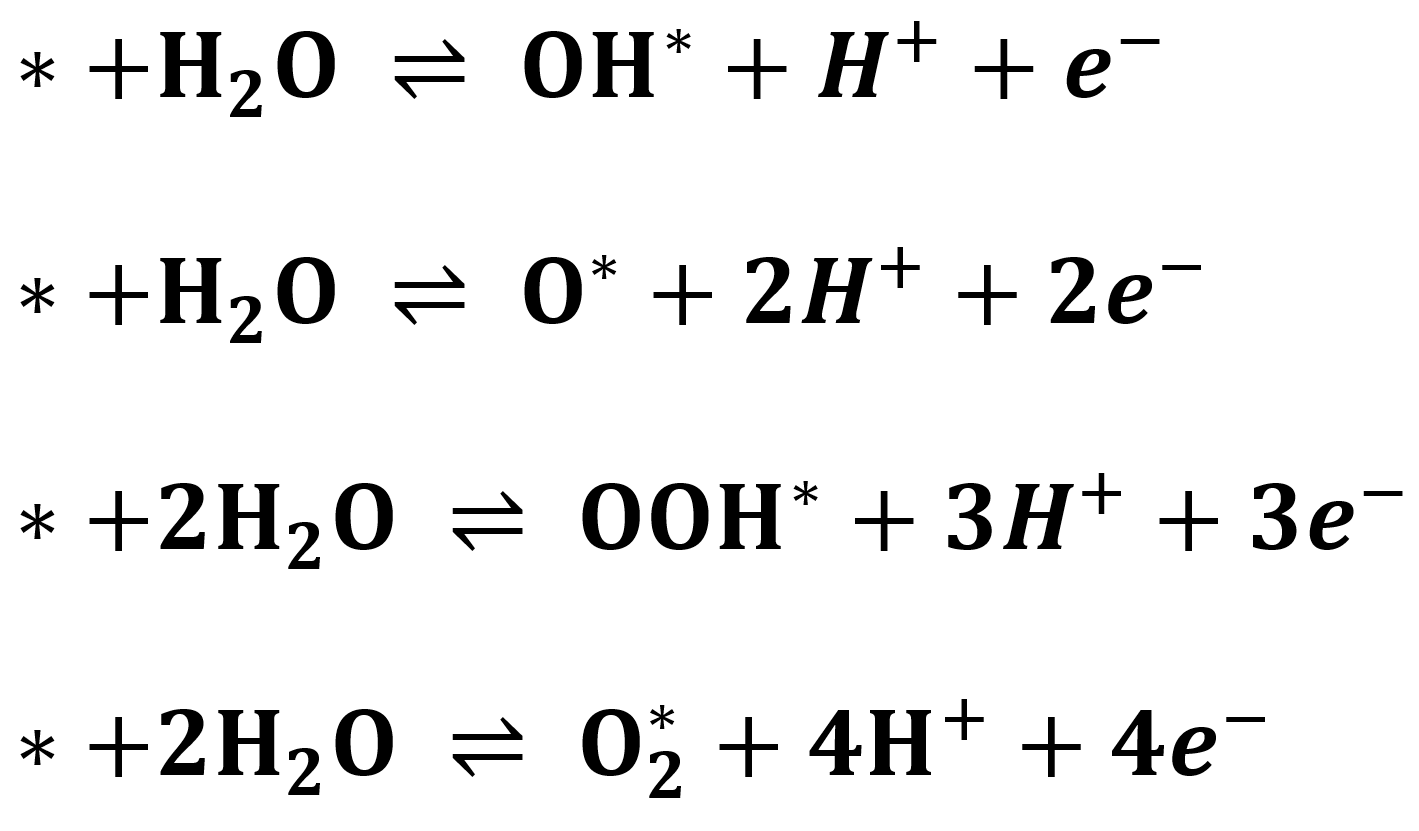


**Schematic S1.** The fundamental steps for ORR electrocatalysis on the metal active sites of HESAC.

**Table S4.** Values used for the entropy (TS) and zero-point energy (ZPE) corrections in determining the free energy of reactants, products, and intermediate species adsorbed on high entropy single atom catalyst (HESACs).

| **Species** | **T×S (eV) (298.15 K)** | **ZPE (eV)** | **ZPE-TS (eV)** |
| --- | --- | --- | --- |
| **H*** | 0.00 | 0.17 | 0.24 |
| **O*** | 0.00 | 0.07 | -0.06 |
| **OH*** | 0.00 | 0.33 | 0.27 |
| **OOH*** | 0.00 | 0.43 | 0.24 |
| **OO*** | 0.00 | 0.14 | -0.12 |
| **H_2_(g)** | 0.41 | 0.27 | --- |
| **H_2_O(g)** | 0.58 | 0.57 | --- |

**Table S5.** Scaling relationship limit coefficients based on **Figure 2a**.

| **Intermediate** | **Ideal coefficients*^a^*** | **Real coefficients** |
| --- | --- | --- |
| **OH*** | $a_{0}=1 \mathrm{and}\beta_{0}=0$ | $a_{0}=1 \mathrm{and}\beta_{0}=0 eV$ |
| **O*** | $a_{1}=2 \mathrm{and}\beta_{1}=0$ | $a_{1}=1.532 \mathrm{and}\beta_{1}=0.650 eV$ |
| **OOH*** | $a_{2}=3 \mathrm{and}\beta_{2}=0$ | $a_{2}=0.904 \mathrm{and}\beta_{2}=3.301 eV$ |
| **OO*** | $a_{3}=4 \mathrm{and}\beta_{3}=0$ | $a_{3}=0.798 \mathrm{and}\beta_{3}=3.751 eV$ |
| **H*** | _---_ | $a_{4}=0.763 \mathrm{and}\beta_{4}=-0.235 eV$ |

*^a^*: Without considering scaling relationship limit, the ideal OER and ORR overpotentials is 0 V.

Scaling relationships are as follows:

| ${\Delta G}_{O}=a_{1}{\Delta G}_{OH}+\beta_{1}$ | (S4) |
| --- | --- |
| ${\Delta G}_{OOH}=a_{2}{\Delta G}_{OH}+\beta_{2}$ | (S5) |
| ${\Delta G}_{OO}=a_{3}{\Delta G}_{OH}+\beta_{3}$ | (S6) |
| ${\Delta G}_{H}=a_{4}{\Delta G}_{OH}+\beta_{4}$ | (S7) |


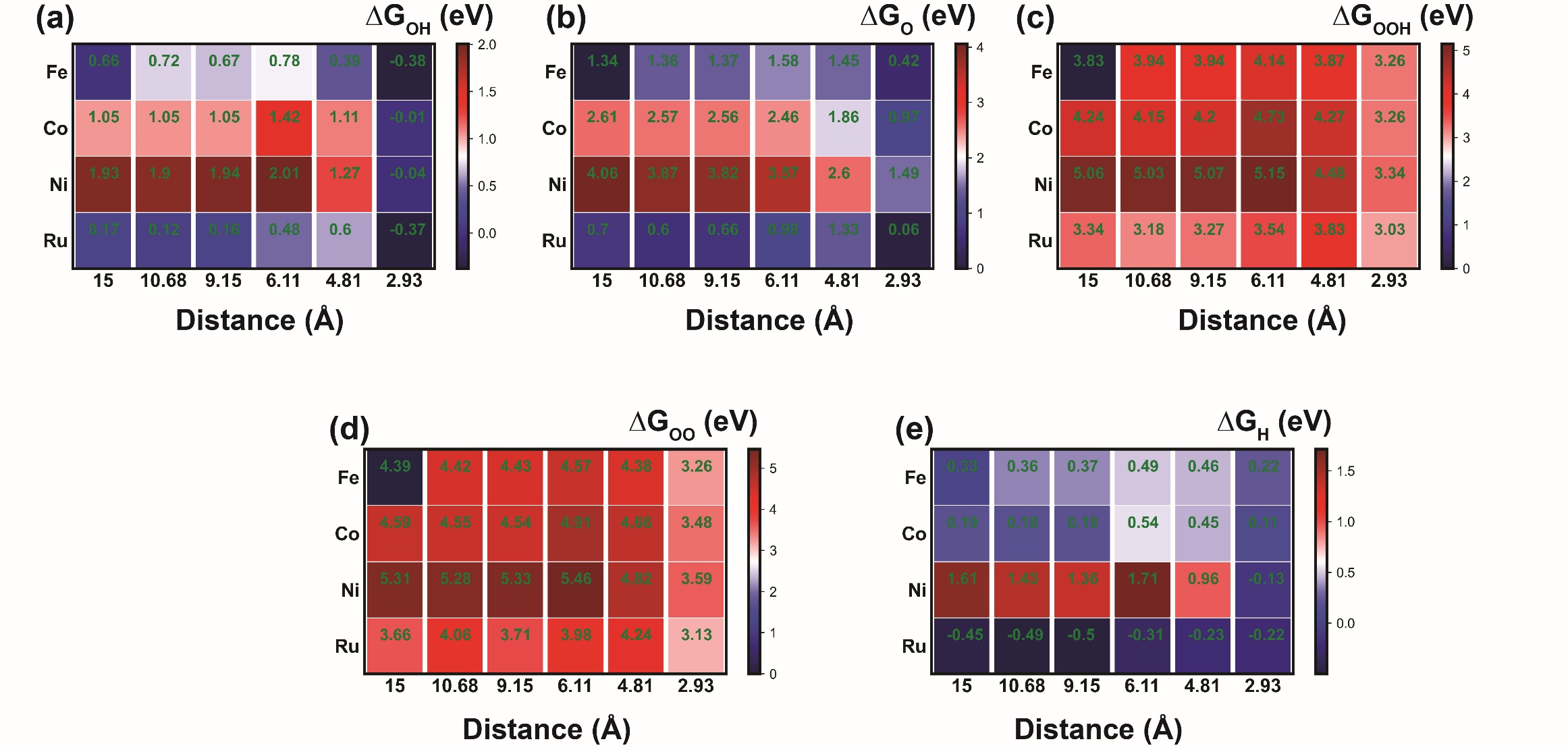


**Figure S4.** Gibbs free energy of H*, OH*, O*, OOH*, and OO* intermediates on Fe, Co, Ni, and Ru active sites in FeCoNiRu-HESAC with the averaged intermetallic distance of 2.93 Å, 4.81 Å, 6.11 Å, 9.15 Å, and 10.68 Å. Dist.=15 Å is considered as the single atom catalyst.


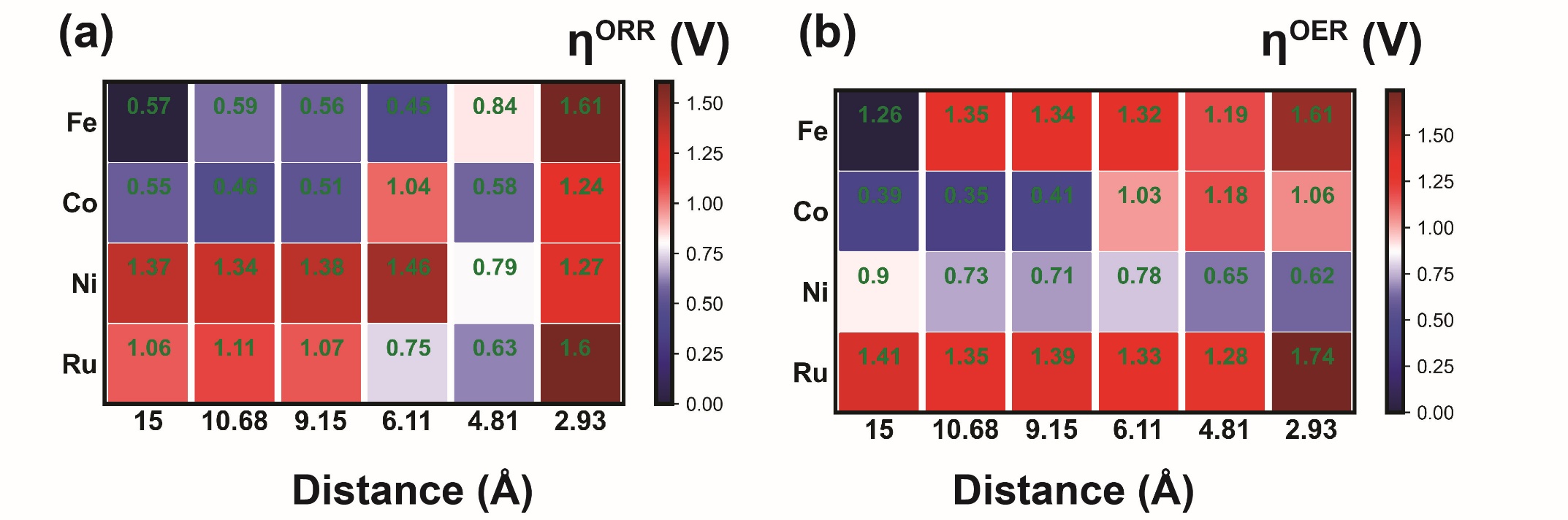


**Figure S5.** ORR and OER overpotentials of Fe, Co, Ni, and Ru active sites in FeCoNiRu-HESAC with the averaged intermetallic distance of 2.93 Å, 4.81 Å, 6.11 Å, 9.15 Å, and 10.68 Å. Dist.=15 Å is considered as the single atom catalyst.


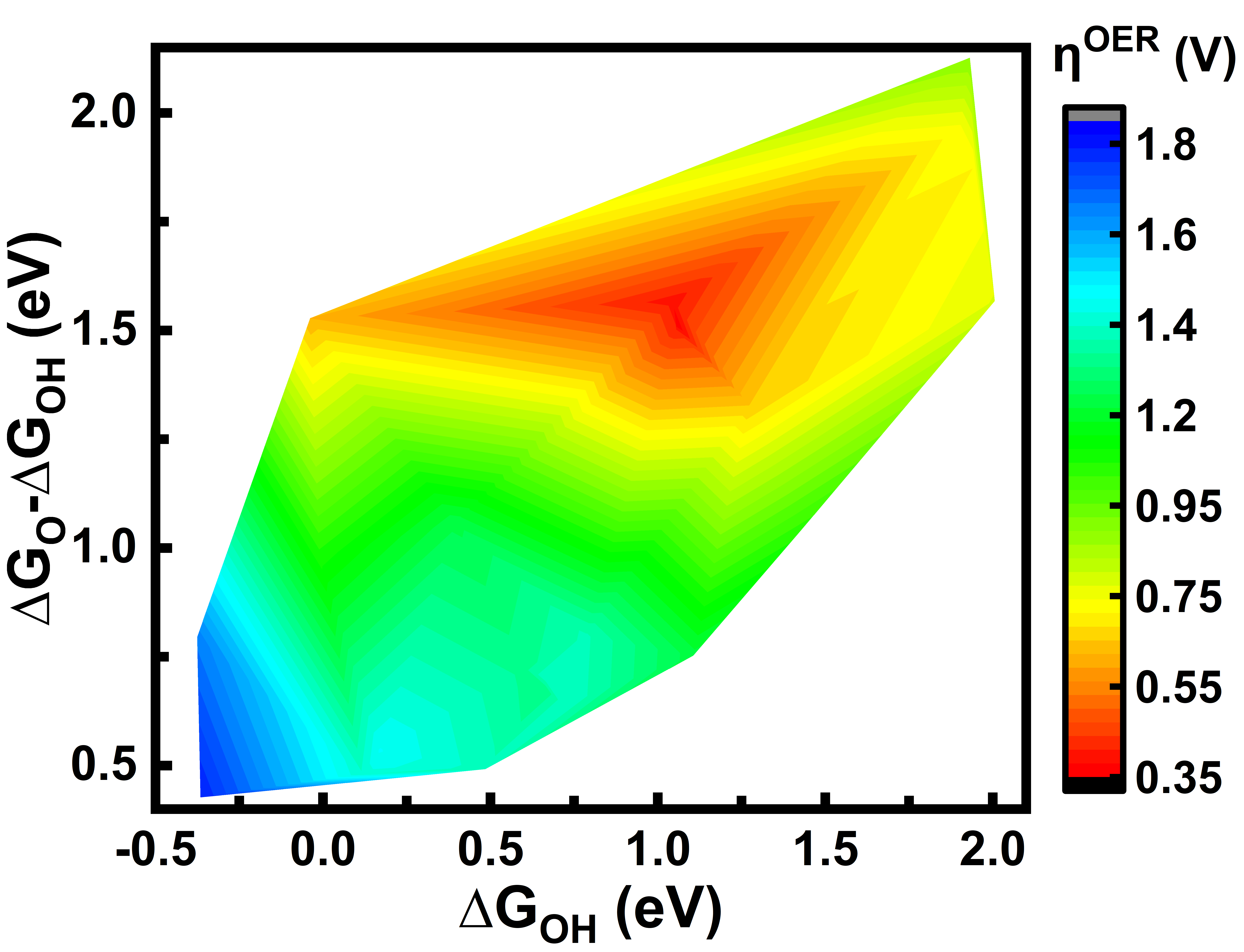


**Figure S6.** Counter plot of ORR overpotential vs. ∆G_OH*_ and ∆G_O*_-∆G_OH*_ and (b) ∆G_OOH*_ and ∆G_O*_-∆G_OH*_.


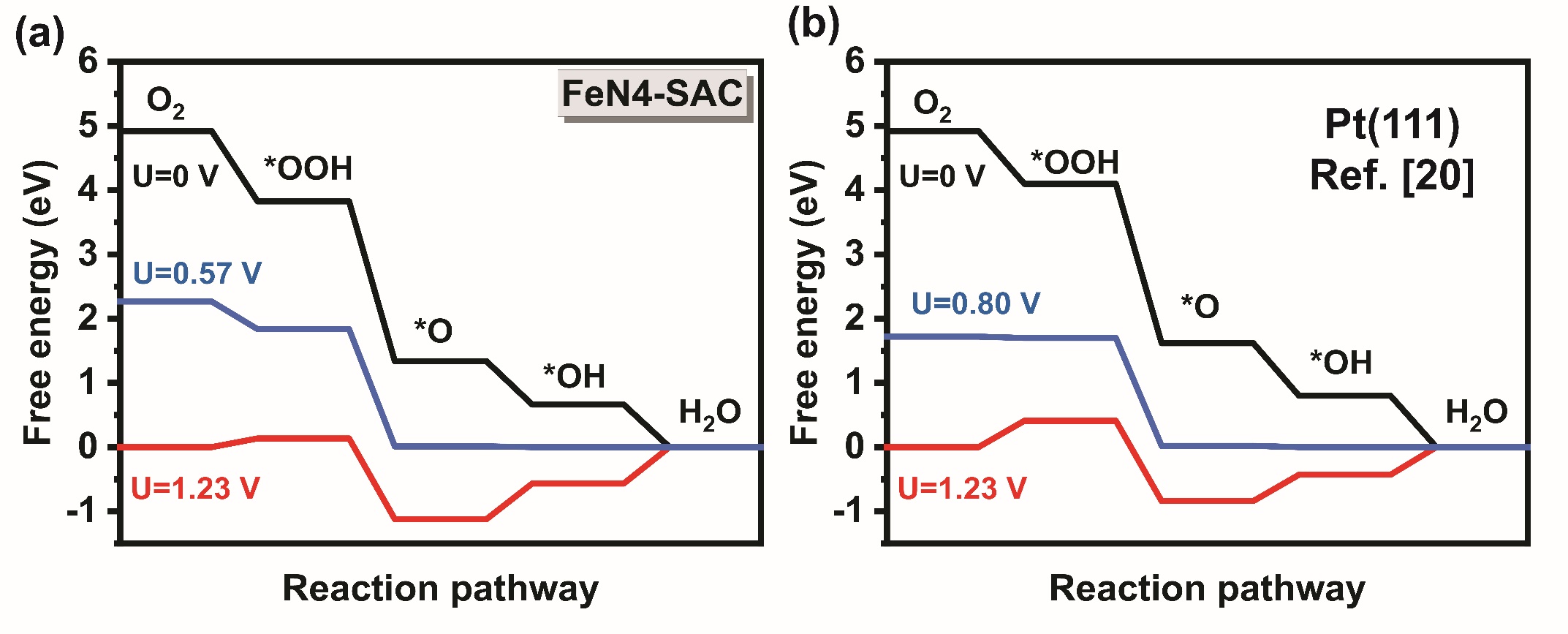


**Ref. [4]**

**Figure S7. Gibbs free energy diagram.** The Gibbs free energy diagram for oxygen reduction reaction (ORR) corresponding to Fe active site in (a) FeN4-SAC and (b) Pt(111), Ref. ^[4]^.


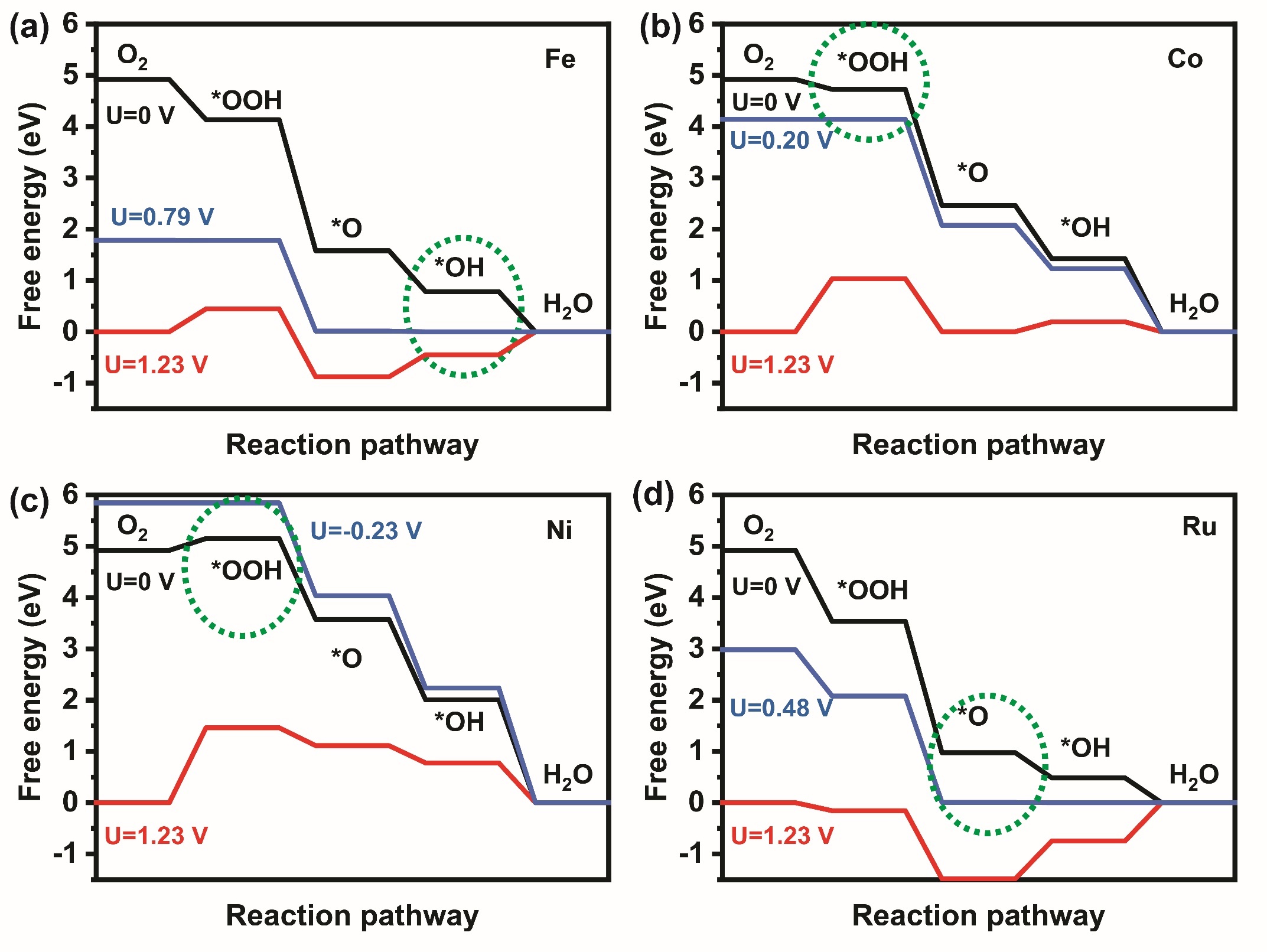


**Figure S8. Gibbs free energy diagram.** The Gibbs free energy diagram for oxygen reduction reaction (ORR) corresponding to (a) Fe, (b) Co, (c) Ni, and (d) Ru active sites in FeCoNiRu-HESAC with intermatallic diatance of 6.1 Å. The dashed green circles show the rate limiting step for each metal. The rate limiting step for Fe, Co, Ni, and Ru sites are the desorption of OH*, proton transfer to OO*, proton transfer to OO*, and proton transfer to O*, respectively.


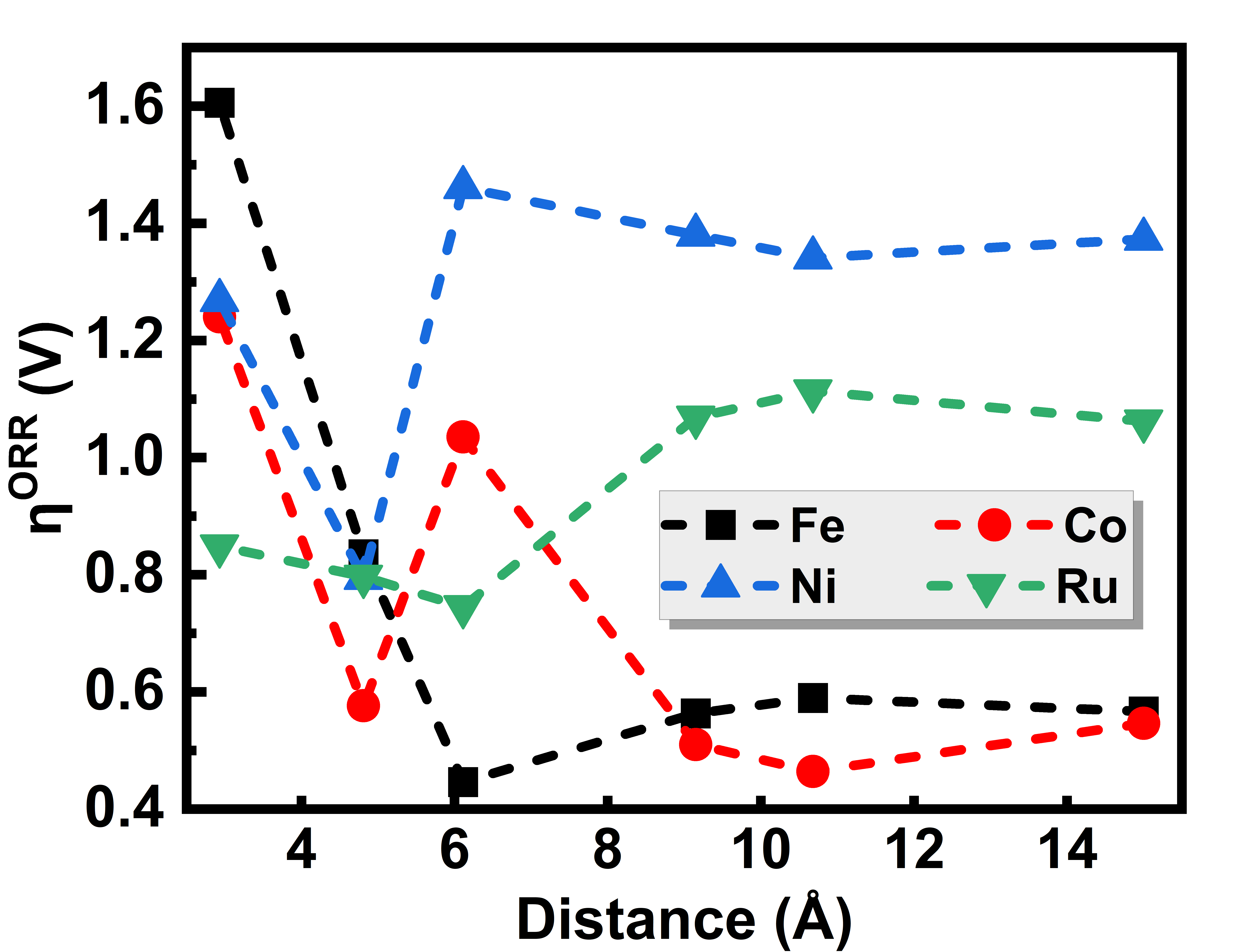


**Figure S9.** ORR overpotentials for Fe, Co, Ni, and Ru active sites in FeCoNiRu-HESAC versus the averaged intermetallic distance (Dist.), indicating that the scaling relationship is weakened below Dist.=6.11 Å.

**
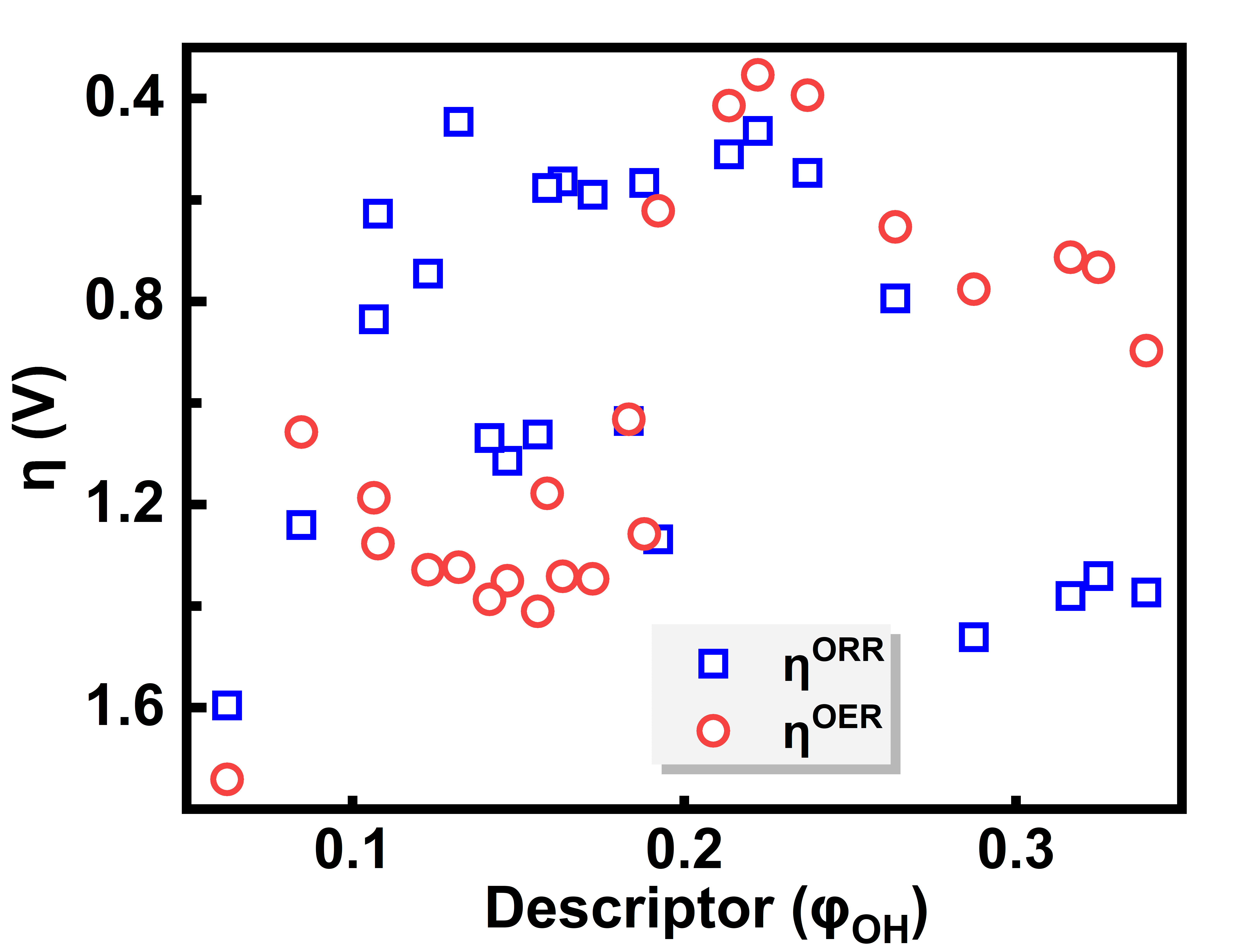
**

**Figure S10.** ORR and OER overpotentials versus new descriptor (φ_OH_).

**
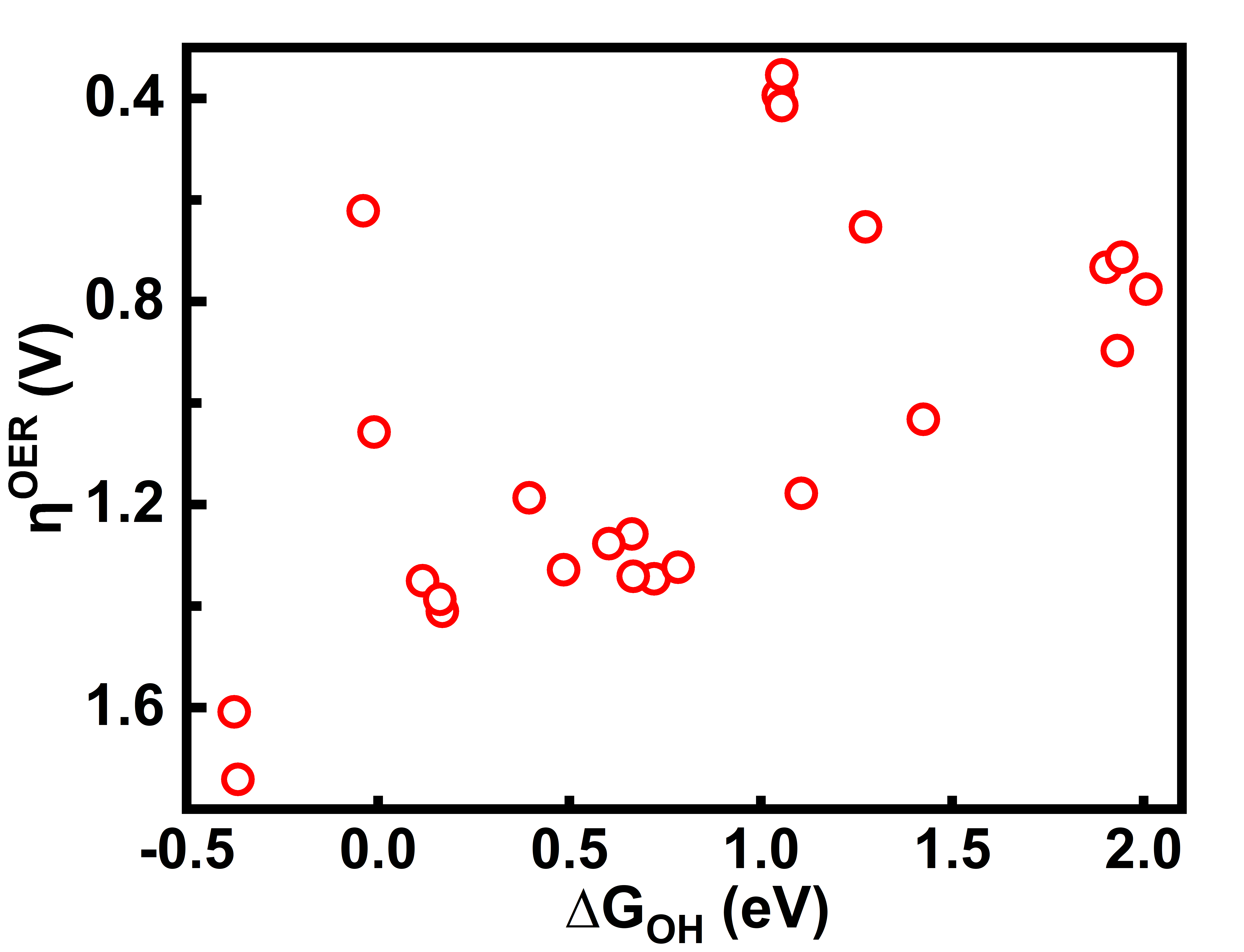
**

**Figure S11.** OER overpotentials versus ∆G_OH_.


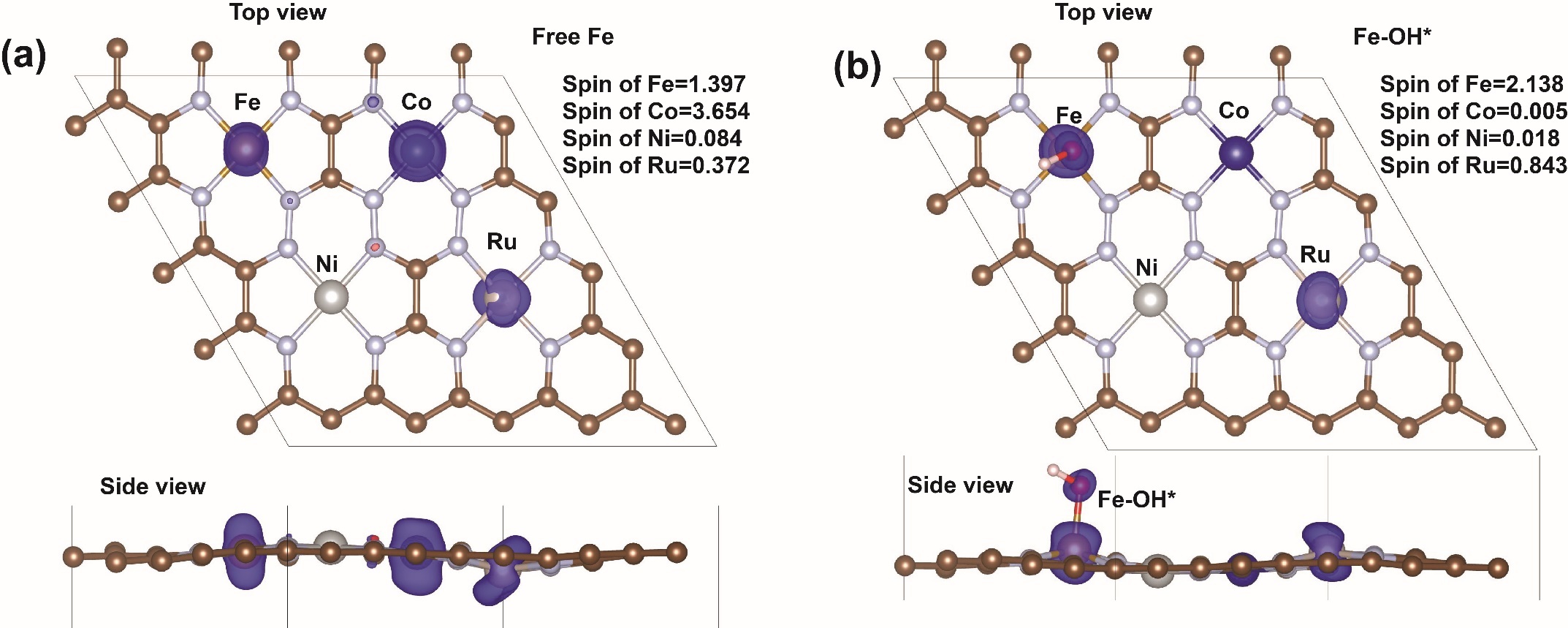


**Figure S12.** Spin density of FeCoNiRu-HESAC with the averaged intermetallic distance of 6.11 Å, indicating the small delocalization of unpaired electrons of high-lying d_z2_ orbital of metal atoms into the ligands. The blue color represents alpha spin while the red color represents beta spin, Isosurface value = 0.02 e/Å^3^.


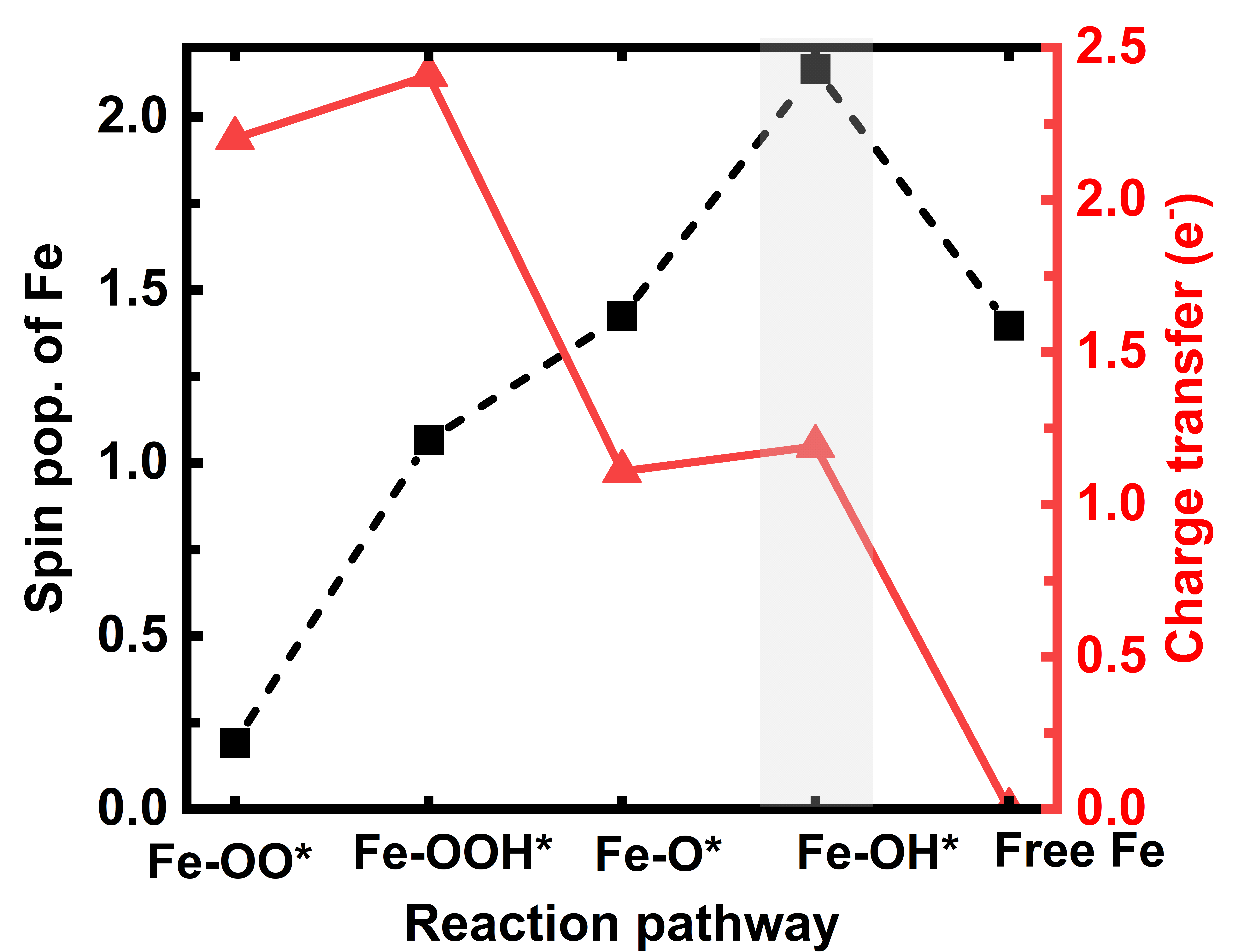

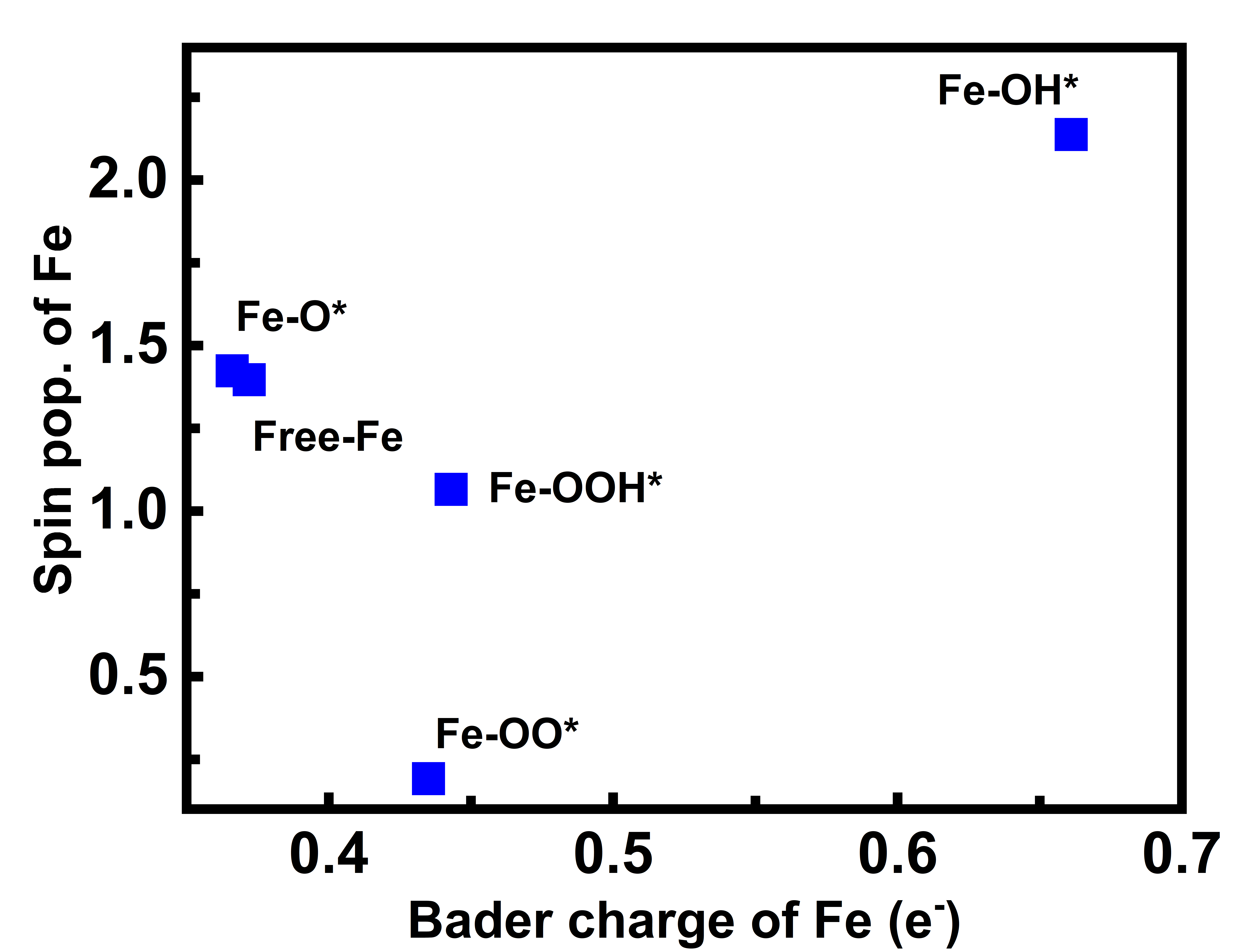


**(b)**

**(a)**

**Figure S13.** (a) The spin population and charge transfer analysis of the Fe site in FeCoNiRu-HESAC with the averaged intermetallic distance (Dist.) of 6.11 Å through ORR pathway, indicating the highest spin density is on Fe-OH*. (b) The spin population versus the charge of Fe site in FeCoNiRu-HESAC with Dist. of 6.11 Å through ORR pathway.


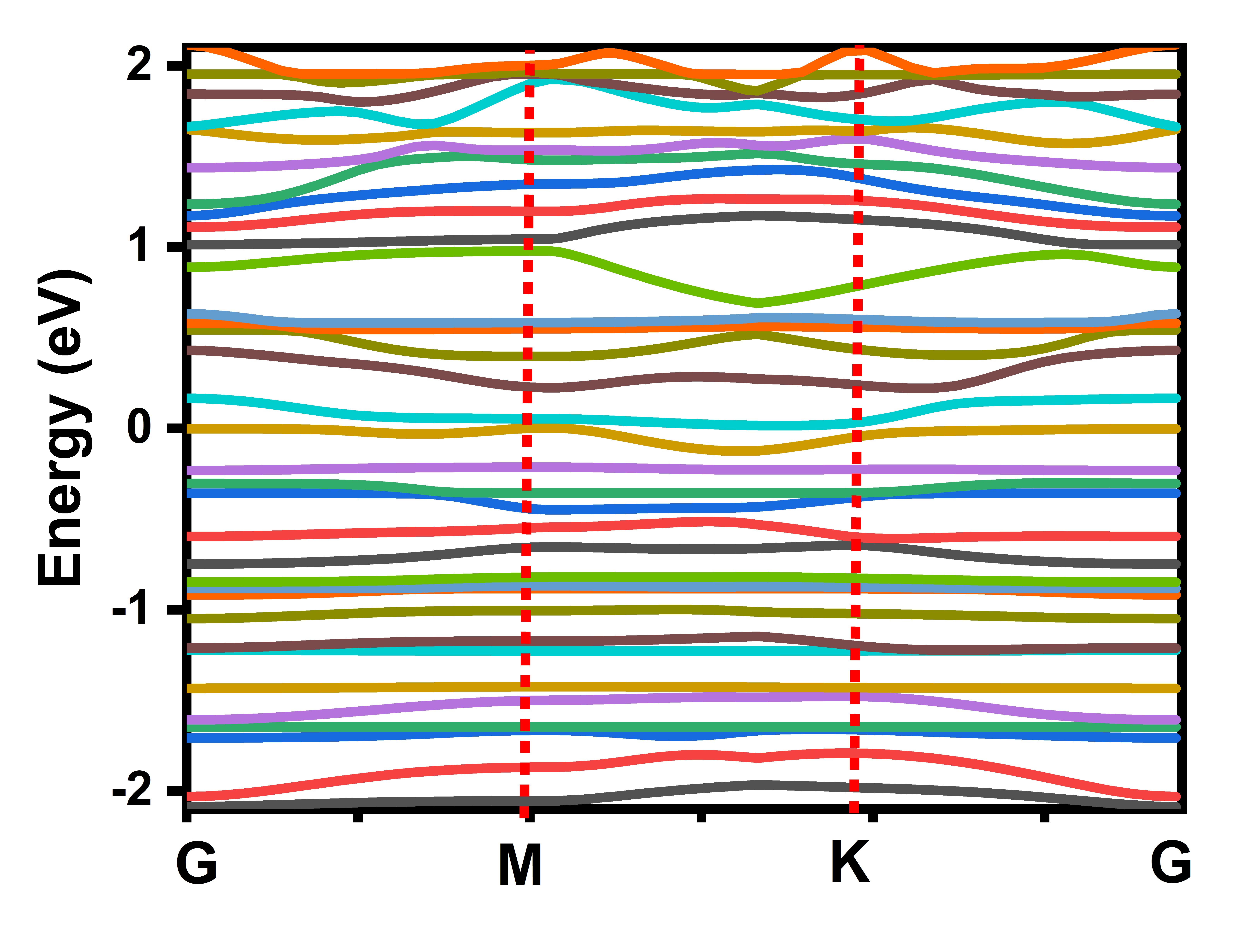

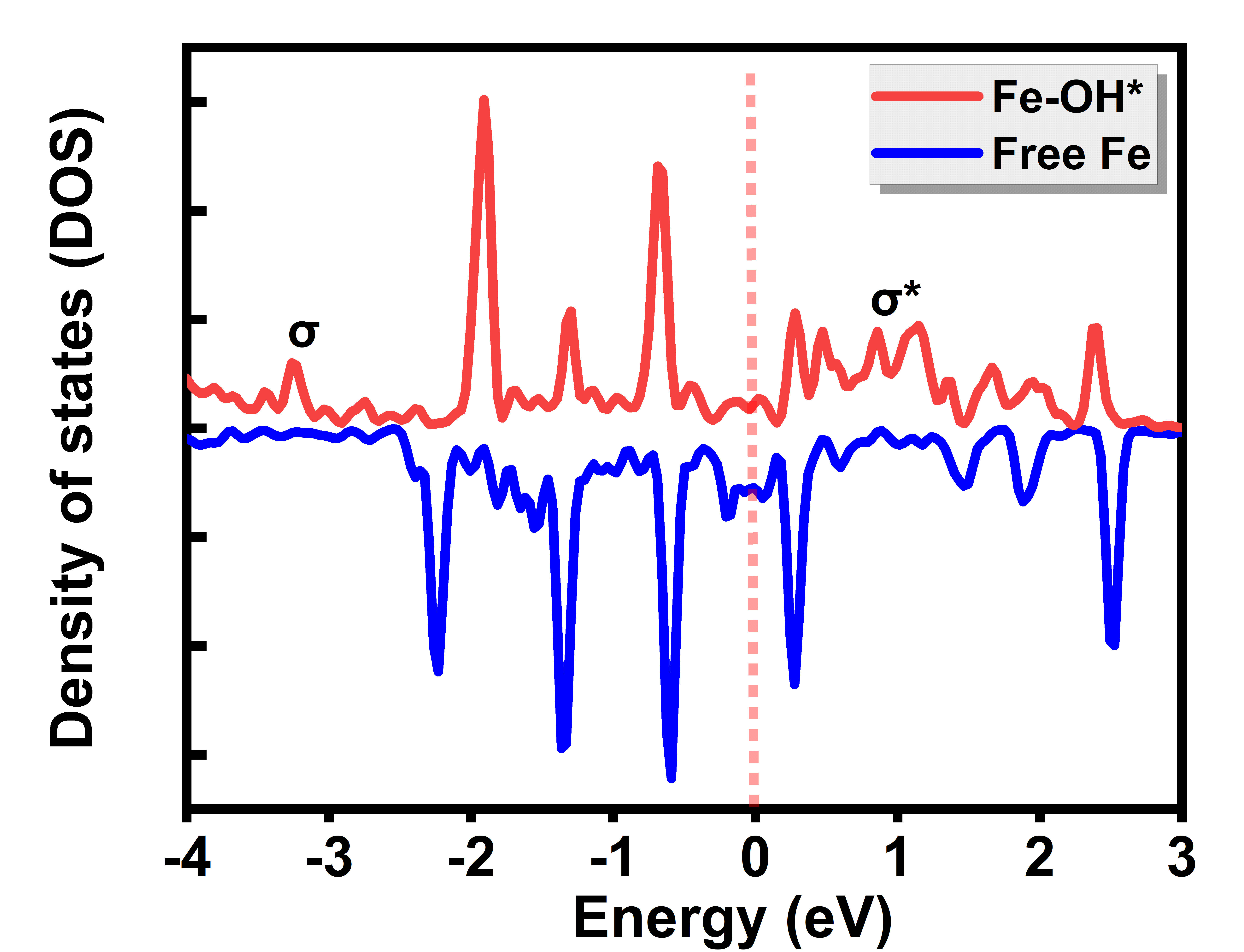


**(b)**

**(a)**

**Figure S14. Band steructure and density of states (DOS).** Bandstructutre of FeCoNiRu-HESAC with the averaged intermetallic distance of 6.11 Å. (b) Density of states (DOS) of Fe metal in FeCoNiRu-HESAC with the averaged intermetallic distance of 6.11 Å before and after interaction with OH*, indicating the bonding (σ) and antibonding (σ*) orbitals after interaction with OH*.

**Table S6.** DFT calculated Gibbs free energy (ΔG) of OH*, O*, OO*, OOH*, and H* intermediates for FeCoNiRu-SAC with several averaged intermetallic distances (Dist.).

| **Instances** | **Active atom** | **Dist. (Å)** | **Intermediate** | **ΔG (eV)** |
| --- | --- | --- | --- | --- |
| 1 | **Fe** | 15 | OH | 0.66 |
| 2 | **Fe** | 15 | O | 1.34 |
| 3 | **Fe** | 15 | OOH | 3.83 |
| 4 | **Fe** | 15 | OO | 4.39 |
| 5 | **Fe** | 15 | H | 0.33 |
| 6 | **Fe** | 10.68 | OH | 0.72 |
| 7 | **Fe** | 10.68 | O | 1.36 |
| 8 | **Fe** | 10.68 | OOH | 3.94 |
| 9 | **Fe** | 10.68 | OO | 4.42 |
| 10 | **Fe** | 10.68 | H | 0.36 |
| 11 | **Fe** | 9.15 | OH | 0.67 |
| 12 | **Fe** | 9.15 | O | 1.37 |
| 13 | **Fe** | 9.15 | OOH | 3.94 |
| 14 | **Fe** | 9.15 | OO | 4.43 |
| 15 | **Fe** | 9.15 | H | 0.37 |
| 16 | **Fe** | 6.11 | OH | 0.78 |
| 17 | **Fe** | 6.11 | O | 1.58 |
| 18 | **Fe** | 6.11 | OOH | 4.14 |
| 19 | **Fe** | 6.11 | OO | 4.57 |
| 20 | **Fe** | 6.11 | H | 0.49 |
| 21 | **Fe** | 4.81 | OH | 0.39 |
| 22 | **Fe** | 4.81 | O | 1.45 |
| 23 | **Fe** | 4.81 | OOH | 3.87 |
| 24 | **Fe** | 4.81 | OO | 4.38 |
| 25 | **Fe** | 4.81 | H | 0.46 |
| 26 | **Fe** | 2.93 | OH | -0.38 |
| 27 | **Fe** | 2.93 | O | 0.42 |
| 28 | **Fe** | 2.93 | OOH | 3.26 |
| 29 | **Fe** | 2.93 | OO | 3.3 |
| 30 | **Fe** | 2.93 | H | 0.22 |
| 31 | **Co** | 15 | OH | 1.05 |
| 32 | **Co** | 15 | O | 2.61 |
| 33 | **Co** | 15 | OOH | 4.24 |
| 34 | **Co** | 15 | OO | 4.59 |
| 35 | **Co** | 15 | H | 0.19 |
| 36 | **Co** | 10.68 | OH | 1.05 |
| 37 | **Co** | 10.68 | O | 2.57 |
| 38 | **Co** | 10.68 | OOH | 4.15 |
| 39 | **Co** | 10.68 | OO | 4.55 |
| 40 | **Co** | 10.68 | H | 0.18 |
| 41 | **Co** | 9.15 | OH | 1.05 |
| 42 | **Co** | 9.15 | O | 2.56 |
| 43 | **Co** | 9.15 | OOH | 4.2 |
| 44 | **Co** | 9.15 | OO | 4.54 |
| 45 | **Co** | 9.15 | H | 0.19 |
| 46 | **Co** | 6.11 | OH | 1.42 |
| 47 | **Co** | 6.11 | O | 2.46 |
| 48 | **Co** | 6.11 | OOH | 4.73 |
| 49 | **Co** | 6.11 | OO | 4.91 |
| 50 | **Co** | 6.11 | H | 0.54 |
| 51 | **Co** | 4.81 | OH | 1.11 |
| 52 | **Co** | 4.81 | O | 1.86 |
| 53 | **Co** | 4.81 | OOH | 4.27 |
| 54 | **Co** | 4.81 | OO | 4.66 |
| 55 | **Co** | 4.81 | H | 0.45 |
| 56 | **Co** | 2.93 | OH | -0.01 |
| 57 | **Co** | 2.93 | O | 0.97 |
| 58 | **Co** | 2.93 | OOH | 3.39 |
| 59 | **Co** | 2.93 | OO | 3.49 |
| 60 | **Co** | 2.93 | H | 0.11 |
| 61 | **Ni** | 15 | OH | 1.93 |
| 62 | **Ni** | 15 | O | 4.06 |
| 63 | **Ni** | 15 | OOH | 5.06 |
| 64 | **Ni** | 15 | OO | 5.31 |
| 65 | **Ni** | 15 | H | 1.61 |
| 66 | **Ni** | 10.68 | OH | 1.9 |
| 67 | **Ni** | 10.68 | O | 3.87 |
| 68 | **Ni** | 10.68 | OOH | 5.03 |
| 69 | **Ni** | 10.68 | OO | 5.28 |
| 70 | **Ni** | 10.68 | H | 1.43 |
| 71 | **Ni** | 9.15 | OH | 1.94 |
| 72 | **Ni** | 9.15 | O | 3.82 |
| 73 | **Ni** | 9.15 | OOH | 5.07 |
| 74 | **Ni** | 9.15 | OO | 5.33 |
| 75 | **Ni** | 9.15 | H | 1.36 |
| 76 | **Ni** | 6.11 | OH | 2.01 |
| 77 | **Ni** | 6.11 | O | 3.57 |
| 78 | **Ni** | 6.11 | OOH | 5.15 |
| 79 | **Ni** | 6.11 | OO | 5.46 |
| 80 | **Ni** | 6.11 | H | 1.71 |
| 81 | **Ni** | 4.81 | OH | 1.27 |
| 82 | **Ni** | 4.81 | O | 2.6 |
| 83 | **Ni** | 4.81 | OOH | 4.48 |
| 84 | **Ni** | 4.81 | OO | 4.82 |
| 85 | **Ni** | 4.81 | H | 0.96 |
| 86 | **Ni** | 2.93 | OH | -0.04 |
| 87 | **Ni** | 2.93 | O | 1.49 |
| 88 | **Ni** | 2.93 | OOH | 3.34 |
| 89 | **Ni** | 2.93 | OO | 3.61 |
| 90 | **Ni** | 2.93 | H | -0.13 |
| 91 | **Ru** | 15 | OH | 0.17 |
| 92 | **Ru** | 15 | O | 0.7 |
| 93 | **Ru** | 15 | OOH | 3.34 |
| 94 | **Ru** | 15 | OO | 3.66 |
| 95 | **Ru** | 15 | H | -0.45 |
| 96 | **Ru** | 10.68 | OH | 0.12 |
| 97 | **Ru** | 10.68 | O | 0.6 |
| 98 | **Ru** | 10.68 | OOH | 3.18 |
| 99 | **Ru** | 10.68 | OO | 4.06 |
| 100 | **Ru** | 10.68 | H | -0.49 |
| 101 | **Ru** | 9.15 | OH | 0.16 |
| 102 | **Ru** | 9.15 | O | 0.66 |
| 103 | **Ru** | 9.15 | OOH | 3.27 |
| 104 | **Ru** | 9.15 | OO | 3.71 |
| 105 | **Ru** | 9.15 | H | -0.5 |
| 106 | **Ru** | 6.11 | OH | 0.48 |
| 107 | **Ru** | 6.11 | O | 0.98 |
| 108 | **Ru** | 6.11 | OOH | 3.54 |
| 109 | **Ru** | 6.11 | OO | 3.98 |
| 110 | **Ru** | 6.11 | H | -0.31 |
| 111 | **Ru** | 4.81 | OH | 0.6 |
| 112 | **Ru** | 4.81 | O | 1.33 |
| 113 | **Ru** | 4.81 | OOH | 3.83 |
| 114 | **Ru** | 4.81 | OO | 4.24 |
| 115 | **Ru** | 4.81 | H | -0.23 |
| 116 | **Ru** | 2.93 | OH | -0.37 |
| 117 | **Ru** | 2.93 | O | 0.06 |
| 118 | **Ru** | 2.93 | OOH | 3.09 |
| 119 | **Ru** | 2.93 | OO | 3.19 |
| 120 | **Ru** | 2.93 | H | -0.22 |

**S2. Machine Learning and data analysis**

We use the support vector repression (SVR) as a supervised machine learning (ML) algorithm^[5]^ to calculate Gibbs free energy of different intermediates using data from the DFT calculations. We use Scikit-learn, NumPy, Matplotlib, SHAP, Pickle, and SciPy libraries in Python 3.6 to read and process the data, train and save the ML algorithm, and perform feature importance analysis. In order to have high-quality data analysis and an interpretable ML algorithm, we need physically interpretable input features with high simplicity and reasonable feature importance.^[6]^ Accordingly, the input features used in our ML model include the intrinsic properties of metal atom (M), substrate, and intermediates such as atomic radius (AtR_M_), atomic number (AtN_M_), electronegativity (EN_M_), first ionization energy (IE_M_), electron affinity (EA_M_), numbers of electron in d orbitals of the active metal site (θ_d,M_), intermetallic distance (Dist.), and valence electron number of reaction intermediate The input dataset contains a total of 120 DFT-predicted Gibbs free energy of H*, OH*, O*, OOH*, and OO* bonded. To various metal sites. The input data was randomally partitioned into the training set (90%, 108 data points) and the test set (10%, 12 data points). The training set was used to construct the ML model, and we used the root mean squared error (RMSE, equation S9) and R^2^ value to evaluate the performance of the SVR model:^[5]^

| $RMSE=\sqrt{\frac{1}{n}\sum_{i=1}^{n} \left( {\Delta G}_{DFT,i}-{\Delta G}_{ML,i} \right)^{2}}$ | (S9) |
| --- | --- |

where ΔG_DFT,i_ and ΔG_ML,i_ are the DFT- and ML-calculated Gibbs free energies, respectively, for intermediate i, and n is the number of instances in the training data set. Based on the trained SVR model, several methods such as Shapley Additive exPlanation (SHAP),^[7]^ permutation,^[8]^ mutual information (MI), and Pearson correlation coefficient^[9]^ were applied to screen the impact of each input features on the model output.


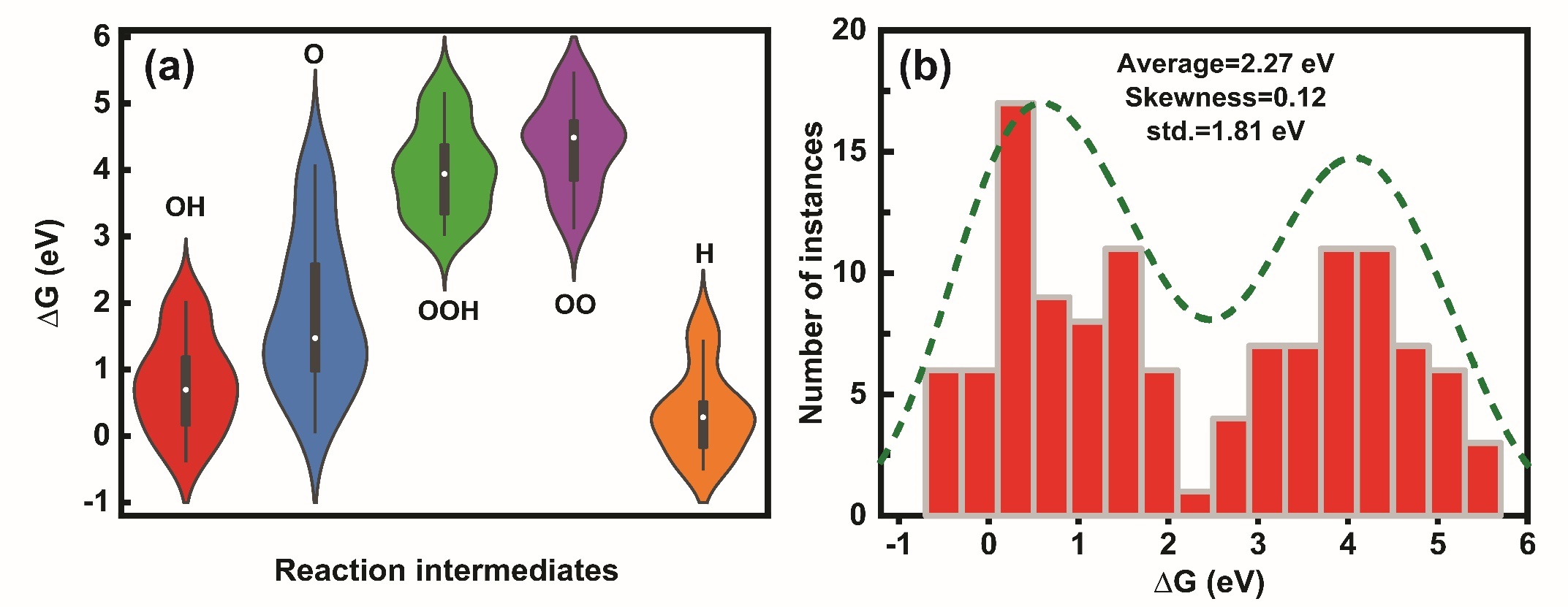


**Figure S15. Input data analysis for machine learning (ML).** (a) Violin plot of Gibbs free energies (ΔG, eV) distribution for OH*, O*, OOH*, OO*, and H* reaction intermediates for the high entropy single atom catalysts (HESAC), indicating a total of 120 input data. (b) The histogram of variation of Gibbs free energies indicates the average, skewness, and standard deviation (std.) of 2.27 eV, 0.12 and 1.81 eV, respectively.


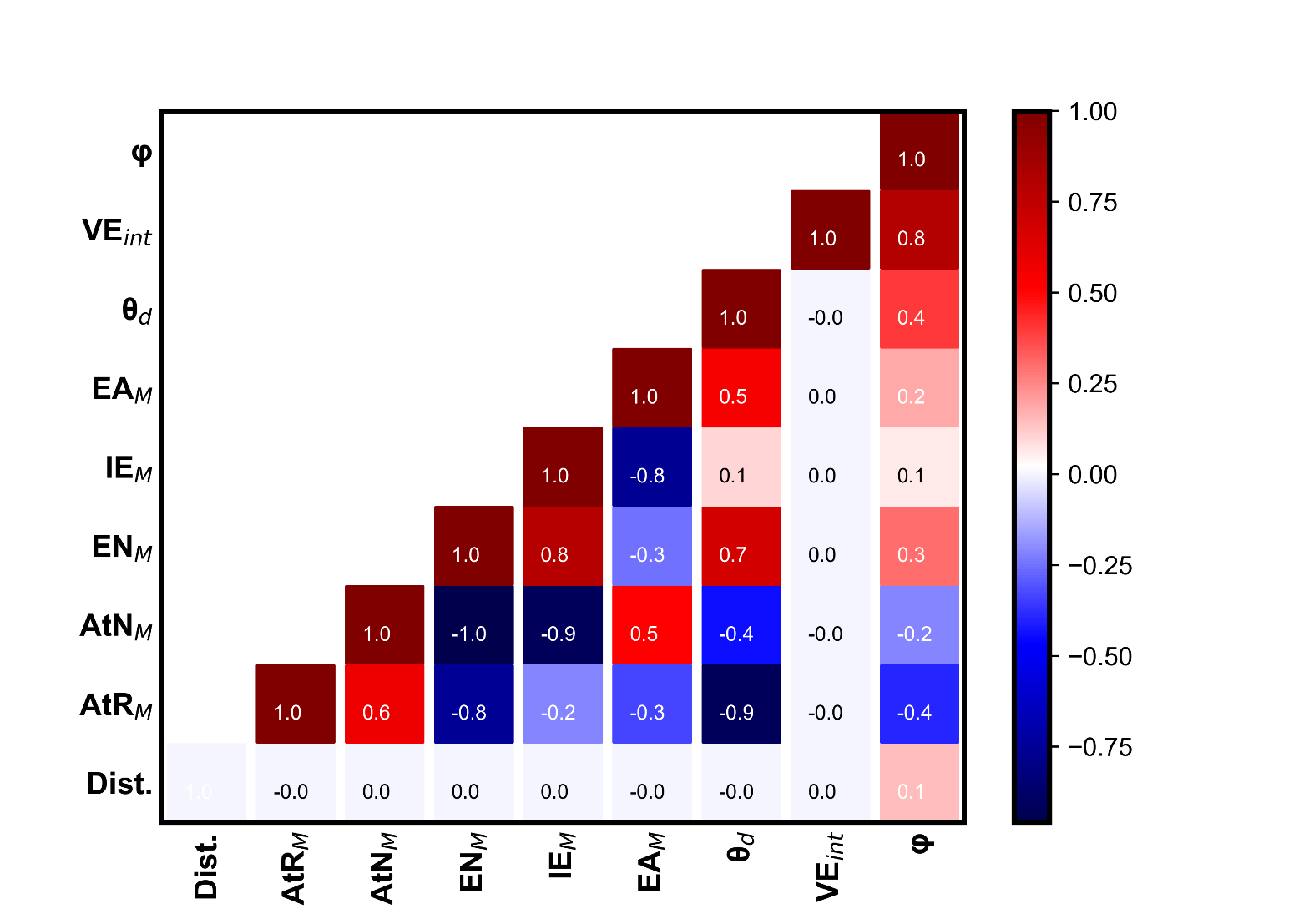


**Figure S16.** Feature-feature correlation map of the input features.


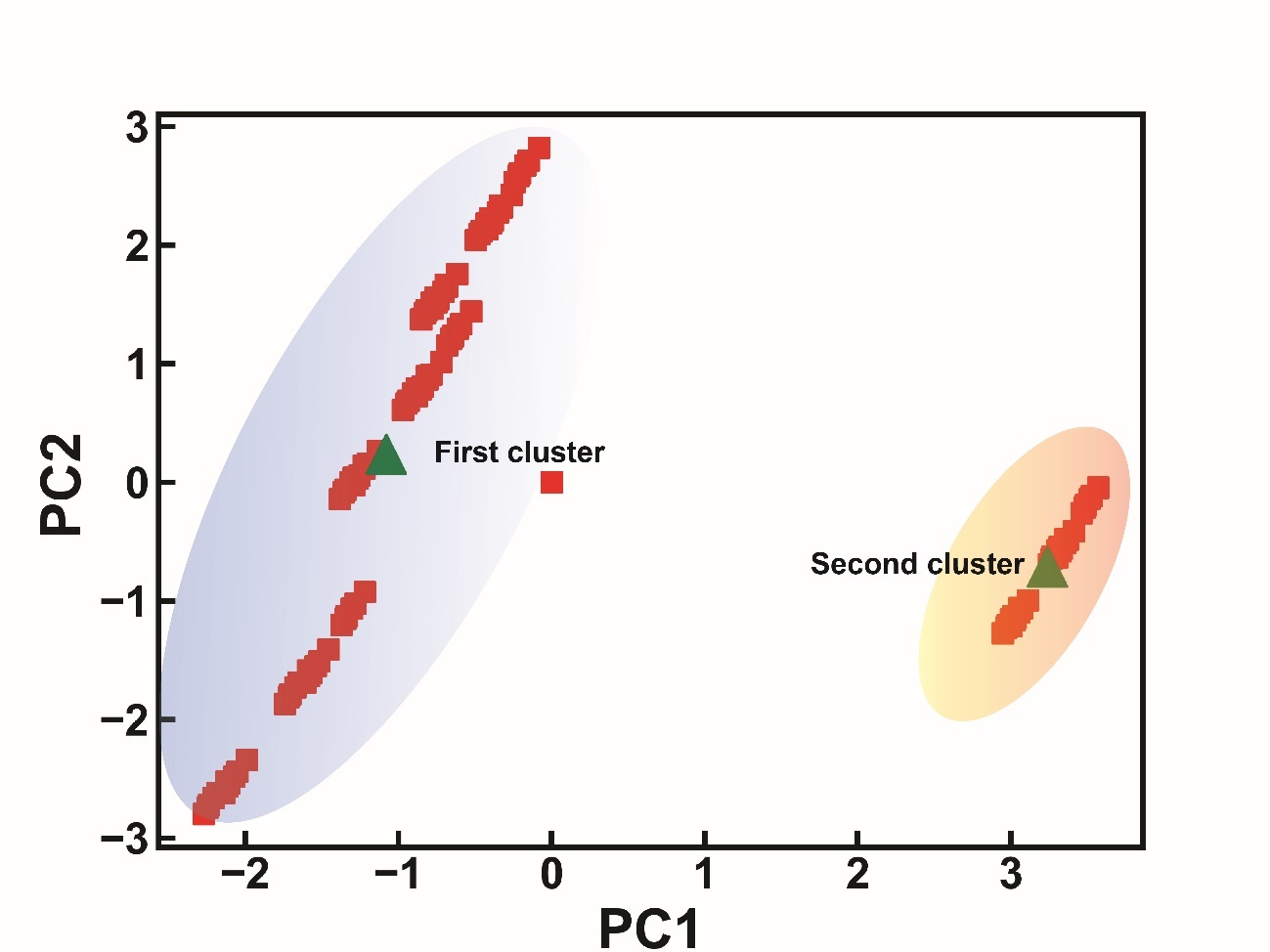


**Figure S17.** Principal component analysis (PCA) and K-means clustering for the DFT-calculated data (■) projected onto PC1-PC2 plane. PC1 and PC2 stand for the first and second principal components, respectively. The k-means clustering method suggests two main clusters in the PC1-PC2 plane with the centers shown in green triangle (▲). 0.419+0.267


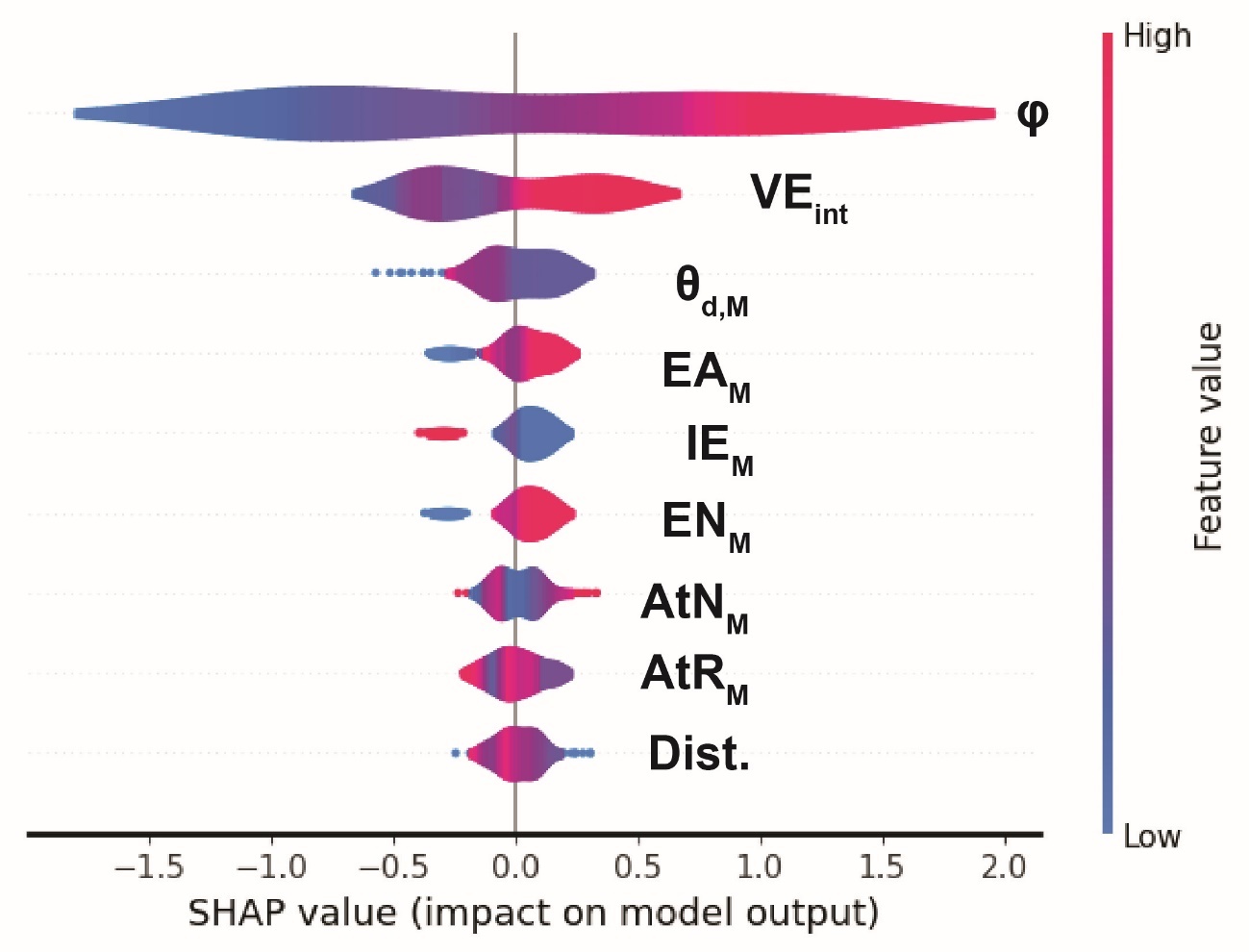


**Figure S18.** Violin plot of SHAP values for input features colored based on the features value.


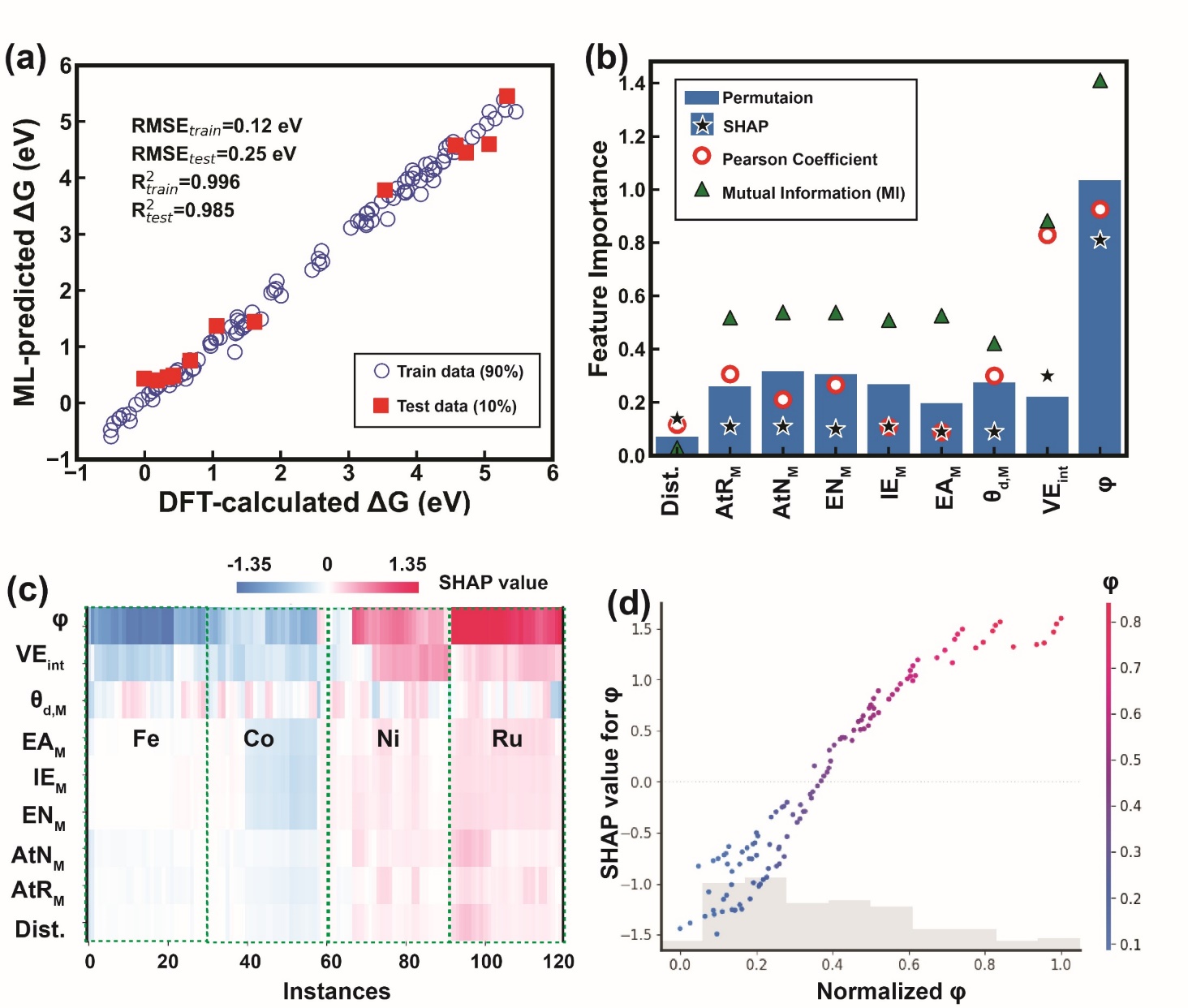


**Figure S19. Machine learning implementation.** (a) The parity plot of ML-predicted versus DFT-calculated Gibbs free energy of reaction intermediates such as H*, OH*, O*, OOH*, and OO* for FeCoNiRu-HESAC. The support vector regression (SVR) algorithm shows satisfactory MSE and R^2^ values for both training and test data without any signs of underfitting. (b) Feature importance analysis on the Gibbs free energy (ΔG) of reaction intermediates based on the permutation and SHAP methods along with the corresponding Mutual Information (MI) and Pearson correlation coefficients (solid red circle). This indicates that the new descriptor (φ), valence electron of intermediate (VE_int_), and number of d electrons (θd) are the most important parameters. (c) The heatmap of SHAP values of input feature for the whole 80 data instances in the order of feature importance, indicating the high effect of Fe and Ru atoms on the adsorption energies. (c) SHAP value of new descriptor (φ) vs. the value of new descriptor (φ), colored based on the features’ values, indicating nearly linear dependence of SHAP value to the new descriptor (φ).

**S3. Experimental synthesis and characterization**


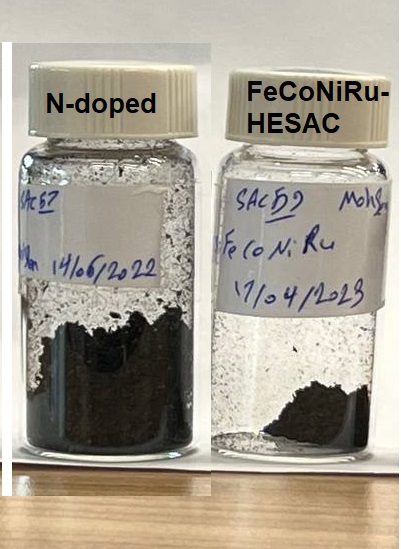


**Figure S20.** Prepared N-doped and FeCoNiRu-HESAC samples.

**(d)**

**(a)**

**(c)**

**Figure S21. XPS results for N-doped sample.** (a) Wide range XPS spectra of N-doped sample. (b) The N 1s XPS spectra with three visible N species belong to pyridinic-N (398.1 eV), pyrrolic-N (399.6 eV), and N-oxide (407.1 eV). (c) The C 1s XPS spectra with two visible C species belong to C-C (284.3 eV) and C-O (286.4 eV).

| **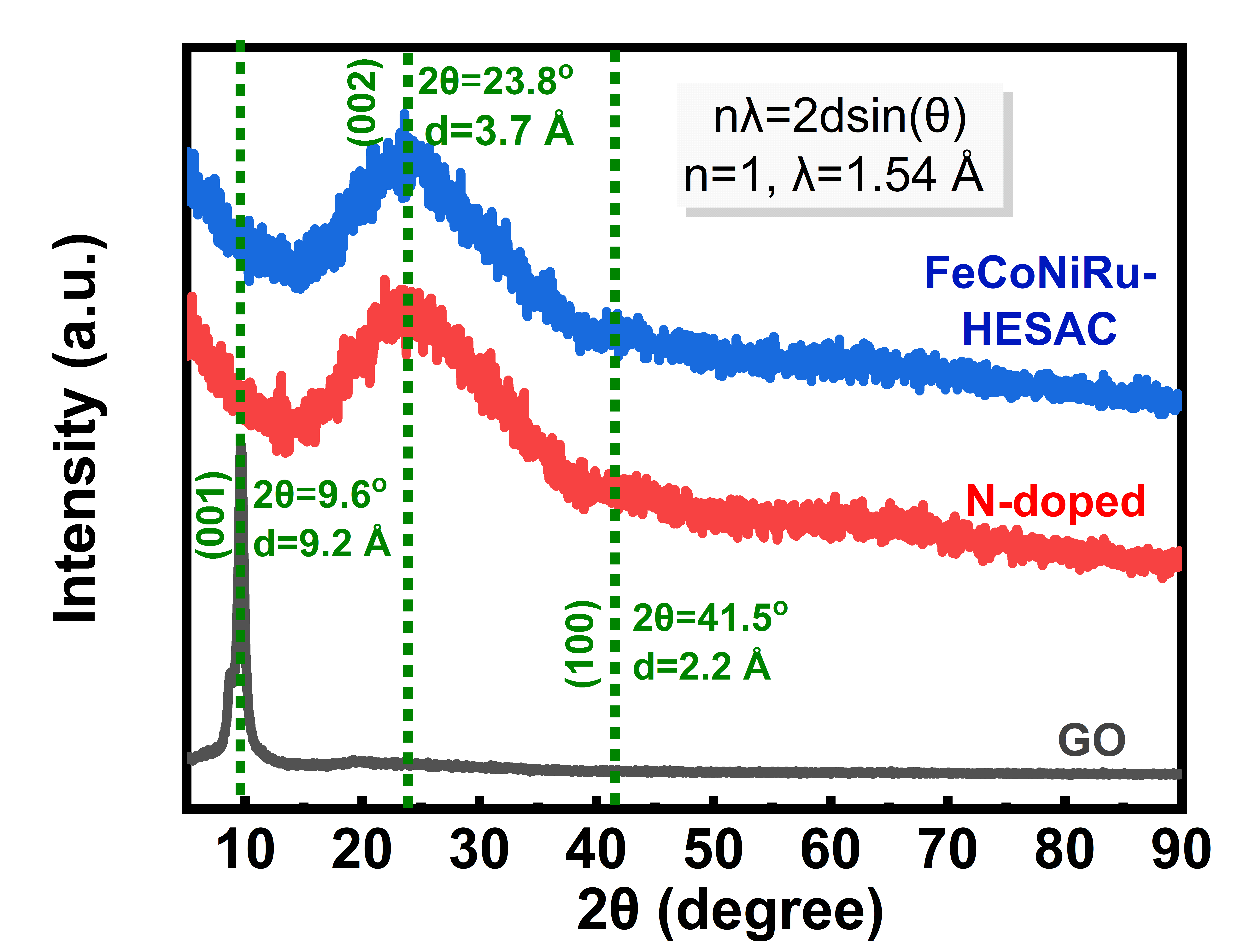**  **(a)** | **(b)**  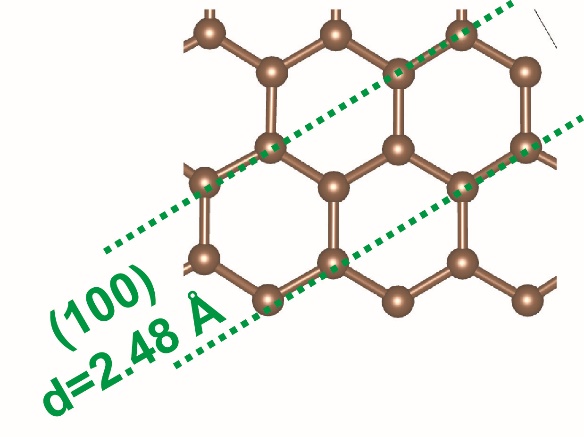 |
| --- | --- |

**Figure S22.** (a) XRD pattern of synthesized graphene oxide (GO), N-doped, and FeCoNiRu-HESAC. The peak at 23.8^o^ belongs to the graphitic carbon peak (002) ^[10]^. In both samples, the broad graphitic peak is observed, ensuring the samples’ polycrystalline crystal structure. No peak corresponds to metal species in any samples due to a small amount of metal atoms. Based on our XPS measurements, the loading of metal species in each sample is less than 0.4 wt.%. (b) Representative of (100) plane of GO.

The interlayer spacing can be calculated based on Bragg's Law:

| $n\lambda=2dsin\left( \theta\right)$ | (S9) |
| --- | --- |

So:

| $d=9.2 Å$ | for GO |
| --- | --- |
| $d=3.7 Å$ | for N-doped |
| $d=3.7 Å$ | for FeCoNiRu-HESAC |


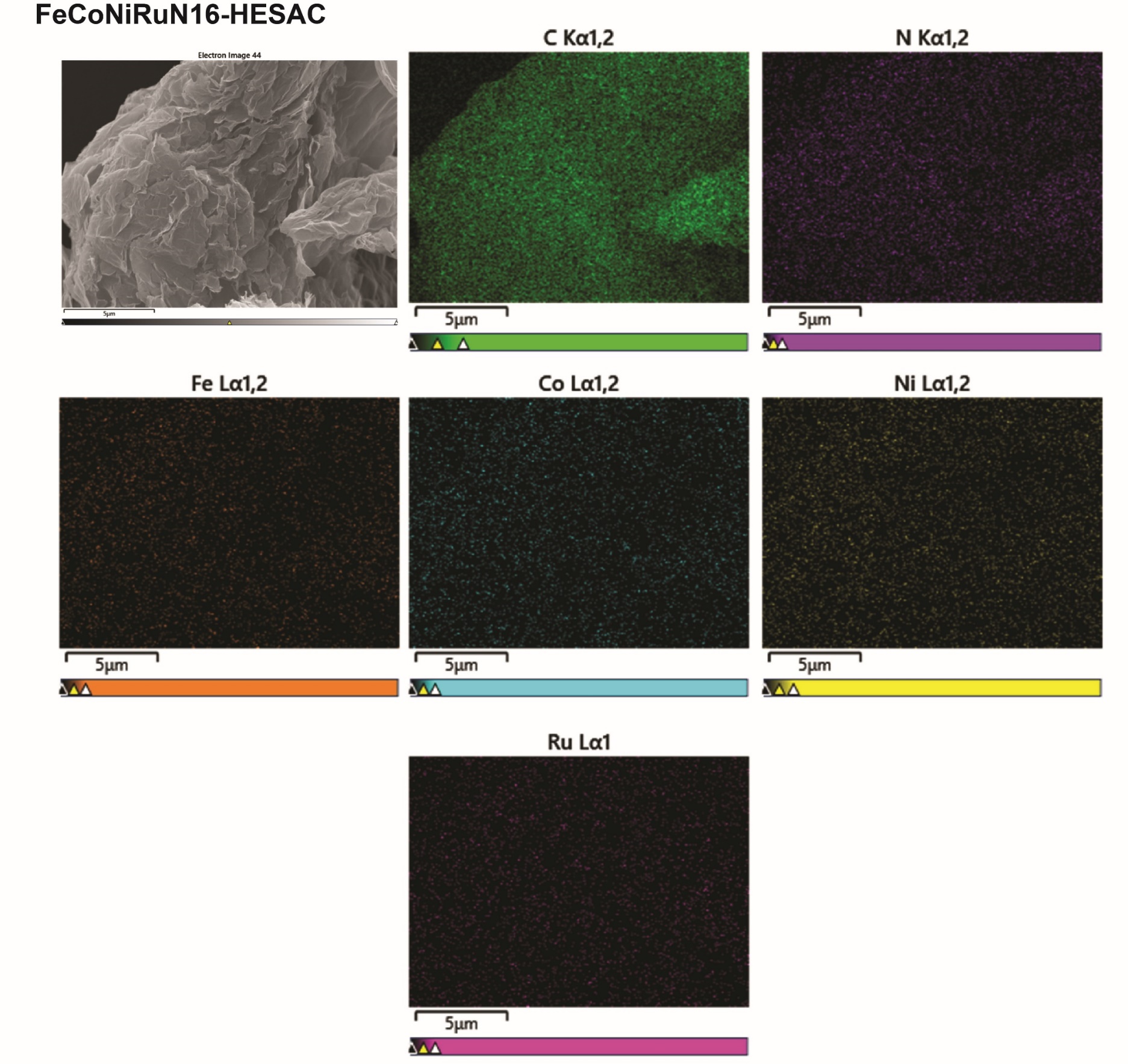


**Figure S23.** SEM imaging and EDX elemental mapping of FeCoNiRu-DAC sample for C, N, Fe, Co, Ni, and Ru elements, indicating the uniform distribution of elements on the surface.

| 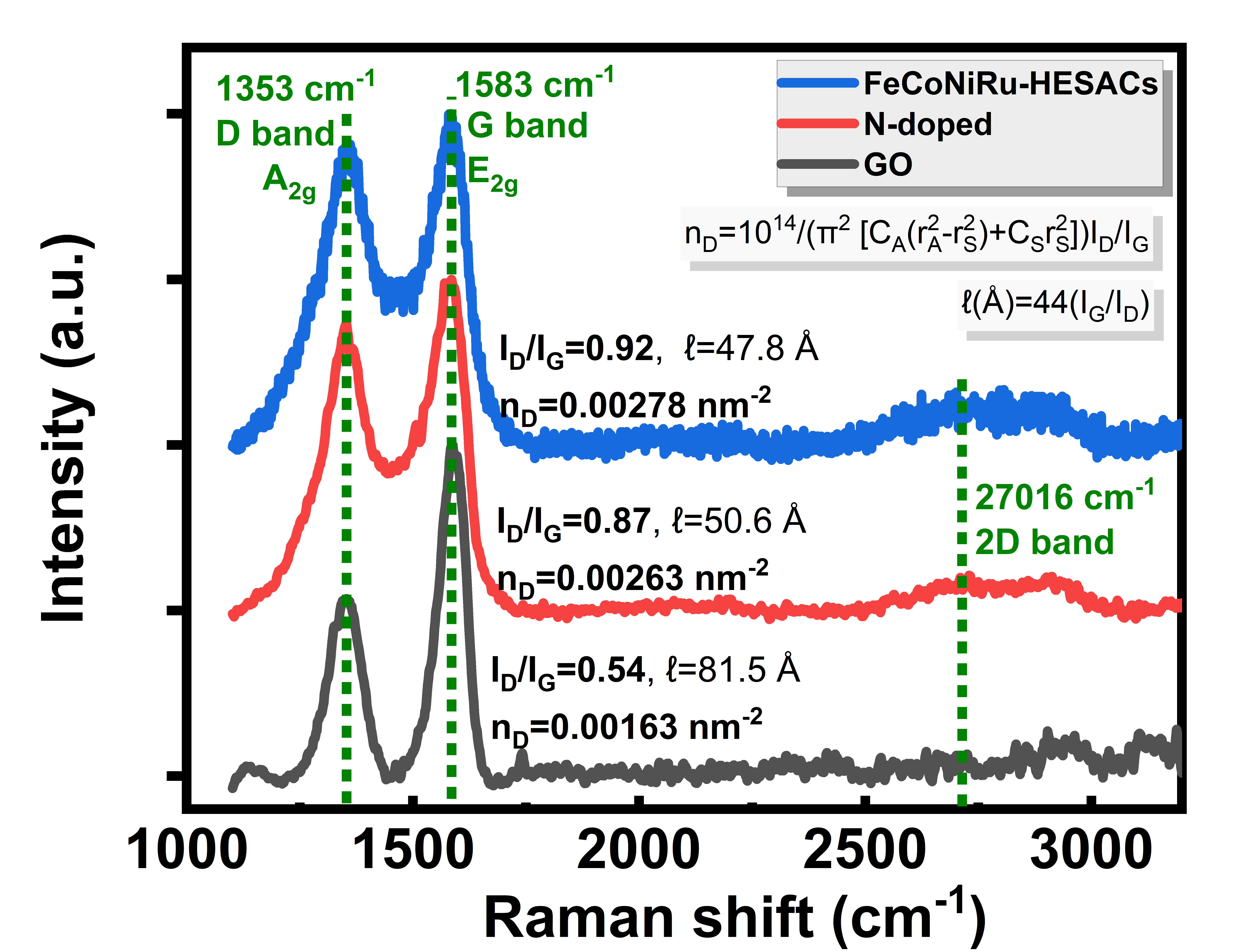  **(a)** | **(b)**  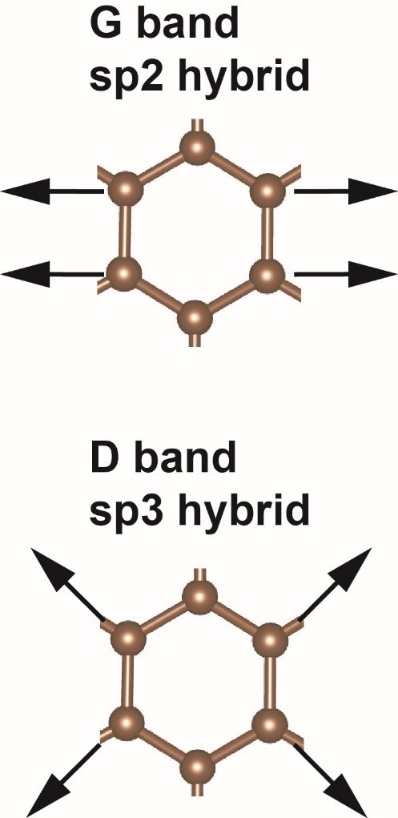 |
| --- | --- |

**Figure S24.** (a) Raman spectroscopy of graphene oxide (GO), N-doped, and FeCoNiRu-HESAC. The increase in the D peak intensity indicates the greater amount of defects present in the catalyst. (b) Representative of G and D bands showing the planar configuration sp^2^ and sp^3^ bonded carbon atoms.


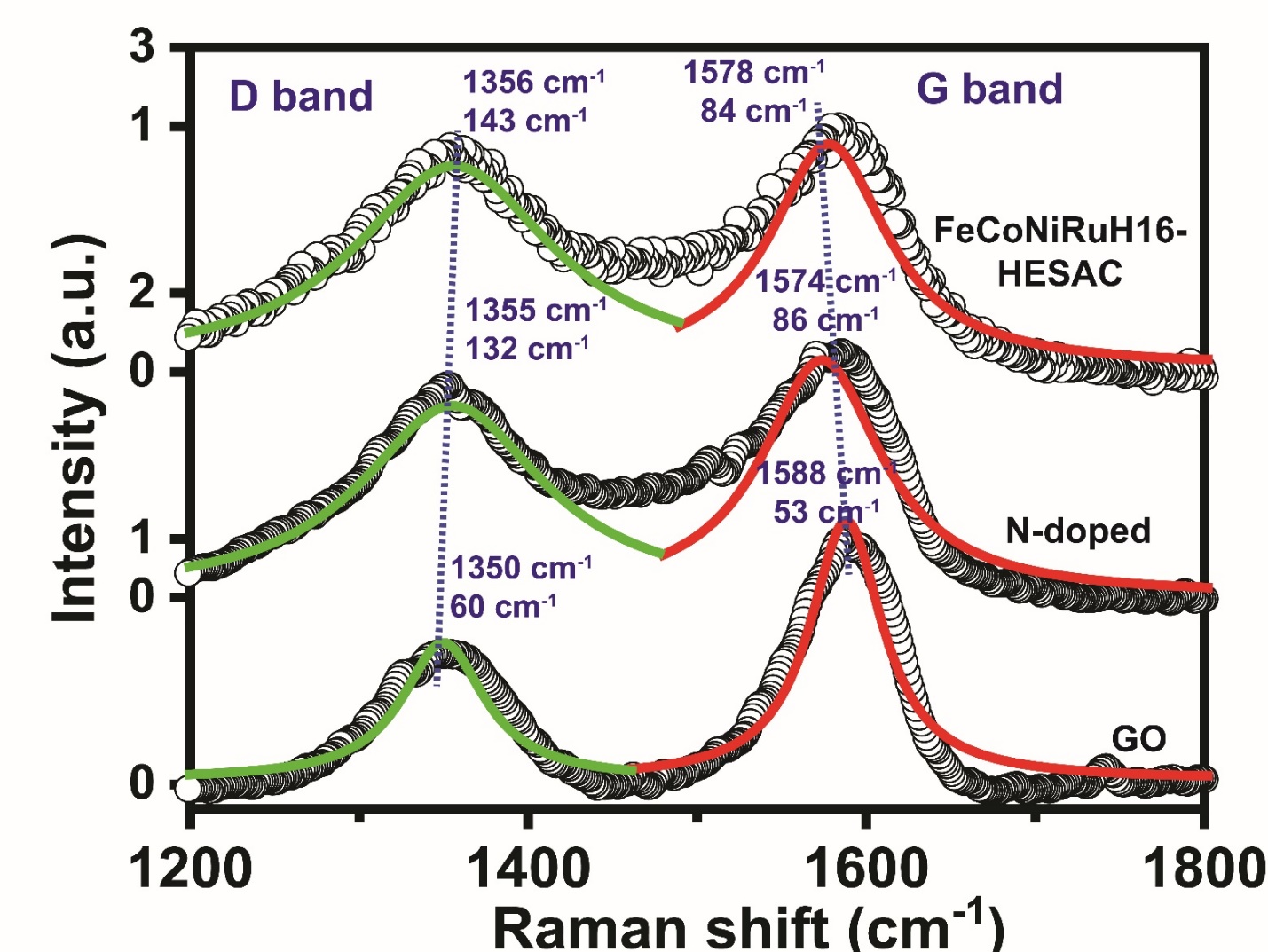


**Figure S25.** High resolution Raman spectroscopy of graphene oxide (GO), N-doped, and FeCoNiRu-HESAC.

The defect density (n_D_) can be calculated using the following equation:^[11–13]^

| $n_{D}\left( cm^{-2} \right)=\frac{{10}^{14}}{\pi^{2}\left[ C_{A}\left( r_{A}^{2}-r_{S}^{2} \right)+C_{S}r_{S}^{2} \right]}\left( \frac{I_{D}}{I_{G}} \right)$ | (S10) |
| --- | --- |

Where for the D peak, the reported values are approximately given as C_A_ = 4.2, C_S_ = 0, r_A_ = 3 nm, and r_S_ = 1 nm,^[11]^ so:

| $n_{D}\left( \mathrm{nm}^{-2} \right)=0.00268\left( \frac{I_{D}}{I_{G}} \right)$ | (S11) |
| --- | --- |
| and:   \| $n_{D}=1.63\times{10}^{-3} \mathrm{nm}^{-2}$ \| for GO \| \| --- \| --- \| \| $n_{D}=2.63\times{10}^{-3} \mathrm{nm}^{-2}$ \| for N-doped \| \| $n_{D}=2.78\times{10}^{-3} \mathrm{nm}^{-2}$ \| for FeCoNiRu-HESAC \| |  |

The density of defect vacancies (n_D_) for N-doped and FeCoNiRu-HESAC increases by 61% and 70%, respectively, compared to GO.

The interdefects spacing can be also calculated using the following equation:

| $\mathcal{l}\left( Å \right)=44\left( \frac{I_{G}}{I_{D}} \right)$ | (S12) |
| --- | --- |

so:

| $\mathcal{l=}81.5 Å$ | for GO |
| --- | --- |
| $\mathcal{l=}50.6 Å$ | for N-doped |
| $\mathcal{l=}47.8 Å$ | for FeCoNiRu-HESAC |

Also we can write:

| $\mathcal{l(Å)=}\frac{118}{n_{D}\left( \mathrm{nm}^{-2} \right)}$ | (S13) |
| --- | --- |


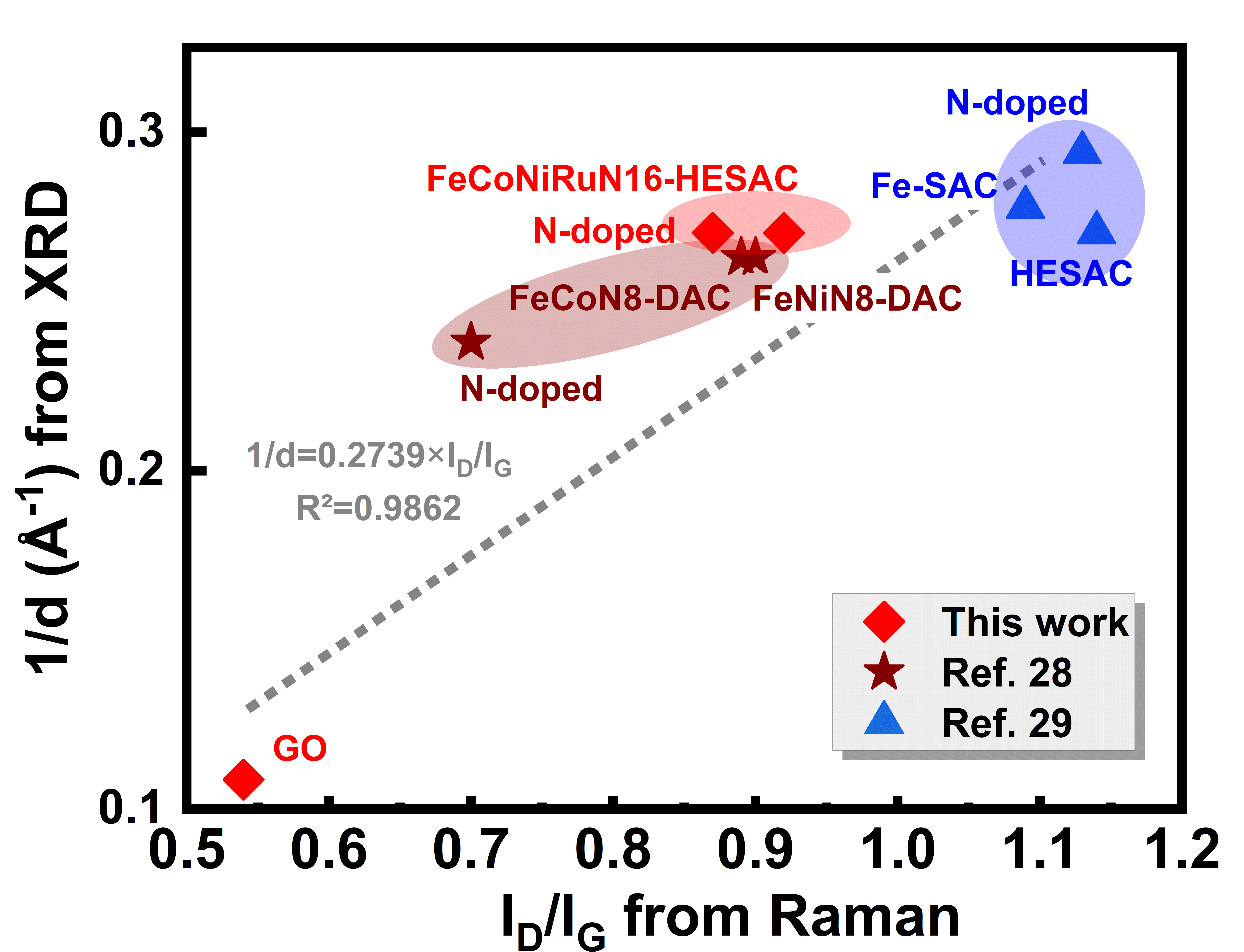


**Ref. [8]**

**Ref. [10]**

**Figure S26.** 1/d from XRD versus I_D_/I_G_ obtained from Raman spectroscopy for graphene oxide (GO), N-doped, and FeCoNiRu-HESAC along with FeCo-DAC, FeNi-DAC, and N-doped from Ref. ^[12]^ and HESAC, Fe-SAC, and N-doped from Ref. ^[14]^. The increase in I_D_/I_G_ indicates the decrease in the d.^[15]^

We can derive the interlayer spacing of samples based on the following equation which might be used for carbon-based materials:

| $d\left( Å \right)=3.65\left( \frac{I_{G}}{I_{D}} \right)$ | (S14) |
| --- | --- |

so:

| $\mathcal{l}\left( Å \right)=12\times d\left( Å \right)$ | (S15) |
| --- | --- |


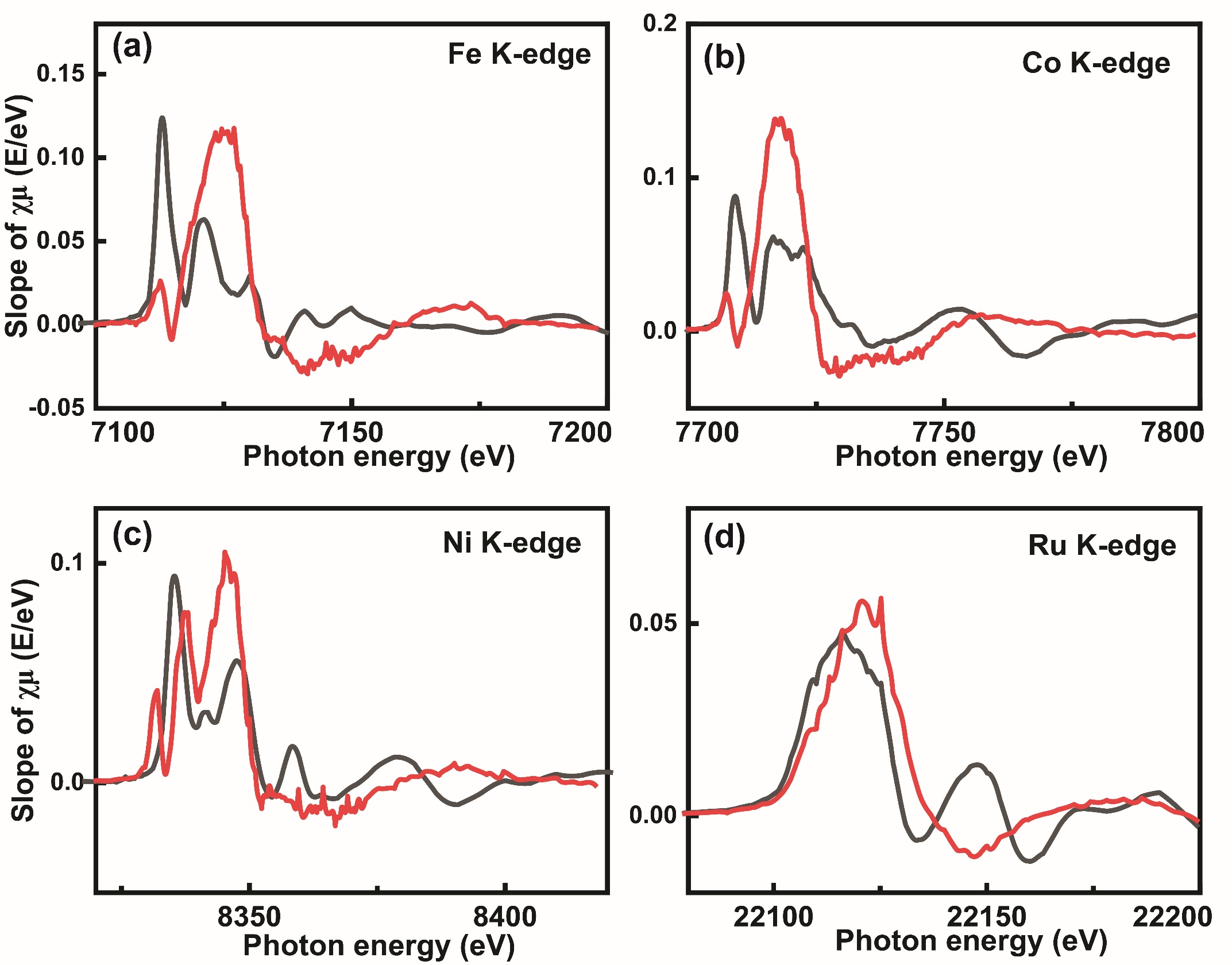


**Figure S27.** First derivative curves of Fe, Co, Ni, and Ru K-edge XANES spectra (red lines) with their reference bulk samples (black lines).


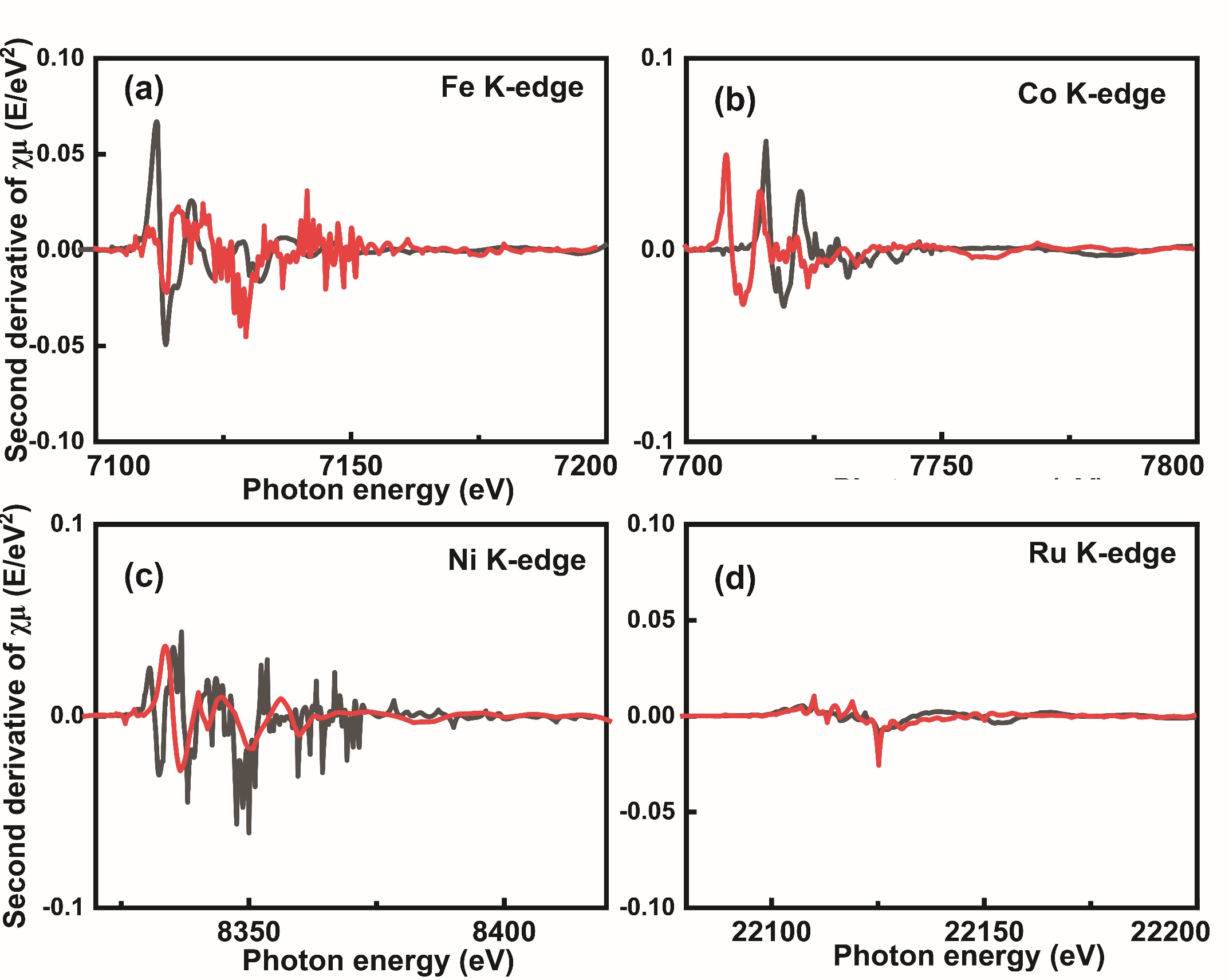


**Figure S28.** Second derivative curves of Fe, Co, Ni, and Ru K-edge XANES spectra (red lines) with their reference bulk samples (black lines).


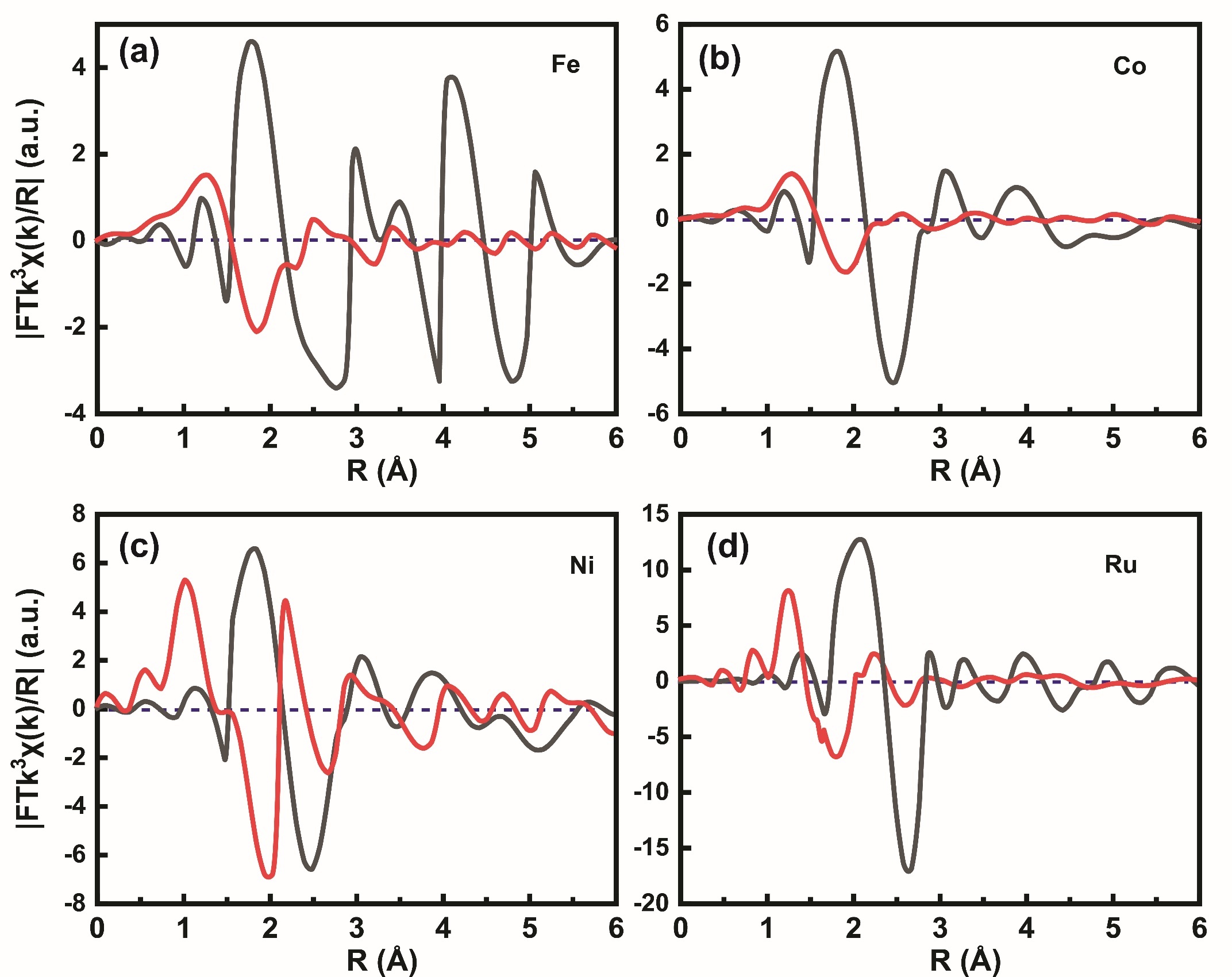


**Figure S29.** First derivative curves of Fe, Co, Ni, and Ru EXAFS spectra (red lines) with their reference bulk samples (black lines).


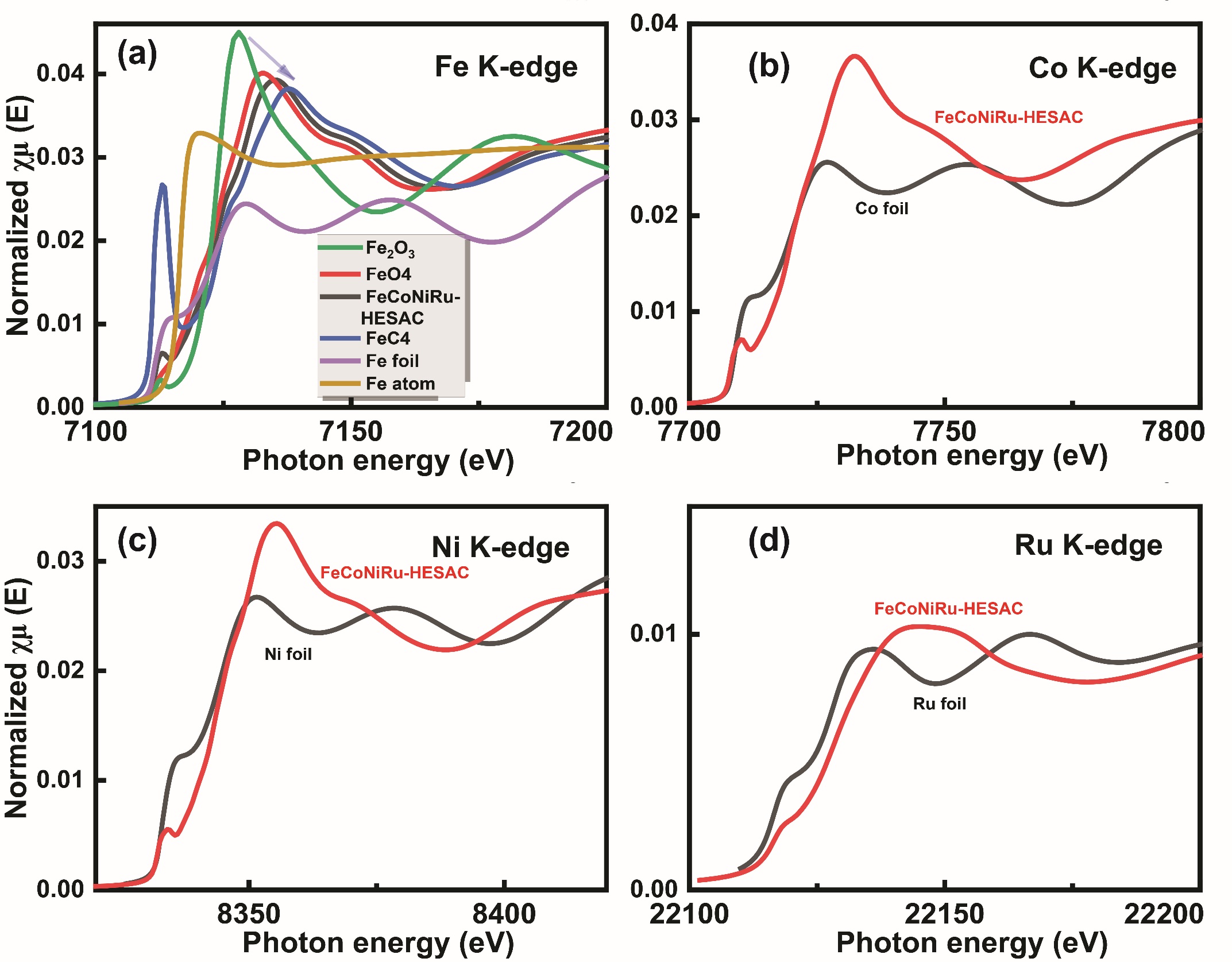


**Figure S30.** (a) Fe K-edge, (b) Co K-edge, (c) Ni K-edge, and (d) Ru K-edge XANES spectra of FeCoNiRu-HESAC (red lines) along with Fe, Co, Ni, and Ru foils (black lines), obtained theoretically using the accurate finite difference (FD) approach implemented in the FDMNES software.^[16,17]^


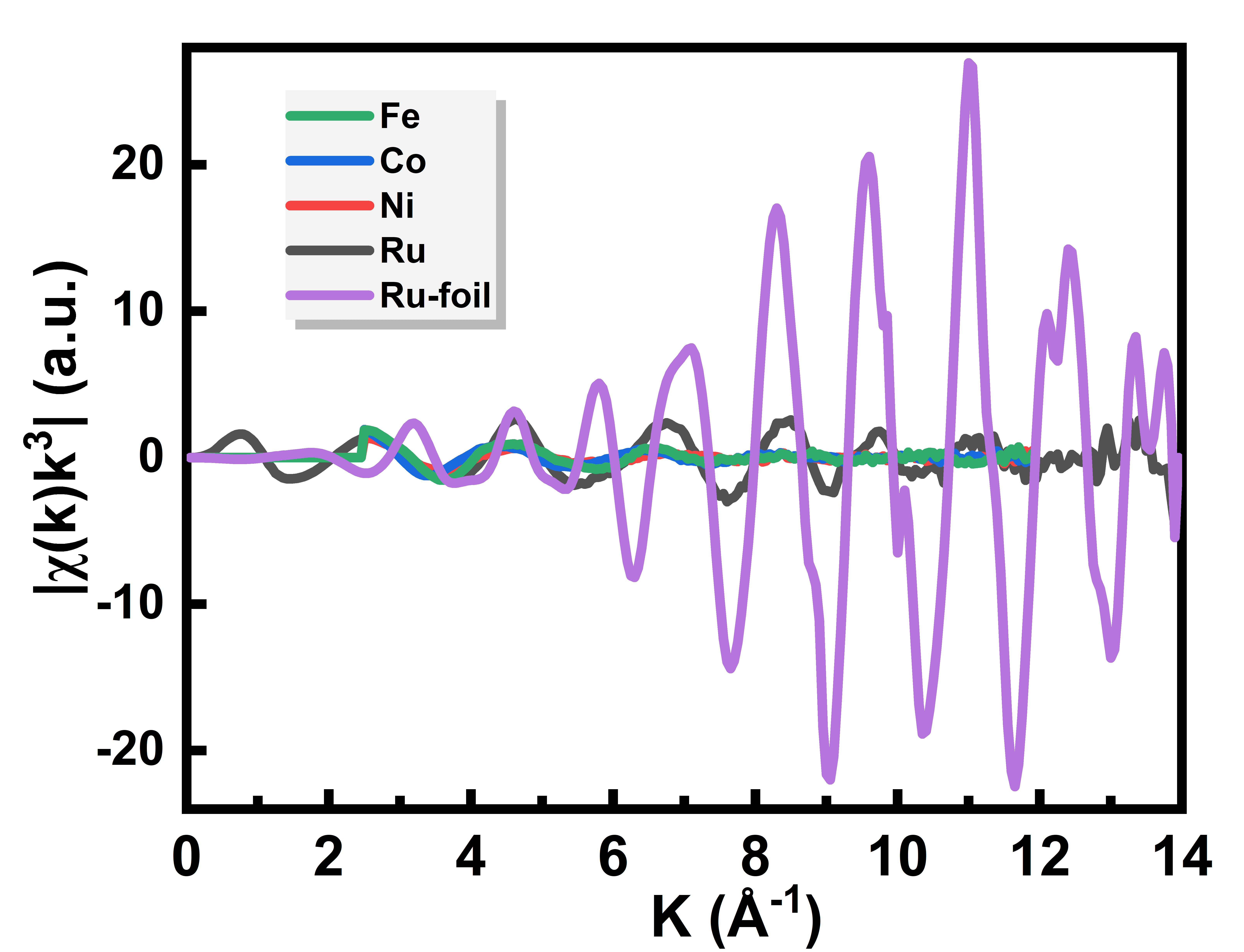


**Figure S31.** The EXAFS analysis of FeCoNiRu-HESAC sample at k space for Fe, Co, Ni, and Ru elements along with Ru foil.


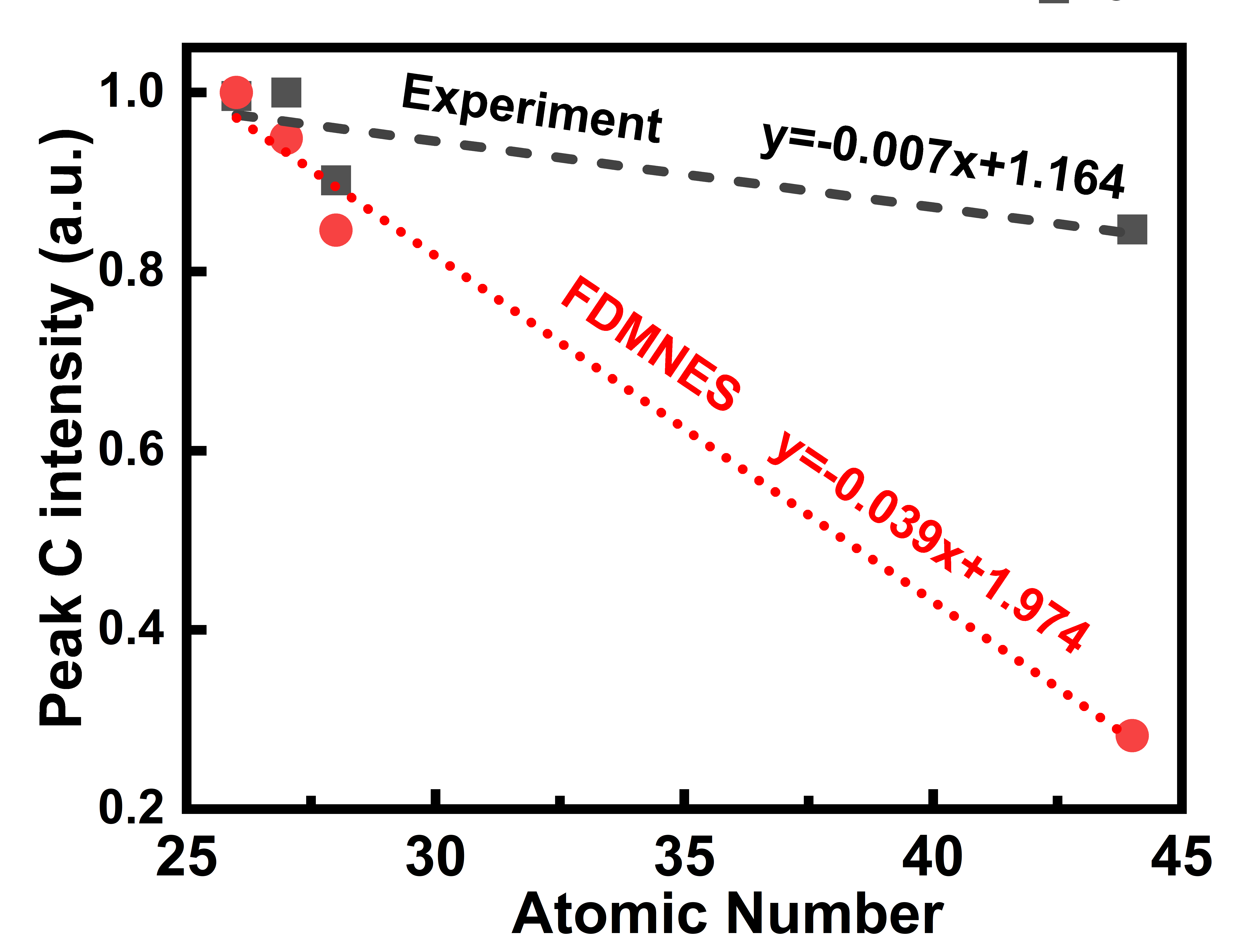


**Figure S32.** Peak intensity of XANES spectra versus the atomic number of Fe, Co, Ni, and Ru metals of FeCoNiRu-HESAC obtained experimentally and theoretically. This indicates that by increasing the atomic number, the peak intensity decreases.


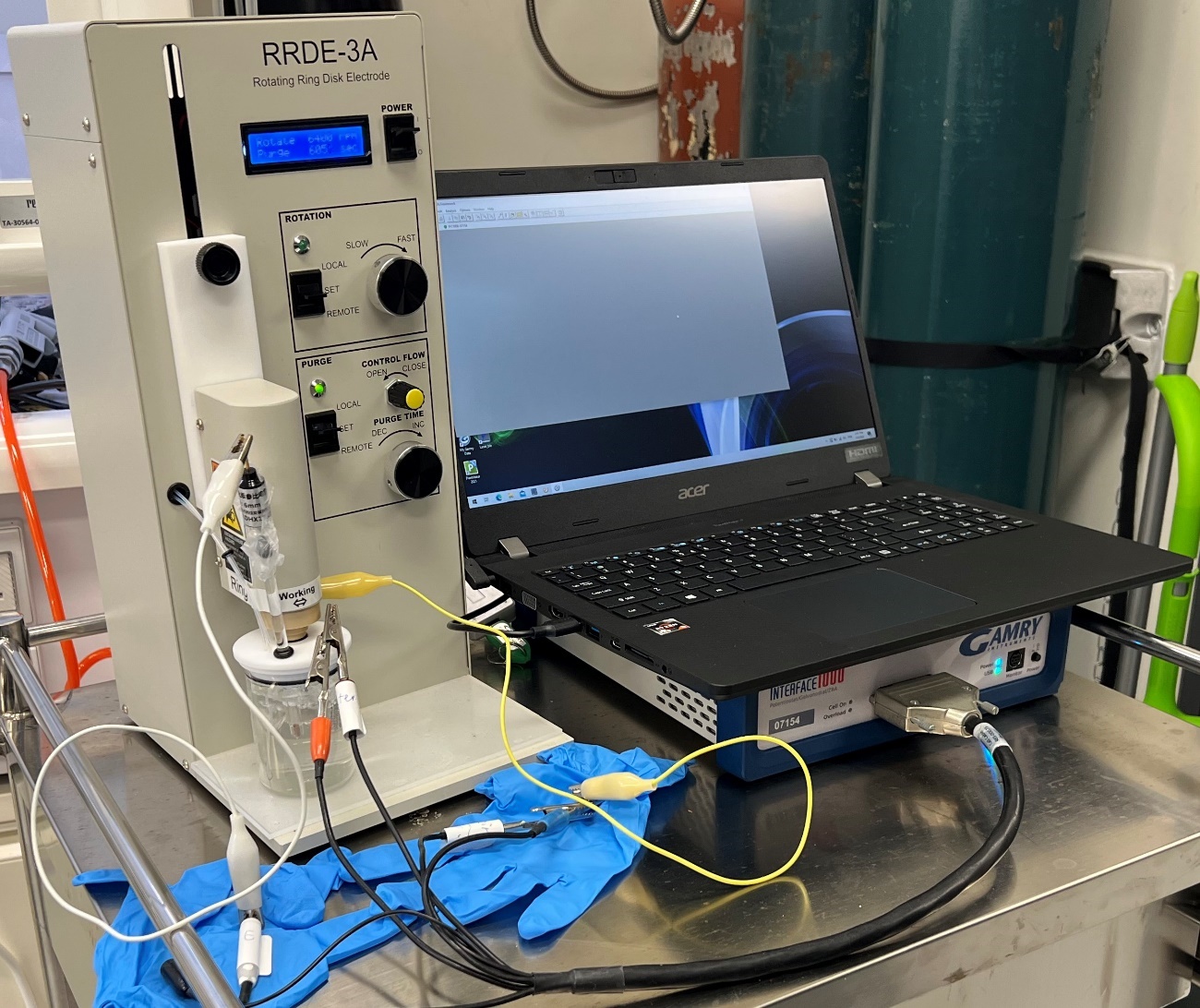


**Figure S33.** The Rotating Ring Disk Electrode device along with a three-electrode cell loaded with either 0.1 M KOH or 0.5 M H_2_SO_4_ aqueous electrolytes for the ORR performance measurements. The GCE loaded with catalysts was used as a working electrode, Pt wire was used as a counter electrode, and an Ag/AgCl electrode was used as a reference electrode which was prior filled in saturated 1 M KOH.


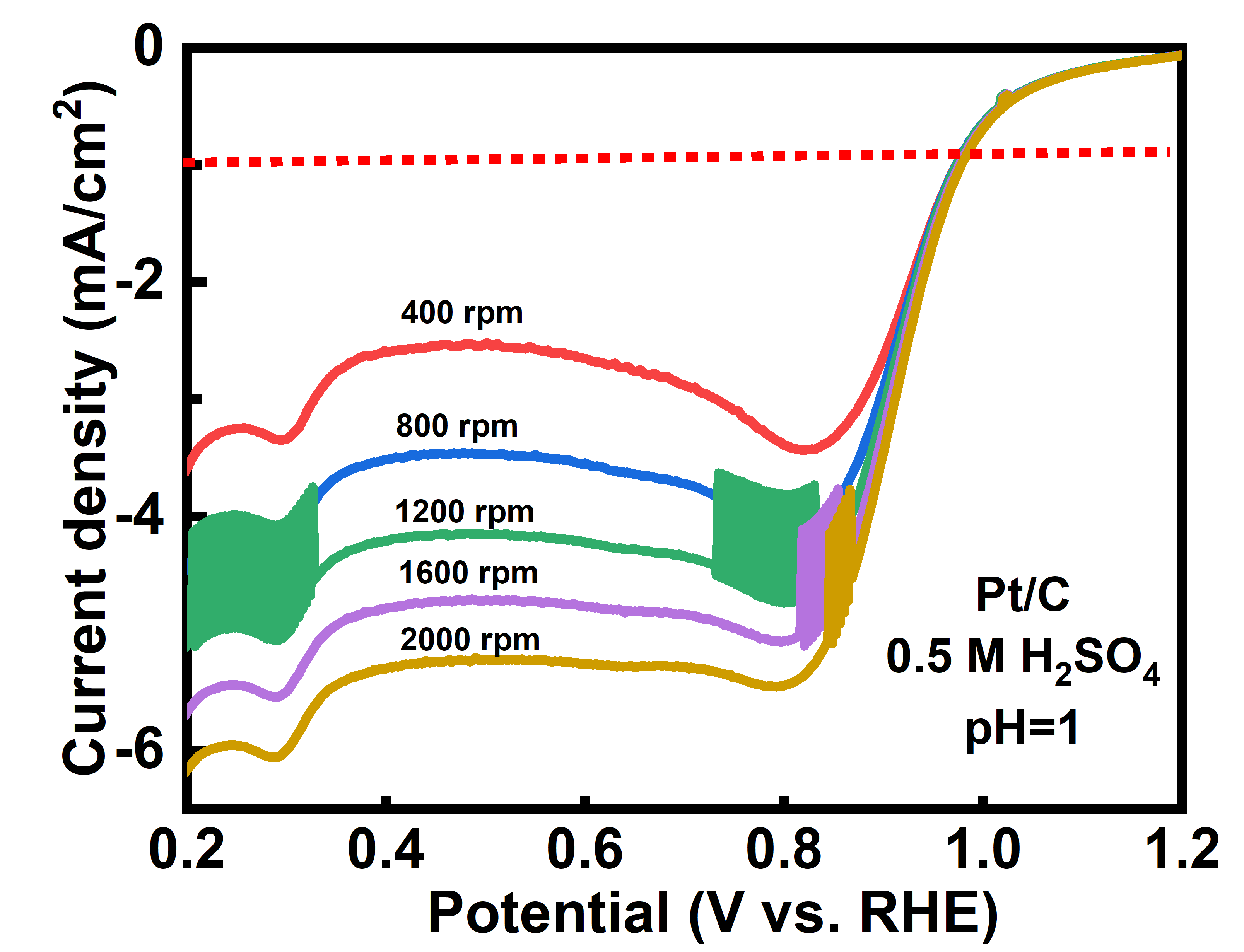

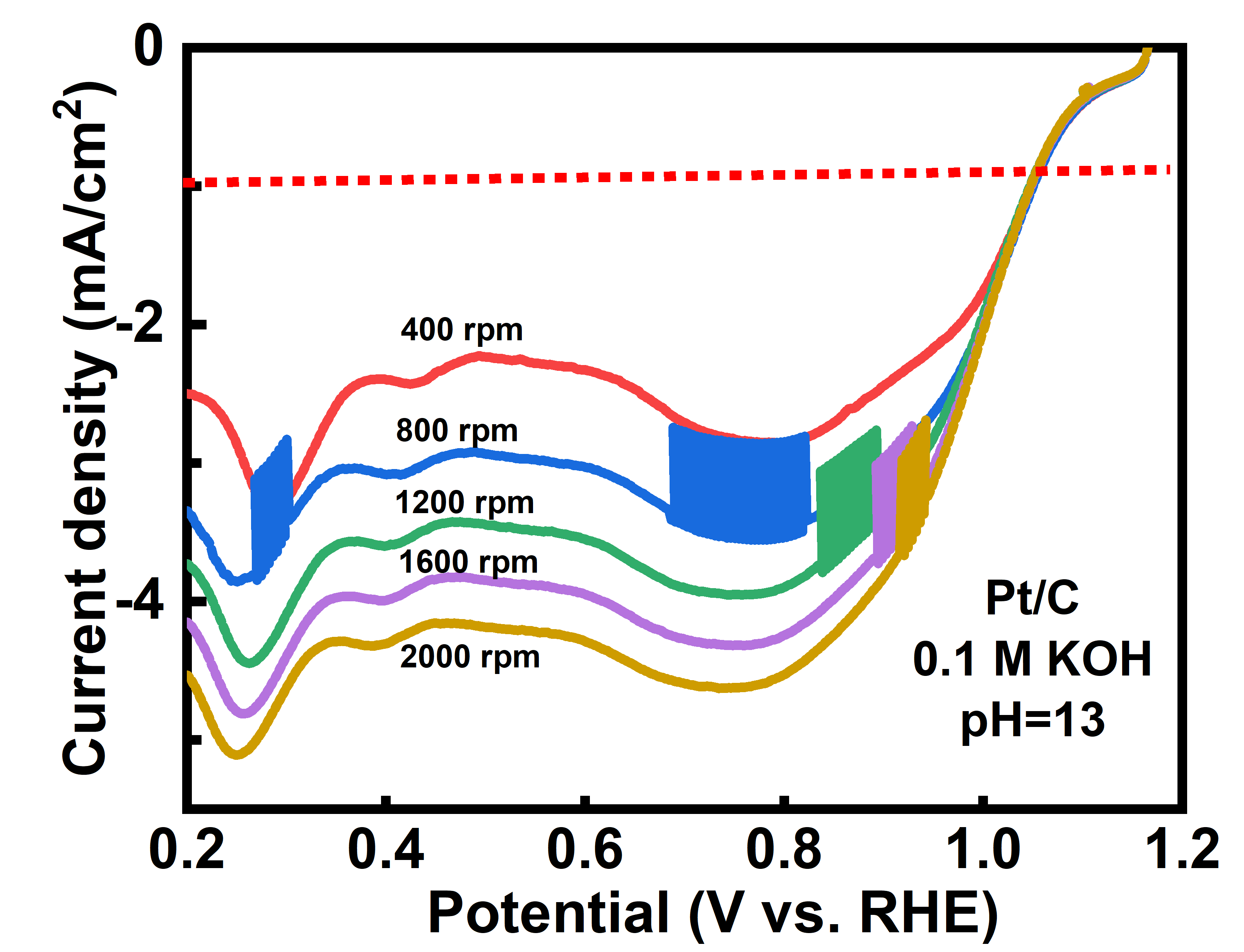

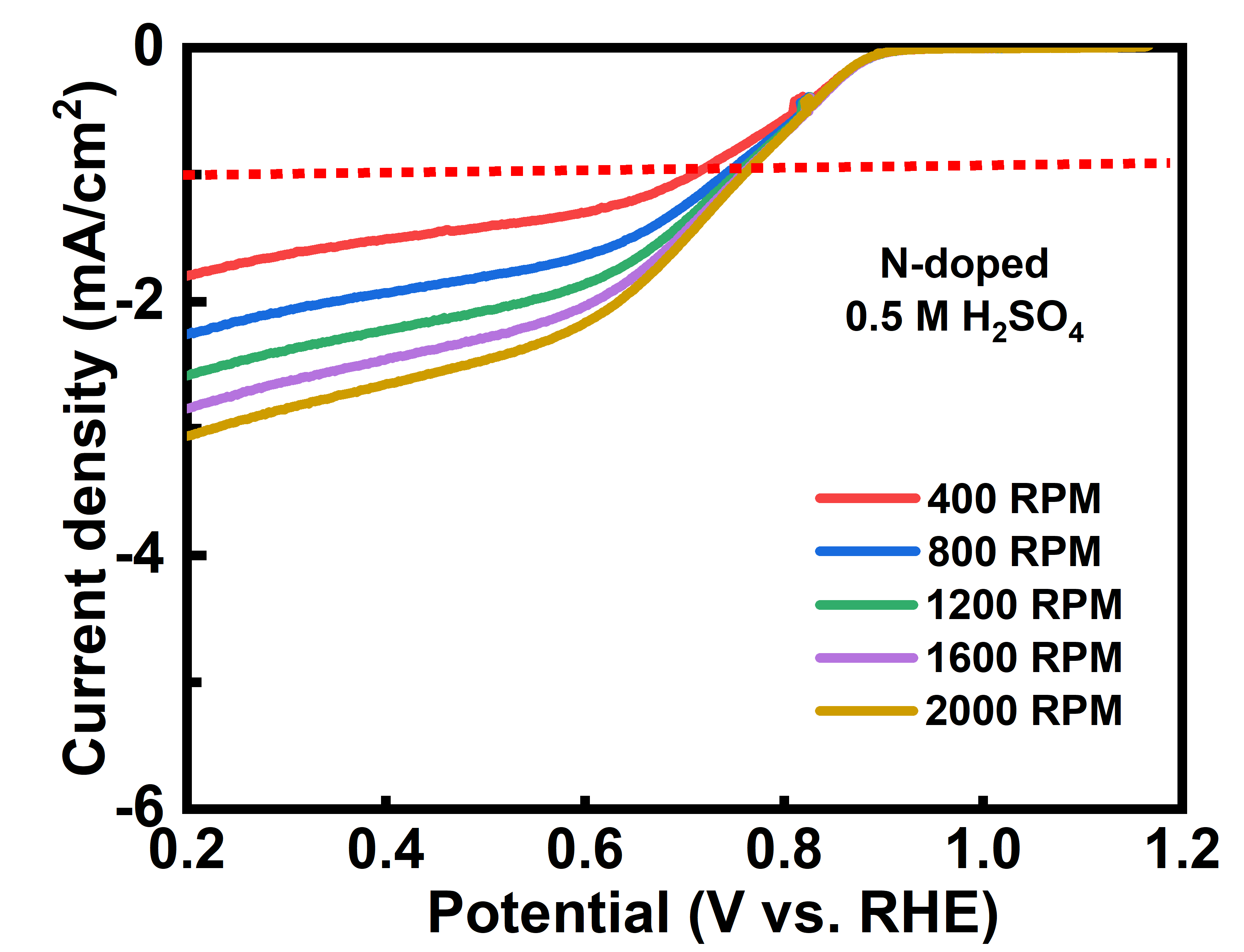

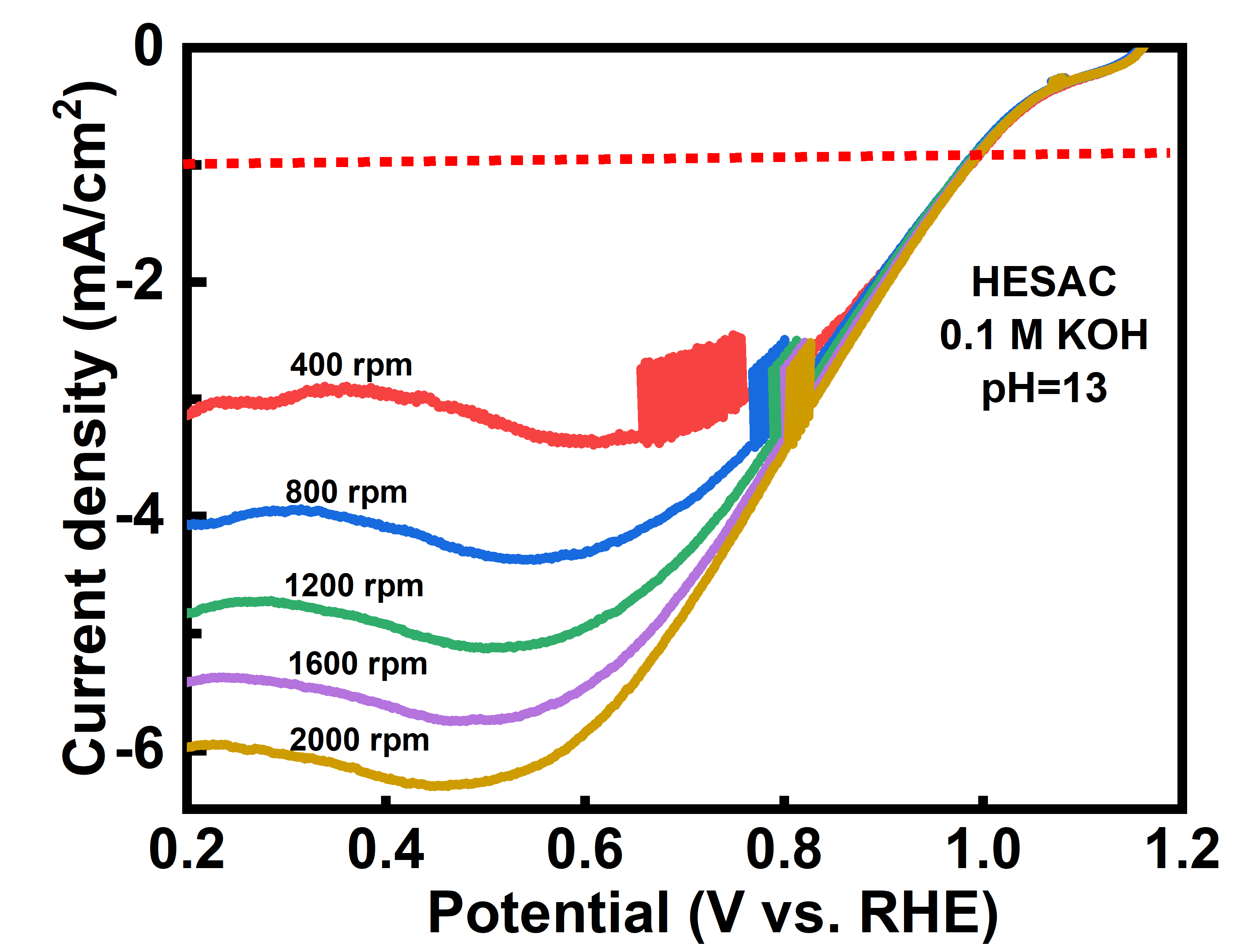

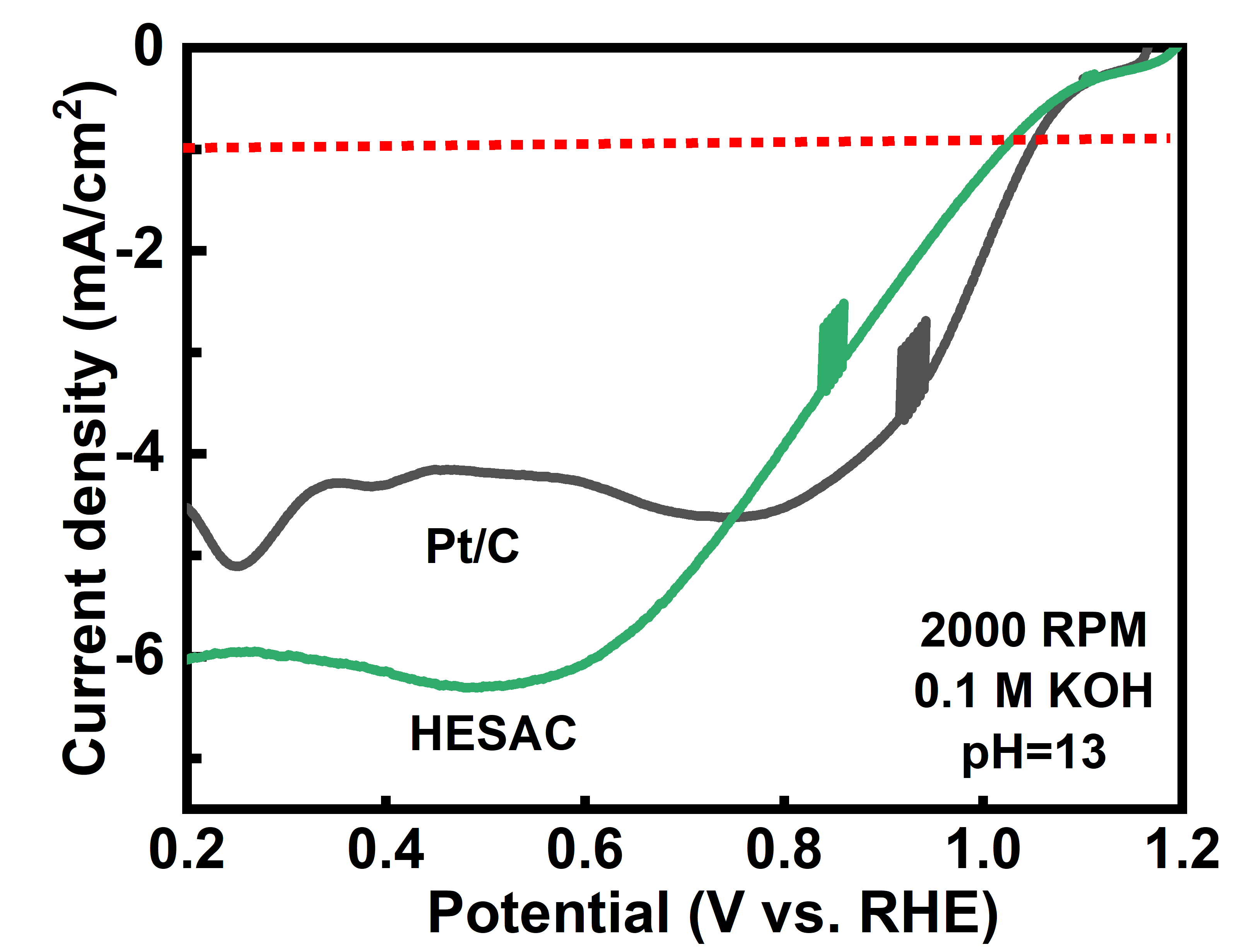


**(e)**

**(d)**

**(c)**

**(b)**

**(a)**

**Figure S34.** **ORR catalytic performance.** LSV polarization curves of Pt/C in the (a) acidic (0.5 M H_2_SO_4_) and (b) alkaline (0.1 M KOH) electrolytes for various rotation speeds (400, 800, 1200, 1600, and 2000 rpm). (c) LSV polarization curves of N-doped in the acidic (0.5 M H_2_SO_4_) electrolyte. (d) LSV polarization curves of FeCoNiRu-HESAC in the alkaline (0.1 M KOH) electrolyte for various rotation speeds (400, 800, 1200, 1600, and 2000 rpm). (d) LSV polarization curves of Pt/C and FeCoNiRu-HESAC samples in the alkaline (0.1 M KOH) electrolyte for the rotation speed of 2000 rpm.


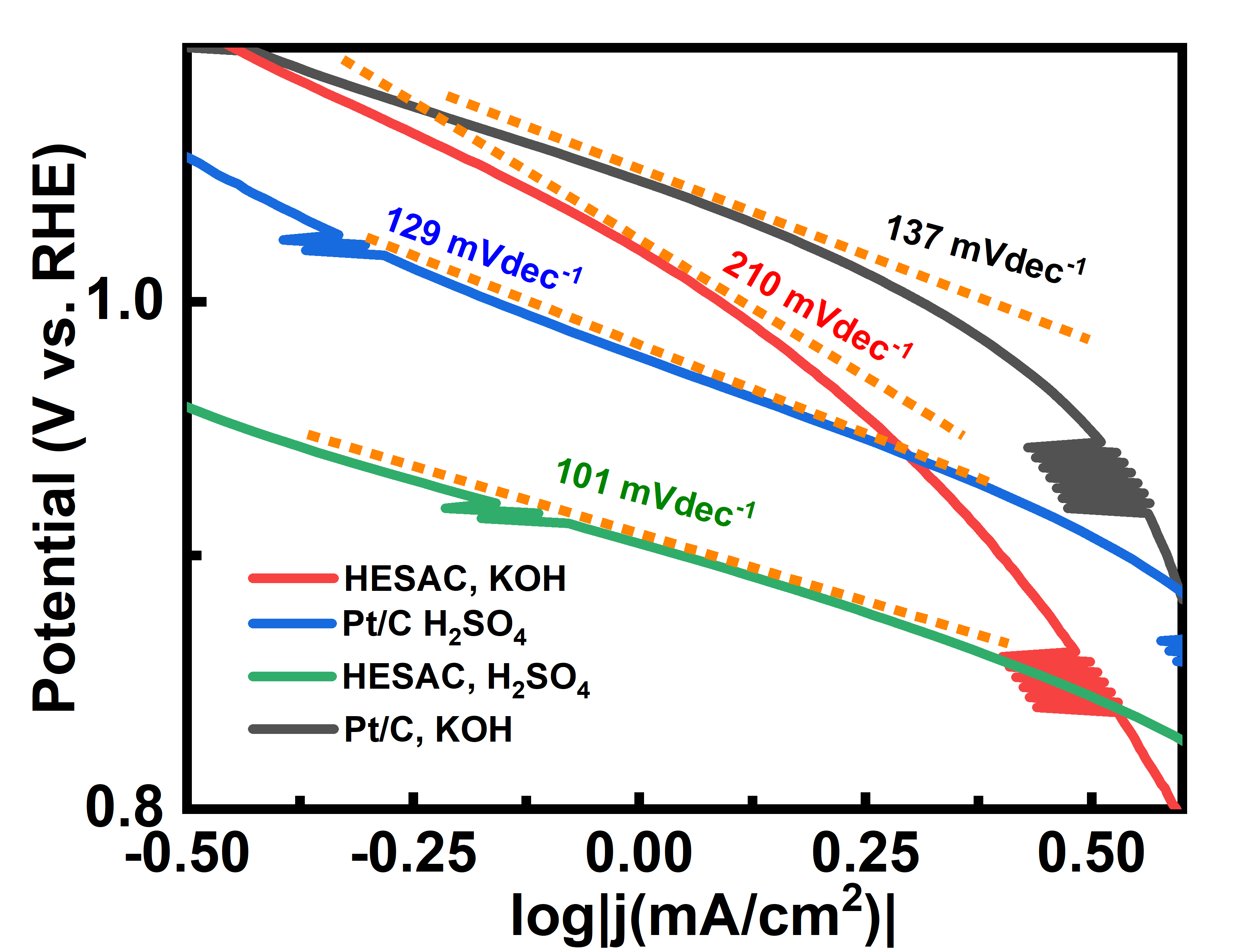


**Figure S35.** Tafel plots for FeCoNiRu-HESAC and Pt/C in the acidic (0.5 M H_2_SO_4_) and alkaline (0.1 M KOH) electrolytes at the rotation speed of 2000 rpm. The Tafel slopes are calculated at the current density of j=1 mA/cm^2^.

Based on the K-L equation, we can calculate *n* and *k*:

| $n=\frac{\beta}{0.62FC_{0}D_{0}^{2/3}v^{-1/6}} \mathrm{and} k=0.62D_{0}^{\frac{2}{3}}v^{-\frac{1}{6}}( j_{K}/B )$ | (S16) |
| --- | --- |

**Table S7.** Computed *n* and *k* from equation S16.

| **Catalyst** | ***β*** | ***j_K_ (mA/cm^2^)*** | ***n*** | ***k*** |
| --- | --- | --- | --- | --- |
| **Pt/C (0.5 M H_2_SO_4_)** | 0.28 | 7.7 | 3.16 | 0.025 |
| **Pt/C (0.1 M KOH)** | 0.21 | 9.0 | 2.37 | 0.039 |
| **FeCoNiRu-HESAC (0.5 M H_2_SO_4_)** | 0.29 | 8.2 | 3.28 | 0.026 |
| **FeCoNiRu-HESAC (0.1 M KOH)** | 0.33 | 5.3 | 3.73 | 0.015 |

**
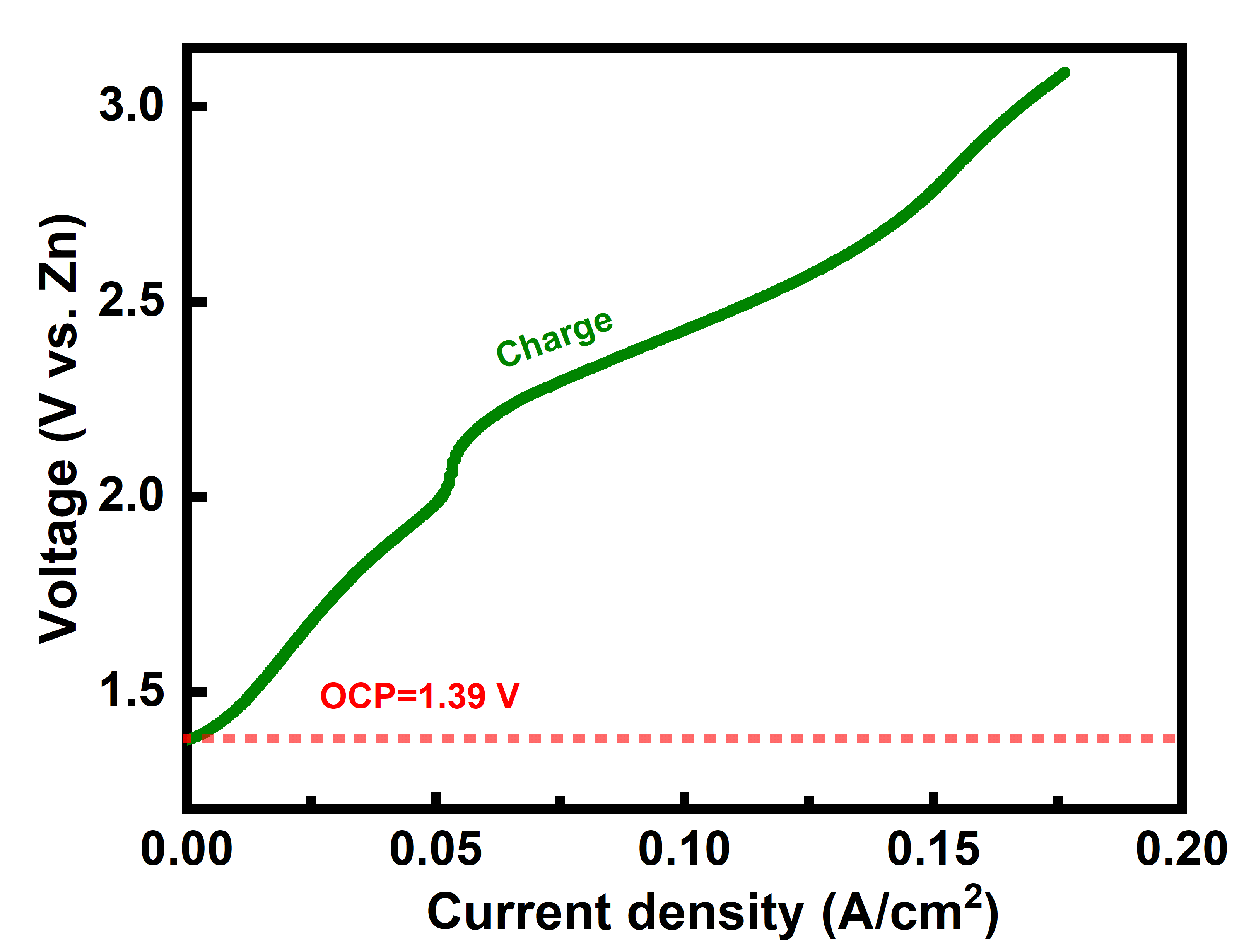
**

**Figure S36.** Polarization curve plot of Zinc-air battery assembled by FeCoNiRu-HESAC in place of cathode for charge process.


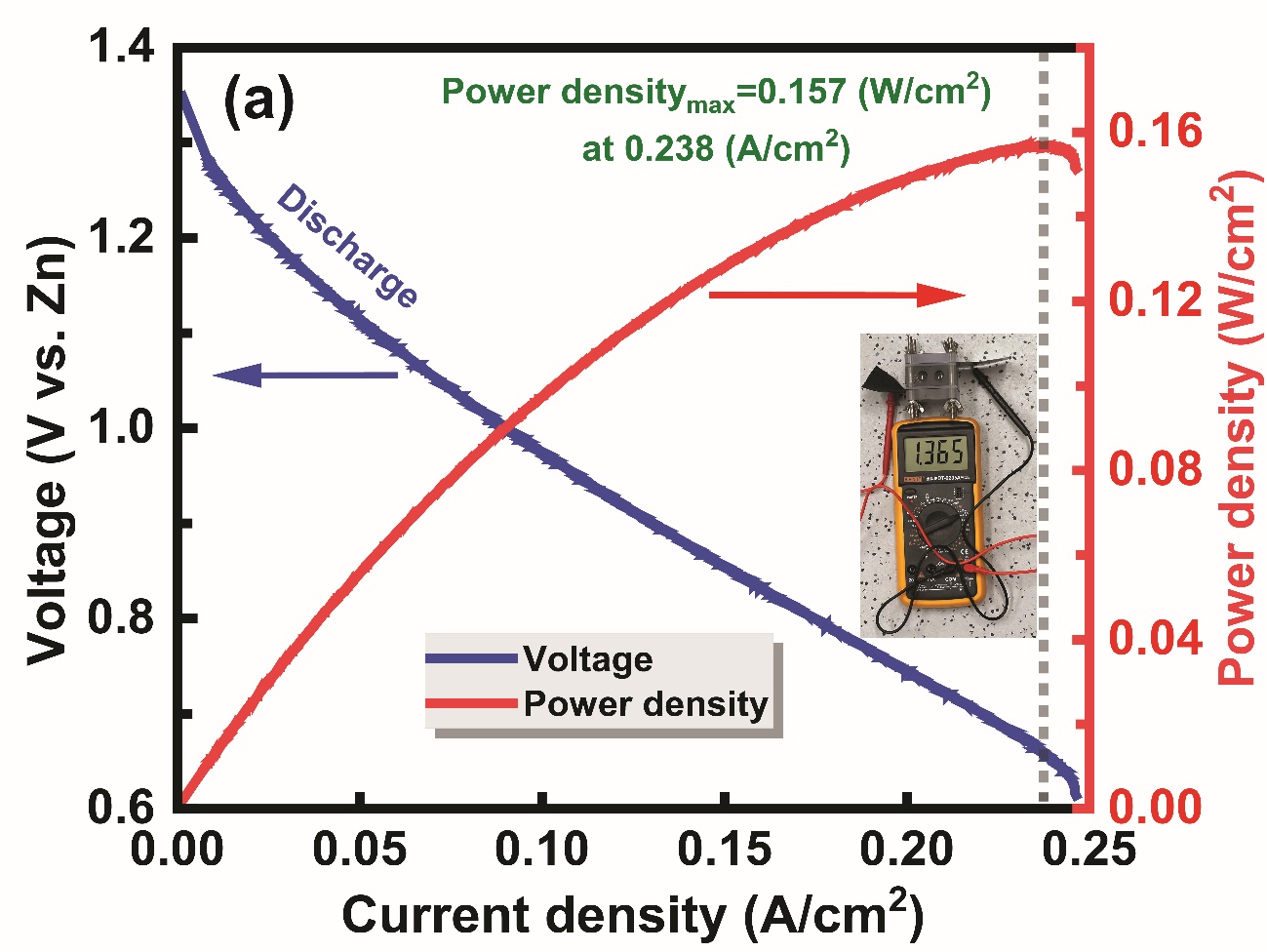


**Figure S37.** Polarization curve and corresponding power density plots of assembled Zinc-air battery for discharge process for Pt/C. The inset shows the photograph of the Zinc-air battery assembled by Pt/C in place of cathode with an open-circuit voltage of 1.365 V.


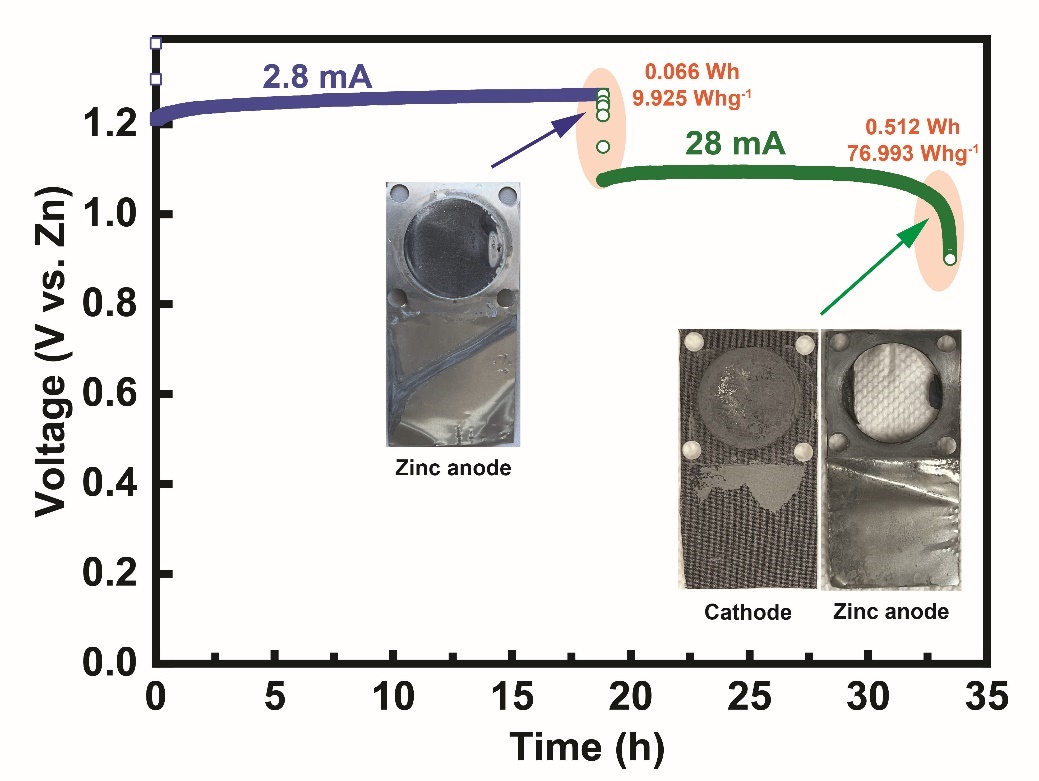


**Figure S38.** Galvanostatic discharge plots of Zinc-air battery with FeCoNiRu-HESAC in place of cathodes. The insets show the photograph of the cathode and Zinc anodes after current densities of 2.8 mA and 28 mA.


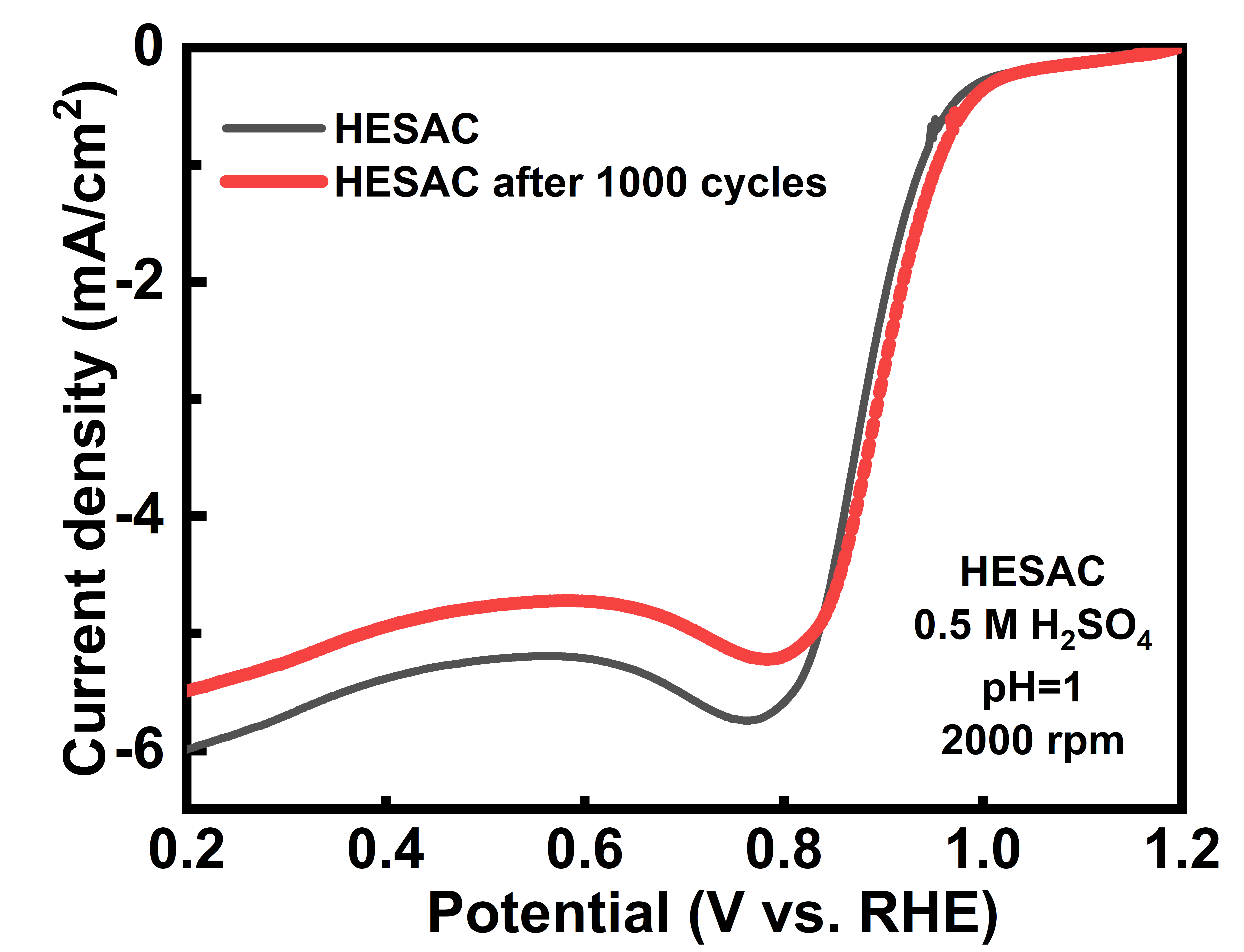


**Figure S39.** The stability test of the FeCoNiRu-HESAC sample after 1000 cycles at 2000 rpm suggesting very stable ORR performance.


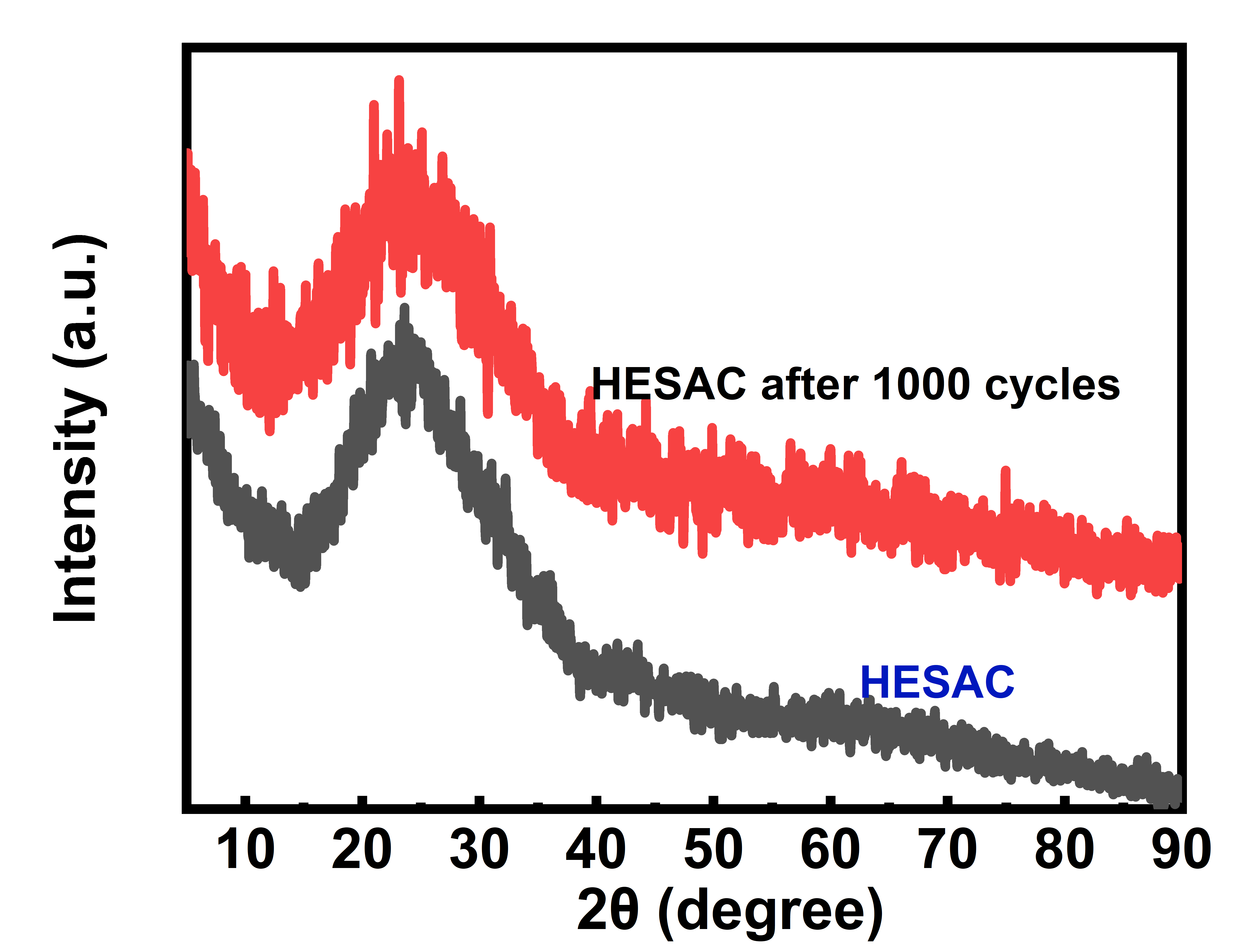


**Figure S40.** The XRD pattern of the FeCoNiRu-HESAC sample before and after 1000 ORR cycles at 2000 rpm. After 1000 ORR cycles, no peak is detected that would correspond to metal species (metal aggregation) or their compounds with oxides or nitrides. It implies that FeCoNiRu-HESAC possesses durable and stable atomically dispersed structure.

**S4. References**

[1] J. Greeley, J. K. Nørskov, *Electrochim. Acta* **2007**, *52*, 5829.

[2] CRC Handbook of Chemistry and Physics, *CRC Handb. Chem. Phys.* **2016**.

[3] F. Rehman, S. Kwon, C. B. Musgrave, M. Tamtaji, W. A. Goddard III, Z. Luo, *Nano Energy* **2022**, *103*, 107866.

[4] H. Xu, D. Cheng, D. Cao, X. C. Zeng, *Nat. Catal.* **2018**, *1*, 339.

[5] M. Tamtaji, X. Guo, A. Tyagi, P. R. Galligan, Z. Liu, A. Roxas, H. Liu, Y. Cai, H. Wong, L. Zeng, J. Xie, Y. Du, Z. Hu, D. Lu, W. A. Goddard III, Y. Zhu, Z. Luo, *ACS Appl. Mater. Interfaces* **2022**, *14*, 46471.

[6] M. Tamtaji, H. Gao, M. D. Hossain, P. R. Galligan, H. Wong, Z. Liu, H. Liu, Y. Cai, W. A. Goddard III, Z. Luo, *J. Mater. Chem. A* **2022**, *10*, 15309.

[7] A. S. Anker, E. T. S. Kjaer, M. Juelsholt, T. L. Christiansen, S. Linn Skjaervø, M. Ry, V. Jørgensen, I. Kantor, D. R. Sørensen, S. J. L. Billinge, R. Selvan, K. M. Ø. Jensen, *npj Comput. Mater.* **2022**, *8*, 3.

[8] X. Mi, B. Zou, F. Zou, J. Hu, *Nat. Commun.* **2021**, *12*, 3008.

[9] G. Panapitiya, G. Avendano-Franco, P. Ren, X. Wen, Y. Li, J. P. Lewis, *J. Am. Chem. Soc.* **2018**, *140*, 17508.

[10] K. Khan, X. Yan, Q. Yu, S. H. Bae, J. J. White, J. Liu, T. Liu, C. Sun, J. Kim, H. M. Cheng, Y. Wang, B. Liu, K. Amine, X. Pan, Z. Luo, *Nano Energy* **2021**, *90*, 106488.

[11] A. Eckmann, A. Felten, I. Verzhbitskiy, R. Davey, C. Casiraghi, *Phys. Rev. B - Condens. Matter Mater. Phys.* **2013**, *88*, 1.

[12] M. Tamtaji, Q. Peng, T. Liu, X. Zhao, Z. Xu, P. Ryan, D. Hossain, Z. Liu, H. Wong, H. Liu, K. Amine, Y. Zhu, W. A. Goddard III, W. Wu, Z. Luo, *Nano Energy* **2023**, *108*, 108218.

[13] M. Tamtaji, S. Cai, W. Wu, T. Liu, Z. Li, H. Y. Chang, P. R. Galligan, S. I. Iida, X. Li, F. Rehman, K. Amine, W. A. Goddard III, Z. Luo, *J. Mater. Chem. A* **2023**.

[14] X. Lei, Q. Tang, Y. Zheng, P. Kidkhunthod, X. Zhou, B. Ji, Y. Tang, *Nat. Sustain.* **2023**.

[15] F. Tuinstra, J. L. Koenig, *J. Chem. Phys.* **1970**, *53*, 1126.

[16] M. D. Hossain, Z. Liu, M. Zhuang, X. Yan, G. L. Xu, C. A. Gadre, A. Tyagi, I. H. Abidi, C. J. Sun, H. Wong, A. Guda, Y. Hao, X. Pan, K. Amine, Z. Luo, *Rational Design of Graphene-Supported Single Atom Catalysts for Hydrogen Evolution Reaction*, Vol. 9, **2019**, p. 1803689.

[17] O. Bunǎu, Y. Joly, *J. Phys. Condens. Matter* **2009**, *21*.
